# The behavioral repertoire of *Drosophila melanogaster* in the presence of two predator species that differ in hunting mode

**DOI:** 10.1371/journal.pone.0216860

**Published:** 2019-05-31

**Authors:** Abhijna Parigi, Cody Porter, Megan Cermak, William R. Pitchers, Ian Dworkin

**Affiliations:** 1 Program in Ecology, Evolutionary Biology and Behavior, Michigan State University, East Lansing, MI, United States of America; 2 Department of Integrative Biology, Michigan State University, East Lansing, MI, United States of America; 3 BEACON Centre for the Study of Evolution in Action, Michigan State University, East Lansing, MI, United States of America; University of Mississippi, UNITED STATES

## Abstract

The fruit fly, *Drosophila melanogaster*, has proven to be an excellent model organism for genetic, genomic and neurobiological studies. However, relatively little is known about the natural history of *D*. *melanogaster*. In particular, neither the natural predators faced by wild populations of *D*. *melanogaster*, nor the anti-predatory behaviors they may employ to escape and avoid their enemies have been documented. Here we observe and describe the influence of two predators that differ in their mode of hunting: zebra jumping spiders, *Salticus scenicus* (active hunters) and Chinese praying mantids, *Tenodera sinensis* (ambush predators) on the behavioral repertoire of *Drosophila melanogaster*. We documented three particularly interesting behaviors: abdominal lifting, stopping, and retreat—which were performed at higher frequency by *D*. *melanogaster* in the presence of predators. While mantids had only a modest influence on the locomotory activity of *D*. *melanogaster*, we observed a significant increase in the overall activity of *D*. *melanogaster* in the presence of jumping spiders. Finally, we observed considerable among-individual behavioral variation in response to both predators.

## Introduction

Predation is a ubiquitous selective force that shapes the evolution and maintenance of defensive traits in prey populations [1–6]. Predator hunting-modes, i.e., the set of behaviors that predators employ to pursue and capture their prey [7–9], have been shown to induce distinct prey responses that can have dramatic implications on the productivity of ecological communities [10]. Despite the importance of hunting mode in determining behavioral responses in prey, only recently have studies that describe the effects of predators on prey traits (e.g. [11–13]) explicitly considered the role of predator hunting-mode (e.g. [9,10]).

Here we examine variation within and between individuals in the behavioral repertoire of the fruit fly, *Drosophila melanogaster*, in response to two predator species that, among other attributes, differ in hunting modes. We used *D*. *melanogaster* because, although the species is one of the most well-studied model organisms in genetics, there is a relative paucity of information regarding *D*. *melanogaster’s* natural history, ecology and behavior, including habitat, food resources, and natural enemies (but see [14–18]).

Previous work has examined the effects of natural enemies on population and community structures of a few *Drosophila* species. For example, Worthen (1989) studied the effects of predation by rove beetles (staphylinids) on the coexistence of three mushroom-feeding *Drosophila* species [19], and Escalante & Benado (1990) showed that ant predators regulate population densities of wild *D*. *starmeri* (cactophillic fruit fly) [20]. In the species *D*. *melanogaster per se*, the role of parasites in influencing larval and adult behaviors has been extensively studied [21–23]. Despite this literature, the anti-predatory behavioral repertoire of adult *D*. *melanogaster* is not well documented.

Here, we document the influence of two predators, the zebra jumping spider (*Salticus scenicus*) and juvenile Chinese praying mantids (*Tenodera sinensis*) on the behavioral repertoire of individual *D*. *melanogaster* derived from a wild-caught population. The zebra spider is an active hunter, locating prey visually (with an extensive visual field attained by antero-medially positioned simple eyes) [24, 25]. In contrast, mantids are generally ambush predators, waiting for prey to enter their attack range [26, 27]. Despite numerous differences, such as body size (juvenile mantids are 10–15 mm, zebra spiders are 5–9 mm) and color (juvenile mantids can vary from green to brown, zebra spiders are black with white stripes), zebra spiders and juvenile Chinese mantids are similar in two relevant ways. First, both species primarily detect prey visually, and are likely incapable of depth perception when their prey item is motionless [26–31]. Second, small adult diptera (but not necessarily *D*. *melanogaster*) account for a substantial proportion of the diet of both predators in the wild. On average, diptera constitute approximately 63% and 86% of the diets of juvenile *T*. *sinensis* and adult *S*. *scenicus*, respectively [32, 33].

Under controlled laboratory conditions, we documented the behaviors of individual adult *D*. *melanogaster* with and without the two predator species. We provide ethograms for each individual *D*. *melanogaster*, and measure the change in duration and frequency of occurrence of different fly behaviors in the presence of predators. We also provide estimates of inter-individual variation in behavioral responses to predators, and describe the temporal associations among *D*. *melanogaster* behavioral sequences in the presence versus absence of the two predators. We discuss our results in terms of conditionally expressed behaviors as they relate to predator hunting mode and in terms of broadening our understanding of the behavioral ecology of *D*. *melanogaster*.

## Materials and methods

### *Drosophila* population and culture conditions

The *Drosophila melanogaster* population used in this study originated from a natural population at Fenn Valley Vineyards in Fennville, Michigan (GPS coordinates: 42°34'12.0"N 86°08'24.0"W) during the summer of 2010. A lab population (henceforth referred to as FVW) was initiated from this collection using the progeny of over 500 single-pair matings of field caught *D*. *melanogaster* as well as wild caught males. This design allowed us to screen out the sympatric congener, *D*. *simulans*, which was present in our collections at a frequency of about 5%. Screening involved setting up single pair mating in vials and discarding all lines with *D*. *simulans*-like genital morphology. After screening, ~1500 individuals were placed in a cage (32.5cm^3^, BugDorm BD43030F) to establish the FVW population. The population was maintained in this cage at an adult density ~ 3000 individuals in a room maintained at 23°C (+/- 1°C), and 40–70% RH. Adults were allowed to lay eggs in 10 bottles with 50–60 ml of a standard yeast-cornmeal food for 2–3 days. These bottles were then removed and kept in a Percival incubator (Model: I41VLC8) at 24°C and 65% RH, throughout the larval stages. All flies and larvae were maintained in a 12 hour light/dark cycle with lights on at 08:00 hours.

For the experiments, pupae were collected 24 hours before they emerged as adults. Pupae were removed from bottles using forceps and individual pupae were placed into 1.5 ml microcentrifuge tubes. Each tube was pre-filled with ~0.5 ml of yeast-cornmeal food and its cap was punctured for gas exchange. Upon emergence, adult flies were sexed visually without anesthesia and housed in these tubes in the incubator until needed for behavioral assays. Age of flies used in behavior analysis was 3–7 days. By using socially naïve flies in our assays, we were able to establish a consistent baseline of social experience among all individuals, allowing us to eliminate the potentially confounding influence of variation in social experience on behavior in *Drosophila* [34–38]

### Spiders

*Salticus scenicus* individuals were collected throughout the spring/summer of 2012 on the campus of Michigan State University. Only adults or sub-adult individuals were used in our behavioral assays (size ranging from 5–9 mm). Spiders were housed individually in vials in a room maintained at 23°C (+/- 1°C) and 30–50% RH, and fed ~5 *D*. *melanogaster* a week. Prior to use in behavioral assays, spiders were starved for 48–72 hours. This starvation time was determined based on preliminary observations, such that the spiders remained healthy, but hungry enough to pursue their prey. Each spider was used in only one behavioral assay, and a total of 33 spiders were used.

### Mantids

Egg cases for the Chinese mantid (*Tenodera sinensis*) were collected near the campus of Michigan State University and supplemented from a commercial supplier (Nature’s Control—Medford, Oregon). Mantid egg cases were stored at 4°C and transferred to 25°C and 70% RH for hatching (~6 weeks). Given the substantial changes in mantid body size across molts [39], only first instar nymphs (size range 10–15 mm) were used for experiments. Like the jumping spiders, juvenile mantids were also fed *D*. *melanogaster*. A shorter starvation period of 24–48 hours was chosen for juvenile mantids because when mantids were starved longer, they tended to be sickly and incapable of normal locomotion. Each mantid was used only once, and a total of 33 mantids were used.

### Behavioral assays

All assays were performed 1–4 hours after the incubator lights came on in the morning (08:00). Behavioral assays were recorded with an Aiptek AHD H23 digital camcorder attached to a tripod under a combination of natural and fluorescent light present in the room where the FVW population and spiders were maintained. For each predator (spiders and mantids), we recorded the behavior for each of 15 male and 15 female socially naïve, virgin flies (collected as described above). We used a chamber constructed from the bottom of a 100 x15 mm petri dish inverted on top of a glass plate with a sheet of white paper beneath to maximize the visibility of flies and predators. While the size of these chambers may constrain the ability of the fly to perform some behaviors, the fly was generally able to run, jump and fly (among other behaviors) out of attack range of both predators).

We used a repeated measure experimental design (with each individual fly as an experimental unit) to assess individual behavioral variability first without a predator and then with either a spider or mantid (but independent flies for each predator). For each assay, an individual fly was aspirated into the chamber and allowed to acclimate for ~5 minutes (duration chosen based on preliminary observations and past studies, e.g. [40]). After this acclimation period, individual flies were recorded for ~5 minutes to quantify behavioral variation without predators or disturbance. A single spider or mantid was then introduced to the chamber using a custom made funnel and behaviors were recorded for an additional 10 minutes or until capture. These observation times were chosen based on past studies [40] and preliminary data. We found that after 5 minutes of initial habituation, prolonged time in the chamber (in the absence of a predator) had no apparent effect on the behaviors exhibited by the fruit fly. As jumping spiders are voracious predators, and ~50% of the spiders captured the fly within 10 minutes of introduction into the assay chamber, we used this as an appropriate length for the duration of the predator exposure. Additionally, longer observation times may habituate the fly to the predator, such that the observed behaviors are no longer influenced solely by the presence of the predator. To account for the uneven observations times, we included “duration spent in each predator state” as a predictor variable in our statistical models (see below).

The chamber was washed with 10–30% ethanol and rinsed with reverse osmosis water after each assay to remove potential olfactory cues.

### Behaviors recorded

All *Drosophila* behaviors were categorized and analyzed as either “states” or “events”. Behavioral states have measurable duration and are mutually exclusive with other states (e.g. individuals cannot simultaneously walk and run). Behavioral events are discrete behaviors that occur instantaneously and are also mutually exclusive with each other (e.g. turning versus jumping) but not always mutually exclusive with behavioral states. For example, an individual could perform a wing display (event) while simultaneously walking (state), but it could not jump (event) while simultaneously running (state). In this study we treated flying as an event because the structure of the experimental chamber constrained flight duration. We also recorded when a fly was not visible (occluded) to the observers analyzing the video. We recorded a total of 6 discrete events and 5 behavioral states in *D*. *melanogaster* in response to predation by spiders and mantids. In order to interpret the behaviors of an individual fly in the context of predatory encounters, we designated two keys to describe the location of the predator in regard to its interactions with the fly. As flies might alter their behavior when a predator is within striking distance, we recorded predator location based on whether or not it was within striking distance of the fly. Based on pilot assays, we estimated the striking distance of the jumping spider as ~3 times the body length of the spider, and the striking distance of a juvenile mantid as ~1 times the body length of the mantid (~ 5 mm from both spider and mantid based on differences in their body length).

### Video processing

Recorded behaviors were viewed with VLC media player (version 2.0.3) and analyzed by two observers using a manual event recorder, JWatcher V1.0 software [41]. One observer (A.P.) viewed each video and verbally announced the occurrence of behaviors while the other observer (C.P./ M.C.) recorded the occurrence of these behaviors with JWatcher. Because *Drosophila* anti-predatory behaviors are often complex and occur rapidly, we analyzed all videos at 0.5X speed.

### Controlling for effects of season and disturbance

We conducted experiments with spiders between October and December 2012 and those with mantids from March and May 2013. To confirm that predator species-specific behavioral differences were not confounded with seasonal differences in behavior, we performed 6 additional assays (alternating between spider and mantid treatments) within the span of one week. Following a spider assay, the plates were cleaned with 30% ethanol and RO water before a mantid assay was conducted.

Additionally, the process of adding a predator to the arena invariably resulted in a disturbance that likely startled the fly (unrelated to the presence of a predator). To confirm that behaviors induced by this disturbance were not confounded with predator induced behavioral differences, we performed 3 control assays. Here, after 5 minutes of acclimatization without a predator (see above for more details), the arena containing the fruit fly was disturbed gently (similar magnitude of disturbance caused by the addition of a predator). For all controls, video processing and behaviors recorded were identical to mantid and spider treatments described above. See supporting information in S2 File for a detailed description of these control experiments and their results.

All behavioral observations were conducted on either a naïve fly in isolation, or on a naïve fly in the presence of a predator. However, harmless heterospecifics also likely influence the behavioral repertoire of any organism (i.e. neophobic responses). This study does not control for behavioral changes in *D*. *melanogaster* that result from encounters with a harmless heterospecific. Thus, changes in the behavioral repertoire of *D*. *melanogaster* in the presence of either predator may, in part, reflect such an encounter.

### Data processing and statistical analysis

A custom Python script was used to parse Jwatcher formatted data files into a comma-separated-value (CSV) file for analysis in **R** (version 3.0.1). As our primary experiment was designed to utilize repeated measures, we analyzed the data as follows. To analyze the effects of predator state (i.e., presence or absence of predator) on the time dedicated to locomotory behavioral states, and number of occurrences for behavioral events, we fit generalized linear mixed effects models (using both glmer function in the lme4 package version 1.0–5, and the MCMCglmm function in the MCMCglmm package version 2.17) with predator state, total duration of assay with and without a predator (duration), sex, temperature and recording time as fixed effects, and individual by predator state and date as random effects. Formally, the model was:

$$y∼\beta_{0}+\beta_{1}PS+\beta_{2}D+\beta_{3}Ag+\beta_{4}T+\beta_{5}ST+\beta_{6}Sx+\beta_{7}+\varepsilon$$

Where **y** is a vector of time spent in a behavioral state. β_1_ is the regression coefficient for predator state, β_2_ is for duration in each predator state, β_3_ is for age of the fly, β_4_ is for temperature, β_5_ is for time at which assay was started, β_6_ is for sex of the fly and β_7_ is for date on which the assay was performed. We also estimated the model using log (duration) as an offset, with largely similar results. We estimated random effects for individuals including variation in response to predator state and duration of assay, and we fit an independent random effect for date. Thus we fit a repeated effects (longitudinal) mixed effects model allowing for variation among individuals for the influence of predator presence and duration of assay where for the i^th^ individual

$$\left(\begin{array}{c} \beta_{0i}\\ \beta_{1i} \end{array}\right)∼MVN\left([\begin{array}{c} \mu_{\beta_{0}}\\ \mu_{\beta_{1}} \end{array}],[\begin{array}{cc} \sigma_{\beta_{0}}^{2} & \sigma_{\beta_{0}{,\beta }_{1}}^{2}\\ \sigma_{\beta_{1},\beta_{0}}^{2} & \sigma_{\beta_{1}}^{2} \end{array}]\right)$$

and (independent of the above)

$$\beta_{7}∼N(0,\sigma_{j}^{2})$$

where j = 1 … date.

Preliminary analyses were inconsistent with the need to fit higher order interactions among fixed effects, so interaction terms were not considered further. The one exception was for “stopping” behavior where individuals almost exclusively performed this in the presence of the predators. For the behavioral states (locomotion, grooming and stopping) we assumed normally distributed variation. For the counts of events (abdominal lift, jumping, etc) we used a log-link function and assumed the variation was Poisson-distributed. Estimation using both maximum likelihood (lmer) and simulating the posterior distribution (MCMCglmm) provided similar results for fixed effects, and generally for random effect components as well. For any fixed amount of time (i.e., bounded response), examining multiple behaviors means that more time spent in one behavior leaves less time for others. While each of the behavioral states and events was fit to independent models, it is important to note that these results are not independent of each other. However, our attempt at a multivariate approach was unsuccessful and did not result in convergence, likely due to the large number of behaviors analyzed. Because our primary goal was to describe and quantify all *D*. *melanogaster* behaviors in response to predation risk, we did not collapse behaviors into fewer categories that would be conducive to multivariate analyses. Therefore, given the use of fixed observation periods, covariation due to length of time in the assay is a potential issue and may influence the magnitude of estimates for highly correlated behaviors.

Among individual coefficients of variation were calculated by dividing the square root of the among individual variance component from the model by its respective fixed effect estimate (i.e. its “mean”). While confidence intervals were consistent for fixed effects, the intervals were more difficult to estimate given the complexities of the random effect structure of the model, and some caution is warranted for their interpretation.

To test for non-random associations in the temporal structure of behavioral patterns we constructed transition frequencies using the “msm” library (version 1.2) [42] in **R**. To test for first order Markov processes between behaviors (transition probabilities), as well as the influence of predator presence on these transition probabilities, we fit log-linear models (assuming Poisson distributed data) with the transition frequency matrices [42] using glm in **R**. As advocated in [43, 44] we fit a saturated log-linear model (with lag0, lag1 and predator state as the effects in the model) and tested the influence of deleting the terms (i.e. third order interaction) on change in deviance. We used modified “Z-scores”, adjusted using sequential Bonferroni to assess the deviation of particular cells in the transition frequency matrix from expected values (assuming independence). For the visual transition probability matrices, we combined the behavioral event “pause” with the behavioral state “stop” because 1) we wanted to reduce the complexity of the matrix and 2) the main difference between the two behaviors is that pause is instantaneous and stop has duration. All transition diagrams were constructed in Inkscape (version 0.48.2 [45]).

## Results

From pilot observations (not included in analysis), we (I.D., A.P. and C.P.) catalogued and described *Drosophila melanogaster* behaviors observed in the presence of a predator (Table 1). Among the behaviors listed in Table 1, abdominal lifting, stopping, and retreat (Videos 1, 2 and 3 in S2 File), may be interesting in the potential context of capture deterrence as they were performed following direct encounters with the predators. However, in this study we did not directly assess function of these behaviors. It is important to note that the “stop” behavior has duration whereas the “pause” behavior is instantaneous.

**Table 1:** Names and descriptions of all examined behaviors.

Behavior	Description
Abdominal lift (ab)	Momentary rearing up on abdomen http://dx.doi.org/10.6084/m9.figshare.1185638
Fly	Moving through space by wing use
Jump	Instantaneous movement between points without wing use
Pause	Noticeable period of inactivity—identical to stop but transitional, duration < 1s
Turn	180 degree change in orientation without change in position
Wing display (wd)	Momentary lifting up of wings without singing or vibration
Groom	Running legs over any body part-often while otherwise stationary
Walk	Movement through space by ambulation
Run	Rapid movement through space by ambulation
Stop	Immobile with duration > 1s http://dx.doi.org/10.6084/m9.figshare.1185639
Retreat	Walking in reverse upon encounter with an object (like a predator) http://dx.doi.org/10.6084/m9.figshare.1185640

Video links are also provided at the end of S2 File.

### Flies perform a range of anti-predatory behaviors in response to zebra spiders and mantids

**Fig 1:**
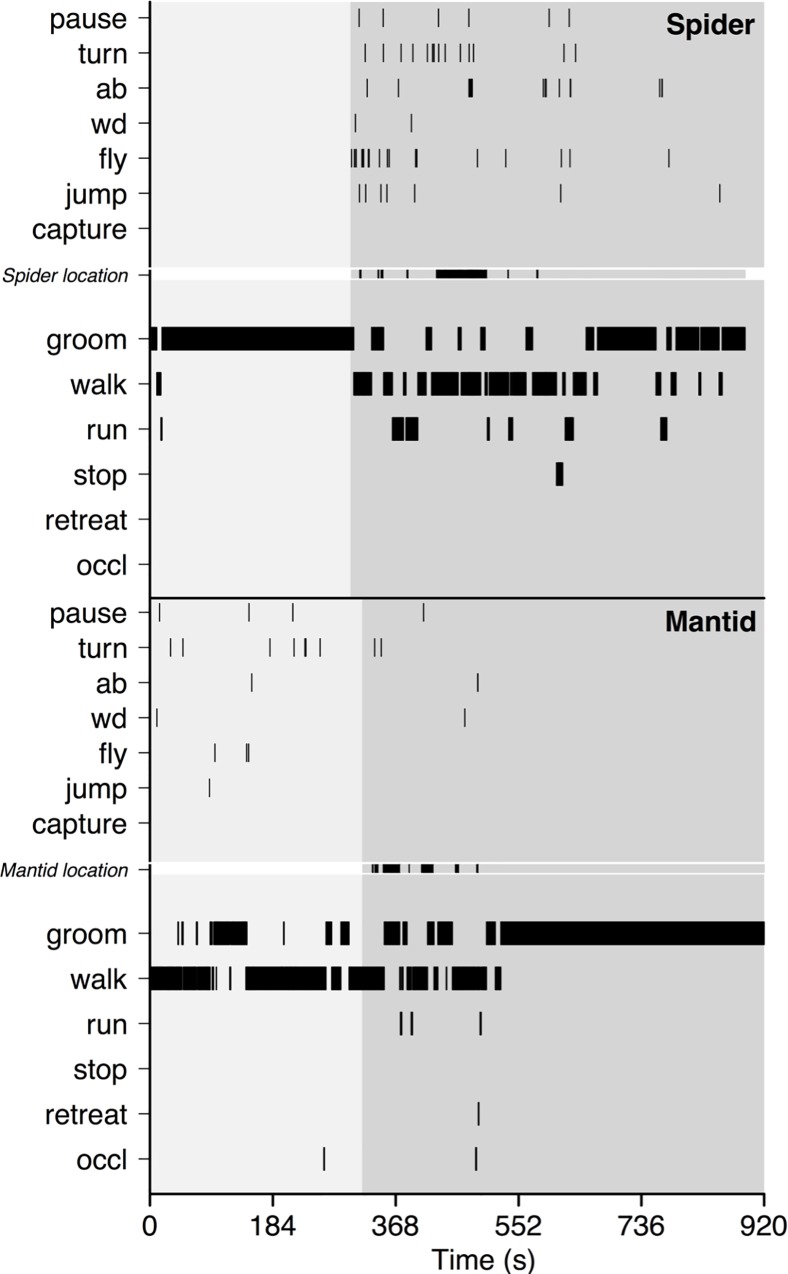
Observed behavioral changes in *D*. *melanogaster* following the introduction of jumping spider or juvenile mantid. Top panel: Representative ethogram of a male, 4 day old *D*. *melanogaster* in response to a zebra jumping spider. Bottom panel: Ethogram of a male, 5 day old *D*. *melanogaster* in response to a juvenile Chinese praying mantid. Light grey background represents time in the arena before the addition of a predator and dark grey background is when the predator was present in the chamber. Each black bar represents the occurrence of a behavior during the observation period. Top half of each panel (separated by *Predator location*) consists of events and the bottom half consists of states. Because states have duration, the width of each black bar corresponds to the duration of a state. *Predator location* (i.e., *Spider location* in top panel and *Mantid location* in bottom panel) indicates whether the predator was within striking distance of the fruit fly at that time point. This information is relevant only after the predator was added to the chamber (~ 300 s into the assay). Dark grey bars in *Predator location* indicate that the predator was within striking distance and light grey regions indicate that the predator was out of striking distance. *Predator location* is white when the predator is absent from the arena or after successful capture. If capture did not occur, *predator location* remains light grey in color.

**Fig 2:**
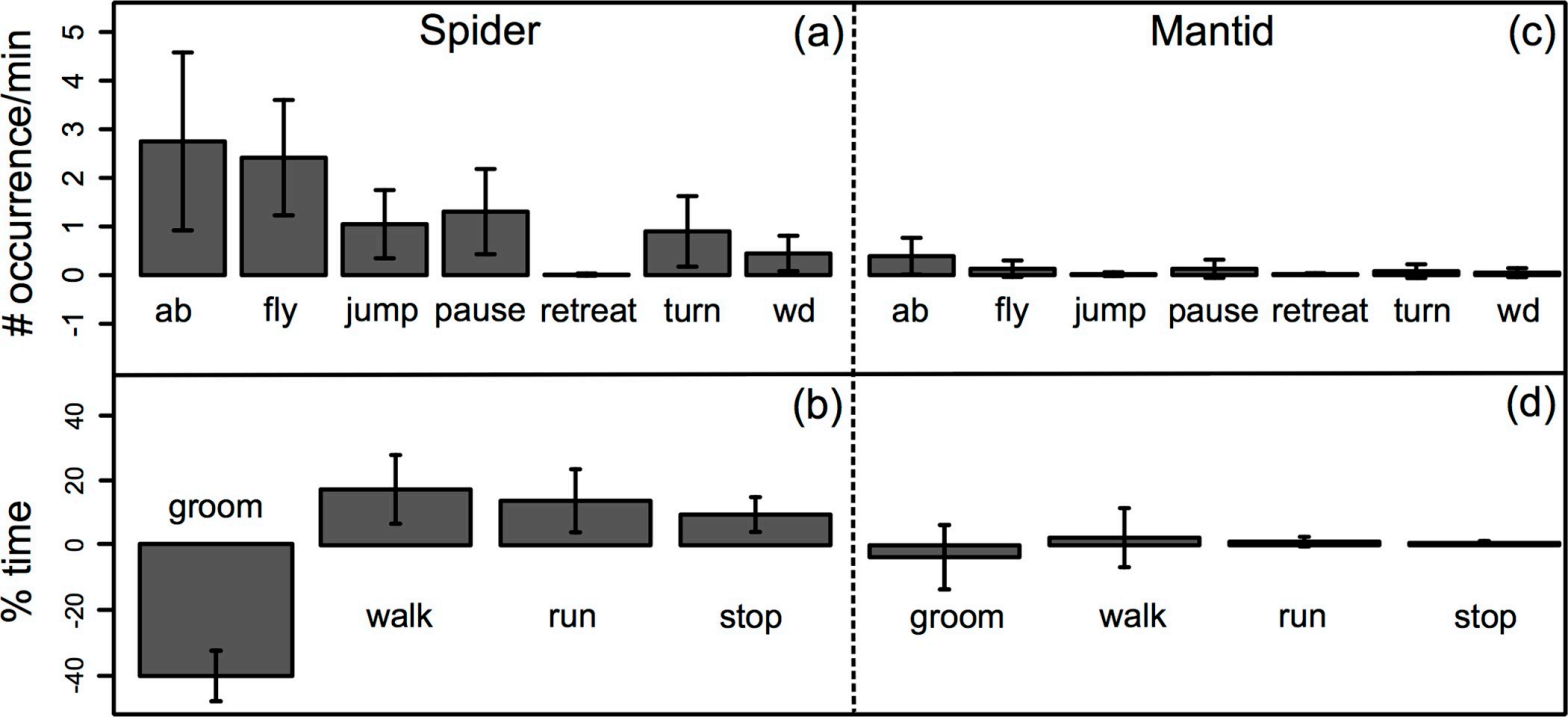
Fruit flies increase overall activity levels in the presence of jumping spiders but not in the presence of mantids. Plots (a) and (c) show change in mean number of occurrences per minute of each behavioral state as a result of the addition of a predator. Plots (b) and (d) show mean change in percentage of total time spent in a given behavioral state caused by the addition of a predator. On the left of the dotted line, behavioral changes correspond to the presence of a spider, whereas on the right of the dotted line, behavioral differences are due to the presence of a juvenile praying mantid. Error bars are ± 95% CIs. Results shown here are made from raw data and do not reflect estimates derived from the mixed effects models described in the results section. ab = abdominal lifts, wd = wing display.

Ethograms representing the response of each individual fruit fly to the presence of a zebra jumping spider are shown in Fig 1A and S1 File. The presence of a jumping spider had substantial effects on both the behavioral states and events displayed by *D*. *melanogaster* (Fig 2). According to our statistical models, when a spider was present, *D*. *melanogaster* increased the time it spent walking and running by 50% (95% CI: 21–79% increase) while grooming 60% less (95% CI: 43–77% decrease). This is shown in Fig A in S2 File. While they were observed at low frequencies prior to the addition of spiders, *D*. *melanogaster* substantially increased the frequency of pauses, jumps and flights (per minute) in the presence of spiders (Fig 2 and Fig C in S2 File). Our models estimate that the frequency of abdominal lifts increased from 0.71/minute to 2.63/minute (95% CI for increase: 1.23–5.91), while jumping showed a ~7X increase in frequency from 0.09/minute to 0.65/minute (95% CI for increase: 0.32–1.78). “Stopping”; a motionless state that likely aids in capture avoidance (see Videos 2 in S2 File) was not performed by *D*. *melanogaster* in the absence of spiders (Fig B in S2 File). However in the presence of spiders, the average total time spent “stopping” increased to ~25.8 seconds (95% CI: 10.1–41.7 seconds). When interacting with spiders, flies were only observed to perform the “retreat” behavior once (out of 30 individuals). Interestingly, we did not see significant sex specific differences in either frequencies of occurrence or time allocated (Fig A and C in S2 File) to the majority of measured behaviors.

In contrast to their behavior in the presence of jumping spiders, the presence of a juvenile mantid had minimal influence on *D*. *melanogaster*’s locomotory activity (Fig 2C and 2D, also see ethograms in Fig 1B and S1 File). Time spent grooming, walking, running and stopping was highly variable (with estimated changes with the predator including zero) in the presence of a juvenile praying mantid (Fig 2D and coefficient plots from generalized linear mixed effects models are shown in Fig A and B in S2 File). Statistical models show that grooming decreased by 20% (95% CI: 83% decrease to 36% increase), locomotion increased by 9% (13% decrease to 32% increase) and stopping increased by 40% (8% decrease to 85% increase). Similarly, the presence of a mantid did not influence the frequency at which *D*. *melanogaster* tended to perform most instantaneous behaviors (fly, pause, wing display, turn, and jump; Fig 2C and Fig D in S2 File). However, as was observed in the presence of spiders, flies substantially increased the frequency of abdominal lifting, from 0.13/minute to 0.40/min (95% CI: 0.14–1.41) in the presence of a juvenile mantid (results from generalized linear mixed effects model, Fig D in S2 File). Upon encounter with a mantid, half of the individuals (15/30) performed a locomotory reversal behavior (Video 3 in S2 File), which we term “retreat”. As with the zebra spiders, we saw no significant sex specific differences in response to mantids.

### Individuals varied greatly in their response to predators

**Fig 3:**
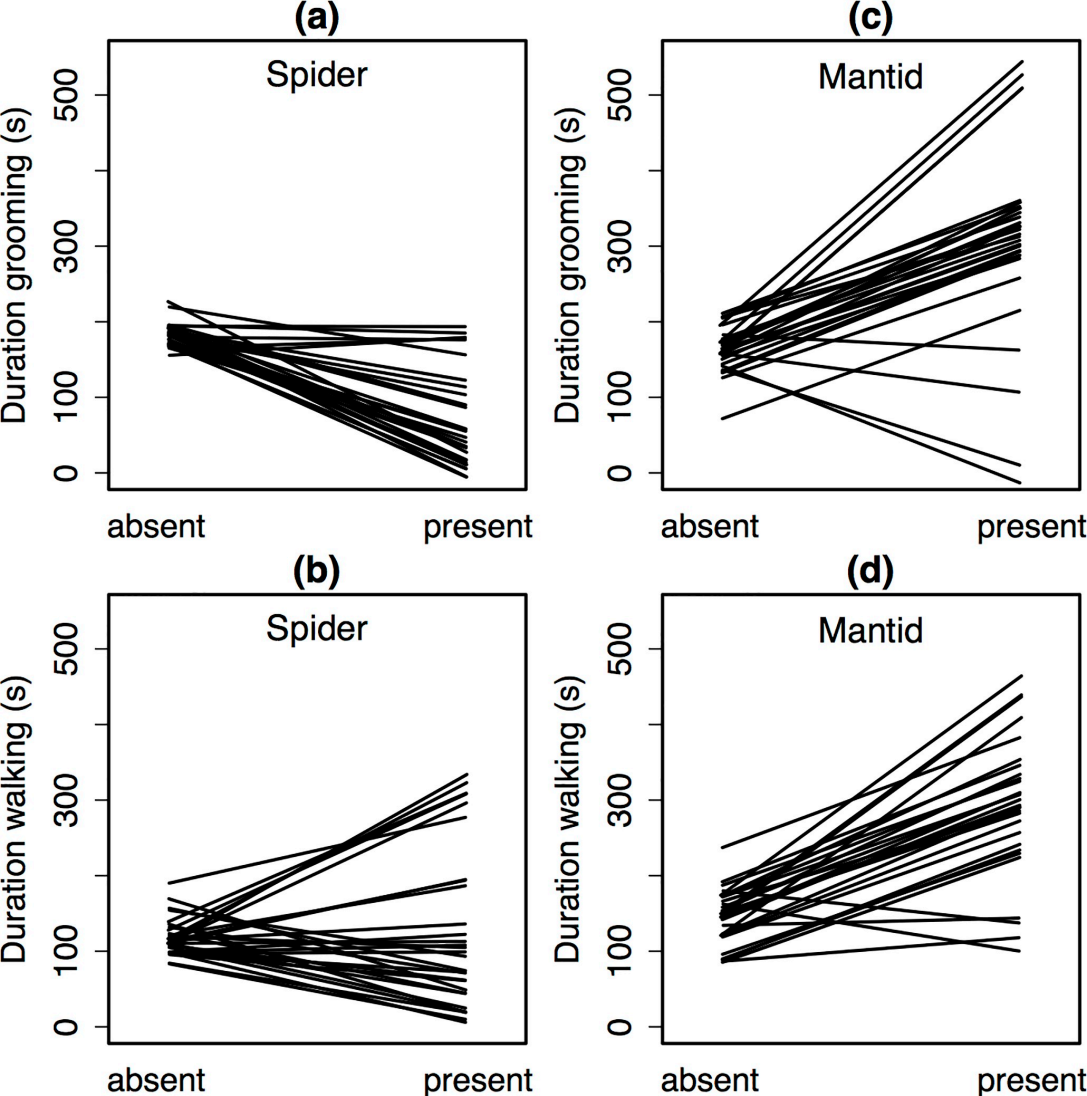
Among-individual behavioral variation in response to predators. Reaction norms visualize how each individual fruit fly responded to the introduction of a spider (panels a and b) or a mantid (panels c and d) into the assay chamber. Measures are in seconds. Each line corresponds to response of one individual. Estimates are derived from the predicted values for each individual from the mixed models.

Given our experimental design, we were able to model the degree to which individuals varied in their responses to the jumping spiders and juvenile mantids. Individuals varied greatly both in their baseline activity levels as well as in their propensities to respond to jumping spiders. The among-individual coefficient of variation for time spent grooming in the absence of predators was 57.7% (40.1–74.2%). While most individuals reduced their grooming activity in the presence of jumping spiders, the degree to which they did so varied considerably, with the among individual coefficient of variation for the decrease being 67.8% (26.5–94.6%; Fig 3A). For walking, the among-individual coefficient of variation was 80.3% (50–105%) in the absence of the spider, and 135% (1–181%) for the magnitude of increase in the presence of the spider (Fig 3B). Performance of the stopping behavior by *D*. *melanogaster* in the presence of spiders also varied among individuals, with the among-individual coefficient of variation being 168% (95% CI: 123–214%). This is driven in part by the fact that 40% of individual flies never performed stopping, even in the presence of the spider. There was a negative correlation (-0.84), between the amount of time individuals spent grooming before and after the addition of the spiders (Table 2). That is, on average, individuals who were more active prior to the addition of the spider reduced their activity to a greater extent in the presence of the spider. A similar negative correlation (-0.66) for among individual activity for locomotion was observed (Table 2).

**Table 2:** Individual flies show negative correlations between behavioral states before and after the introduction of a predator.

Grooming, Spider			
	**Intercept**	**Pred.State**	**Time**
**Intercept**	89.2 (62.0, 115.3)	-68.2 (-88.7, -34.1)	26.4 (14.0, 36.0)
**Pred.State**	-0.84	61.7 (18.8, 84.5)	-14 (-25.5, 17.1)
**Time**	0.75	-0.3	10.4 (4.4, 14.9)
**Walking, Spider**			
	**Intercept**	**Pred.State**	**Time**
**Intercept**	81.9 (43.3, 109.8)	-60.2 (-89.5, 27.5)	25.6 (-8.8, 36.7)
**Pred.State**	-0.66	67.3 (0.36, 98.5)	11.5 (-22.2, 29.2)
**Time**	0.62	0.15	13 (5.6, 18.2)
**Grooming, Mantid**			
	**Intercept**	**Pred.State**	**Time**
**Intercept**	122.8 (59.6, 175)	-20.2 (-117, 106)	46.0 (-16.9, 74.5)
**Pred.State**	-0.05	60.5 (0.13, 100.2)	-18.3 (-47.9, 34.0)
**Time**	0.8	-0.26	21.5 (2.6, 33.8)
**Walking, Mantid**			
	**Intercept**	**Pred.State**	**Time**
**Intercept**	144.8 (86.2, 198)	-100.3 (-162.6, 38.8)	63.2 (31.6, 90.3)
**Pred.State**	-0.86	80.5 (0.21, 139.3)	-45.2 (-76.4, 19.6)
**Time**	0.94	-0.86	29.4 (11.3, 43.5)

There is considerable variation among individuals in time spent performing specific behaviors (i.e. walking and grooming), with and without predators. However, there is a strong negative correlation within individuals for time spent before and after introduction of the predator. That is, individuals who spend more time performing a specific behavior prior to the addition of a predator, reduce that behavior to an even greater amount (than the average for the sample) once the predator is introduced. The one exception is for grooming for the mantid trials. Diagonals of the table (light grey background) contain the standard deviation (mean of the posterior distribution) for individual behavioral responses (95% CIs in parentheses) from the random effects of the models. Above the diagonal (white background) are covariances between predictors (and CIs in parentheses). Below the diagonal (dark grey background) are correlation coefficients for the covariances between the predictors.

Although the presence of a mantid had only a small average effect on fly behavior, flies did vary considerably among individuals in their grooming and walking activities. Indeed, the among-individual variability in proportion of time spent grooming and walking is greater in magnitude in the presence of the mantids than spiders (Fig 3). Evidence for negative co-variation for intra-individual behavior before and after the addition of the predator was not strongly supported (i.e. 95% CIs for covariances included zero) (Table 2).

### Predators influence the temporal associations among behavioral sequences

**Fig 4:**
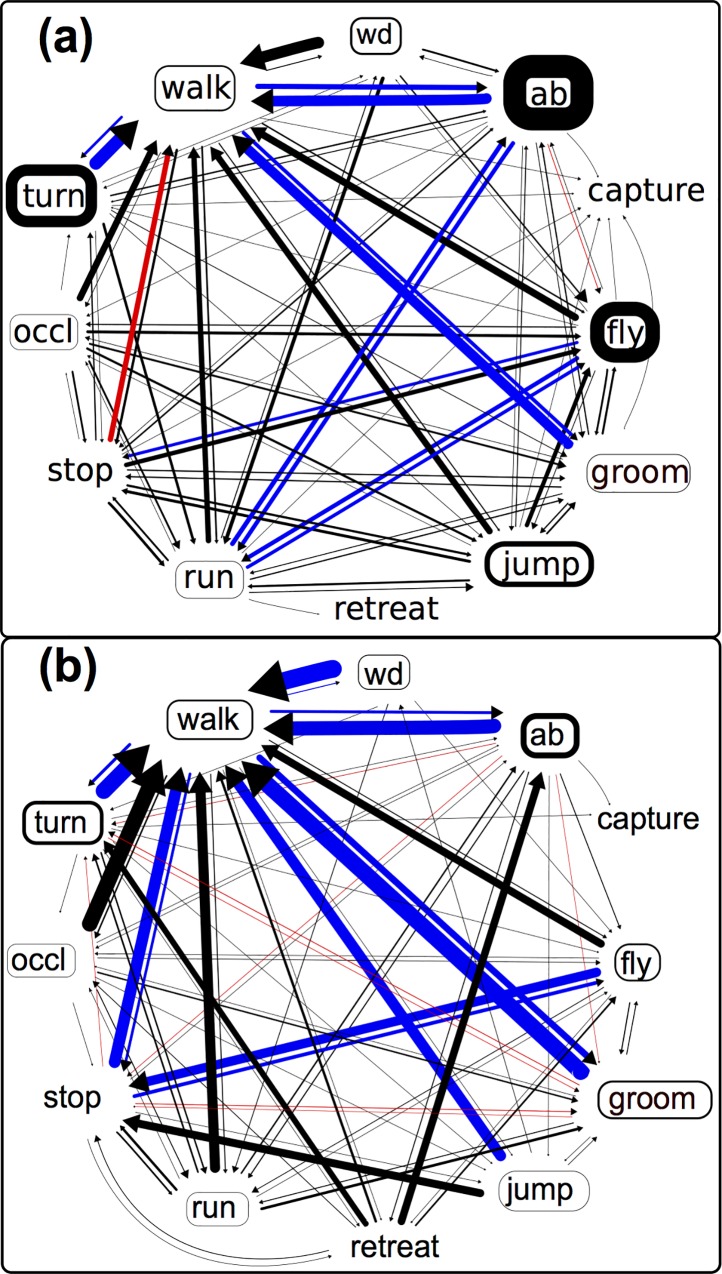
Spiders and mantids had different effects on the temporal associations between pairs of *D*. *melanogaster* behaviors. a) A diagram representing probability of transitioning from one fly behavioral state to the other in the presence of a zebra jumping spider. b) A diagram representing probability of transitioning from one fly behavioral state to the other in the presence of a juvenile praying mantid. Thickness of arrows indicates transition probability between the two behaviors. Blue arrows represent transitions that occurred with a higher probability than expectation, red arrows represent transitions that occurred with a lower probability than expectation, whereas black arrows represent transitions that occurred at expected probabilities. Transition probabilities are obtained by dividing each transition frequency (see Tables A and B in S2 File) between a pair of behaviors by the total number of times a given behavior was performed (row sums in Tables A and B in S2 File). The arrowhead points to the behavior being transitioned to. Thickness of the box around behavioral state (groom, run, occl, retreat, stop and walk) indicate the mean proportion of total time spent in that behavior, whereas thickness of the box around behavioral events (fly, jump, turn, wd, ab) indicates mean number of occurrences per minute of that behavior. To reduce the complexity of the web we combined the behaviors “pause” with the behavior “stop”. Behavioral transitions that occurred less than 10 times have not been shown in the figure.

To visualize the temporal associations among behavioral sequences, we constructed transition matrices (Tables A, B, E and F in S2 File) and transition probability diagrams for all pairs of behaviors in the presence (Fig 4A) and absence (Fig G in S2 File) of predators.

In response to jumping spiders, transitions among behaviors are somewhat more dispersed (with many connections between behaviors), suggesting that there is weak temporal association between fruit fly behaviors. Indeed these qualitative conclusions are supported based on the Z-scores. In the absence of spiders, 8 possible transitions were significant (after controlling for multiple comparison, Table B in S2 File), while 13 transitions were significant in the presence of the spider (Table A in S2 File). Most of these differences were due to the increase in behaviors potentially involved with anti-predation activity (i.e. flight, abdominal lifting). However, while the results of the log-linear analysis (across the whole transition frequency matrix) supported the dependence of current behavioral states on the previous state (resid df = 71, deviance = 632, p < 0.001), the inclusion of predator status did not influence this dependence (resid df = 71, deviance = 59, p = 0.8).

Transition matrices and transition probability diagrams (Fig 4B, and Fig G, Tables C, D, G and H in S2 File) show patterns of temporal association among behaviors. In response to juvenile mantids, behavioral transitions are less “dispersed” than those in the presence of jumping spiders (Fig 4). While most behaviors (abdominal lift, fly, groom, jump, run, stop, and turn) tend to transition to walking, we also see stronger associations between other pairs of behaviors. For example, after performing the retreat behavior, flies often either performed the abdominal lift or turn, while flight was often followed by stopping. These observations are supported by the findings that in the absence of mantids, 12 transitions showed significant deviations from expectations (Table D in S2 File). In comparison, in the presence of mantids 23 transitions showed a significant deviation from expected values (Table C in S2 File). Interestingly, as with the spiders the log-linear model supports the non-independence of behavioral states (resid df = 71, deviance = 1054, p <0.001), but not for the additional influence of predator state on this non-independence (resid df = 71, deviance = 72, p = 0.4).

## Discussion

Prey organisms can alter their behavior to reduce the likelihood of encounter, detection or capture with a predator [46]. For example, when predators are present, ground squirrels dedicate more time to vigilance behaviors (like scanning for a predator [47]) and some aquatic insects spend more time in refuges [48]. These changes in behavior may alter the use of resources, and potentially the fitness of an organism. In this study, we describe the behavioral repertoire of *Drosophila melanogaster* in response to predation by the zebra jumping spider (*Salticus scenicus)* and juvenile Chinese praying mantids (*Tenodera sinensis*). Among other characteristics, zebra spiders and praying mantids differ in their hunting mode. While we discuss our findings with respect to hunting mode differences, we recognize that other attributes differing among the predators likely contribute to the observed differences in prey behavioral repertoires. However, as our experimental design was meant to minimize the effects of many possible confounding factors (e.g. time of day, temperature, light, humidity, location, predators’ starvation levels and propensity to hunt) it seems likely that, in part, our results reflect hunting mode differences.

### Changes in activity level of *D*. *melanogaster* are associated with the presence of predators

In response to active hunters (those that constantly patrol for prey), we predicted that fruit flies would increase their overall activity levels (including flight) in order to maximize distance from the predator. However, to reduce the likelihood of an encounter with an ambush predator (i.e., a predator that only attacks when a prey organism wanders in to its strike zone), we predicted that *D*. *melanogaster* would respond by decreasing locomotory activities [10]. Our results were only partially in line with these predictions. While the actively hunting jumping spiders induce a clear increase in overall activity, we found the presence of juvenile mantids- our ambush predators- to have minimal influence on fruit fly activity levels (Fig 2, and Fig B in S2 File). It has been previously argued that ambush predators might be a predictable source of threat to prey organisms [9,10] as opposed to the diffuse and variable threat imposed by active hunters [10]. However, these predictions are based on studies from a grasshopper and its two natural predatory spider species that differ in hunting mode. In our study, whether or not *D*. *melanogaster* can perceive juvenile preying mantids as a threat is unknown. Therefore, the predator hunting-mode based predictions from the grasshopper study [10] may not be applicable to our system.

Many species of jumping spiders, including *S*. *scenicus*, are often seen in the natural habitat of *D*. *melanogaster* (personal observations of A.P., C.P. and I.D.), and are likely to be ecologically relevant predators of *Drosophila*. Juvenile mantids however, are rarely found in areas where fruit flies are abundant (personal observations of A.P. and I.D.), at least in eastern North America. Therefore, it is likely that fruit flies, having experienced a longer evolutionary history with jumping spiders, are better able to recognize any potential threat of these spiders. In addition, the disturbance created by a constantly patrolling zebra spider may be partly responsible for the increased activity levels seen in *D*. *melanogaster* (either due to actual mechanical disturbance or because flies are able to detect moving objects quicker than stationary ones). In this study, we are unable to tease apart the effects of evolutionary recognition versus constant mechanical disturbance on the differences in flies’ activity levels. Further experimentation with harmless but constantly moving heterospecifics (such as field crickets) or immobilized active hunters is needed to address these issues.

### Potentially relevant behaviors seen in the presence of predators

We also identified three behaviors of *D*. *melanogaster* that appeared to be most prominent during exposure to predators that may be relevant for future study.

#### Stopping

The behavior we called “stopping” (Table 1) was observed numerous times after a direct (but failed) attack by a spider (Video 1 in S2 File). Stopping or freezing behavior in response to predation threat has been documented in other species. Wong et al. (2005) showed that freezing is a common behavior in threatened crabs [49] and Brown et al. (2009) found that guppies often freeze in response to conspecific alarm cues [50]. While *D*. *melanogaster* will spend time without any ambulatory activity (walking, running), they are often observed to be active (generally grooming) during these periods. However, when fruit flies performed the stopping behavior, there was a complete lack of movement on the part of the fly, even when video was viewed at a few frames/second.

When a fruit fly was “stopped”, the spider had to search for the fly, irrespective of the physical proximity between the spider and the fly. In salticids, while the principal eyes have high spatial acuity, secondary eyes are primarily used to detect moving objects [28, 29]. Because salticids are unable to accommodate by changing the shape of their lens, they need to extensively sample their visual field to see details in object shape and form [28, 51, 52]. Scanning for prey by such sampling is likely a slow process unless guided by the motion sensing peripheral eyes, giving motionless prey the advantage of staying hidden (at least for a few seconds) while in plain sight of their salticid predator. Thus, *D*. *melanogaster* may be using the “stopping” behavior as a potential mechanism to reduce the likelihood of detection by the spider.

A superficially similar behavior called “freezing” has been previously described in *D*. *melanogaster* by Card and Dickinson (2008) (reviewed in [53]). However, the freezing behavior was an instantaneous behavior (akin to our “pause” behavior), observed only in the legs, and performed just prior to a jump. In our study, we did not observe many transitions from stops (or pauses) to jumping, although we observed a significant over-representation of transition to the stopping behavior after a jump or flight. Therefore, it is unlikely that the freezing behavior described by Card and Dickinson (2008) and stopping behavior described in this study are the same.

#### Abdominal lifting

In the presence of both predators, male and female *D*. *melanogaster* substantially increased the frequency at which they performed abdominal lifts. Although many studies have described abdominal bobbing movements in ants and ant-mimicking Myrmarachne salticid spiders in the context of aggression and defense [54–57], to our knowledge, abdominal lifting, in the context of interaction with a potential predator, has not been described in the *D*. *melanogaster* literature prior to this study. Spieth (1952), described a variety of abdominal movements (primarily curling and extension) in different *Drosophila* species [58]. In each case, this was performed in the context of active courting between a male and a female or attempted copulation with a conspecific. Based on the descriptions within Spieth (1952), it seems likely that in addition to context (hetero-specific predator versus conspecific mate) the physical manifestation of the behaviors differ. In a study about courtship behaviors in female *D*. *melanogaster*, Lasbleiz et al. (2006) described two behaviors: abdominal drumming and abdominal extension [40]. Abdominal drumming (described as “quickly repeated vertical movements of the abdomen which is tapped on the substrate”) was only seen in males during courtship display, and abdominal extensions (described as “abdomen raised by 15–30 degrees” seen in both males and females) were also closely associated with courtship. The adaptive significance of abdominal bobbing and abdominal lifting in the context of courtship is yet unknown.

In our study, abdominal lifting was often directed at a predator or followed a failed predatory encounter, and there were no potential mates for the flies in the arena. If abdominal lifting is indeed anti-predatory, it could function in several possible ways. Here we elaborate on three (out of several) hypotheses: first, abdominal lifting may be a signal of prey condition directed at the predator as a form of pursuit deterrence, comparable to stotting in the Thomson’s gazelle [59]. Second, because *D*. *melanogaster* are often surrounded by conspecifics, flies may use abdominal lifting as a signal to warn conspecifics of the presence of a potential threat (similar in function to fin flicking in tetras, [60]). Third, abdominal lifting may be an indication of some sort of physiological priming of the fly in preparation for a fight-or-flight response, or simply a response to “fear” with respect to the predator. Determining whether it is a specific anti-predator behavior, as well as the details of its function need to be a focus of future work.

#### Retreat

In response to the juvenile praying mantids, half of the fruit flies (15/30) performed a reverse locomotory behavior which we have called “retreat”, where the flies walked in reverse, away from the predator (Video 3 in S2 File). This was often (but not always) interspersed with the abdominal lifting behavior. Phenomenologically, this behavior appears to be similar to that described in [61]. Bidaye et al. (2014) identified neurons that upon activation changed walking direction in *D*. *melanogaster*. Bidaye et al.’s reverse walking behavior appears to be a smooth and continuous behavior, whereas “retreat” was often discontinuous and accompanied by abdominal lifting. If the two “retreat” behaviors are related, the observed disassociation between retreat and abdominal lifting as well as its continuous nature (in [61]) may be a function of how the neurons were perturbed. Further investigation is necessary to uncover the relatedness between the two retreat behaviors, as well as any potential role in response to predators.

### Transitions among behaviors

We also investigated how the presence of the different predators may influence non-random associations among behaviors. We observed that in the presence of both predators there was an increase in the number of behavioral transitions that deviated from expectations under independence (from 12 to 23 with the mantid, and 8 to 13 with the spider). Despite this, the log-linear model (analyzing the whole transition frequency matrix) did not support the influence of predator state on the frequencies of transitions. This may be partly due to the relatively modest sample sizes (in terms of both individuals and transitions among behaviors). Further work is necessary to validate and extend this sequential analysis.

Because we tracked a large number of fruit fly behaviors, we were unable to simultaneously record predator behaviors. We could take the predator location (“predator close” vs. “predator far”–see methods for more details) into account. However, our experimental design could not disentangle predator distance from the predator’s intent to strike. In some cases, when the predator was within striking distance, it faced away from the fly. Additionally, very few individuals were captured in the mantid treatment, while half the individuals were captured in the spider treatment. We were, thus, unable to identify *D*. *melanogaster* behavioral sequences that were associated with a higher probability of escape. To estimate the probability of capture, future experiments should focus on a small subset of the described *D*. *melanogaster* behaviors, along with some predator behaviors (e.g. “track”, “crouch”, “orient” and “attack”).

### Inter-individual variation in *D*. *melanogaster* behaviors

Finally, we show that there is considerable inter-individual variation in *D*. *melanogaster’s* baseline activity levels, as well as, in their response to both predators. This among-individual variation was greater in response to juvenile mantids than to jumping spiders. Several factors, including inter-individual variability in predator activity levels and presence of different fruit fly “personalities” (i.e. behavioral syndromes), may contribute to the observed variability in *D*. *melanogaster’s* behavioral responses. Further investigation is needed to understand the relationship between prey behavioral variability and hunting mode.

We also saw a negative correlation in duration of time spent on grooming and locomotory behaviors before and after the addition of a jumping spider but not a praying mantid. In other words, fruit flies that spent more time grooming (and less time walking) in the absence of a jumping spider, tended to reduce their grooming (and increase their walking) to a greater extent in the presence of a jumping spider. Given the limitations of our experimental design, we are unable to attribute this negative behavioral correlation to hunting mode. We therefore, limit our discussion to a description of the variability. A larger sample size, additional predator pairs and controls are necessary to understand the role of predator hunting mode in shaping these behavioral correlations.

### Caveats of this study

While we show that there are some predator hunting-mode specific behavioral differences in *D*. *melanogaster’s* anti-predator response, we reiterate five important caveats.

First, although the primary distinction between the zebra jumping spider and juvenile Chinese praying mantids as predators is their hunting-mode, other factors between these species (for example, size, color, odor) may influence differences in fruit fly behaviors. Replicating the observations with other predator pairs that differ in hunting-mode is necessary to confirm hunting-mode’s influence on anti-predatory repertoires.

Second, some changes in *D*. *melanogaster* behavior seen as a response to the predator, may be generic responses to any heterospecific (predatory or non-predatory) individual. For example, fruit flies may respond to the presence of an active but harmless field cricket by increasing their overall locomotion for a short period of time. Further, the introduction of a conspecific (instead of a predator) may also lead to similar changes in activity levels. Additional controls with “harmless-active” and “harmless-stationary” heterospecifics and conspecifics must be conducted to identify *D*. *melanogaster* behaviors that are exclusively anti-predatory.

Third, our assay chambers are an artificial environment and do not resemble the conditions under which *D*. *melanogaster* face predators in the wild. Due to the nature of our assay chamber, *D*. *melanogaster* were unable to employ some behavioral strategies that may reduce encounters with predators (e.g., utilizing a refuge). Therefore we were only able to describe the capture-deterrence repertoire of *D*. *melanogaster* behaviors.

Fourth, although we found *D*. *melanogaster’s* behavior to be temporally homogenous in the absence of a predator, the behavioral repertoire may change slightly over (even relatively short) periods of time. Given the differences in observation times for “predator present” (10 minutes) vs. “predator absent” (5 minutes) treatments, we account for a possible temporal effect on *D*. *melanogaster’s* behavioral repertoire by adding “duration of time spent in the assay chamber” as a predictor variable in our statistical model. We performed an experiment in the absence of predators but with a manual disturbance to simulate predator entrance into the arena, and observed no temporal change (Table M in S2 File). However, confounding temporal effects on *D*. *melanogaster* behaviors in the “predator present” treatment cannot be completely ruled out.

Fifth, the wild caught population we used in this study was maintained in the lab for 2 years before our behavioral assays were performed. Domesticated populations are more susceptible to predation than their wild counterparts [62–65]. Lack of predation experience during development along with prolonged relaxed selection results in the loss of traits that were once advantageous in the wild. Therefore, we can expect some anti-predatory behaviors to be lost (or performed at lower frequencies) in our domesticated population, while other less-relevant (and potentially unfit) behaviors to become more prevalent.

We believe that our study is a necessary first step to describing and documenting the complete anti-predatory behavioral repertoire of *D*. *melanogaster*, and we foresee future work to be conducted in a modified chamber, under more “natural” conditions, and with a freshly captured population of *D*. *melanogaster*. Doing so will allow us to take this premier model genetic system and make it into an ecological model as well.

## Supporting information

S1 File Ethograms of 60 individual fruit flies in the absence and presence of either a zebra jumping spider or a juvenile Chinese praying mantid. (PDF)

S2 File Supplemental methods, figures, tables and links to videos. (DOCX)
