# Supplementary material for: The behavioral repertoire of Drosophila melanogaster in the presence of two predator species that differ in hunting mode
Source: PLoS One. 2019 May 31;14(5):e0216860. doi: 10.1371/journal.pone.0216860 (PMC6544228; doi:10.1371/journal.pone.0216860)
Supplement: S1 File — (PDF) [file pone.0216860.s001.pdf]

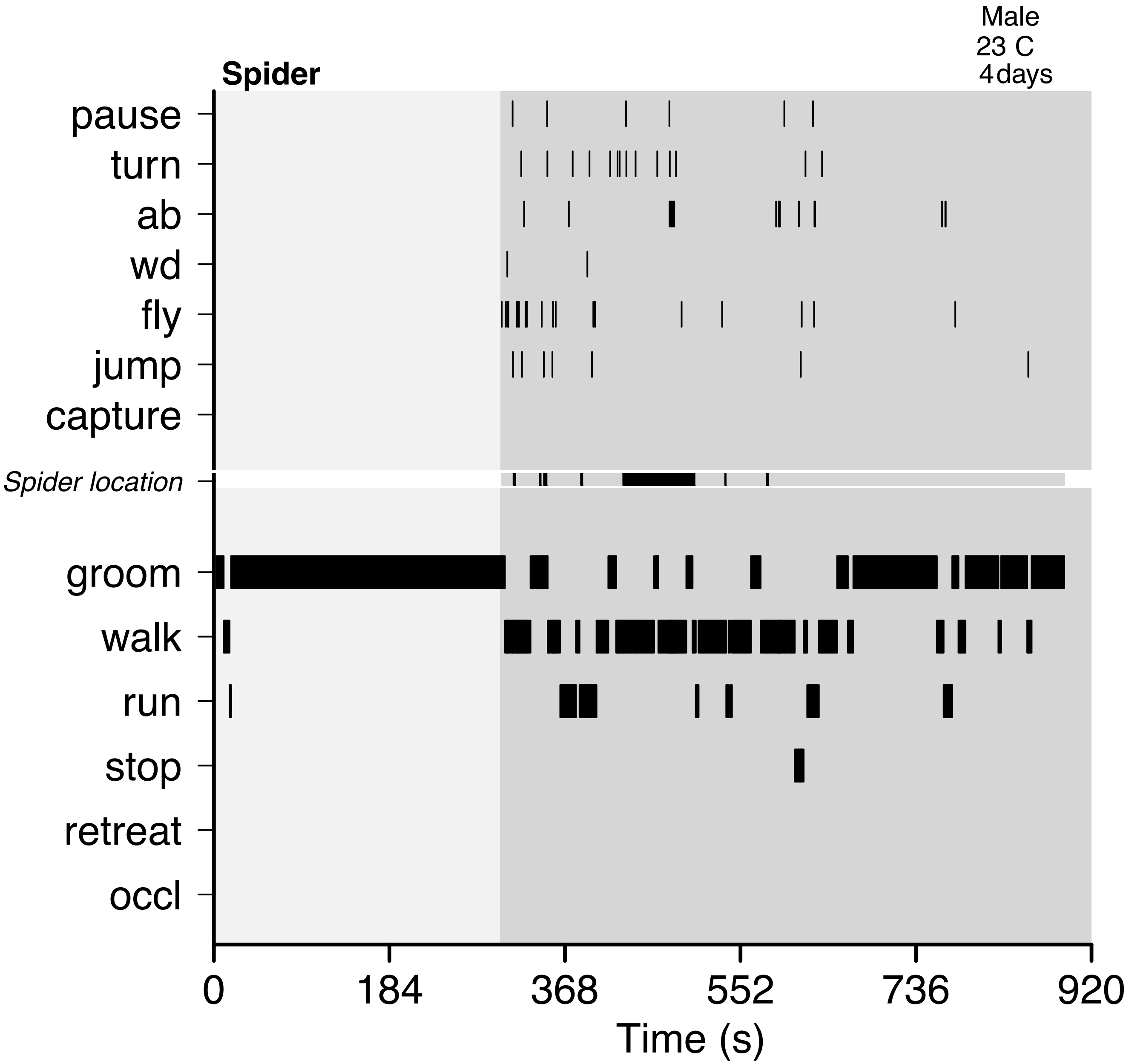

Male
23 C
4 days
Spider
pause
turn
ab
wd
fly
jump
capture
Spider location
groom
walk
run
stop
retreat
occl
0
184
368
552
736
920
Time (s)

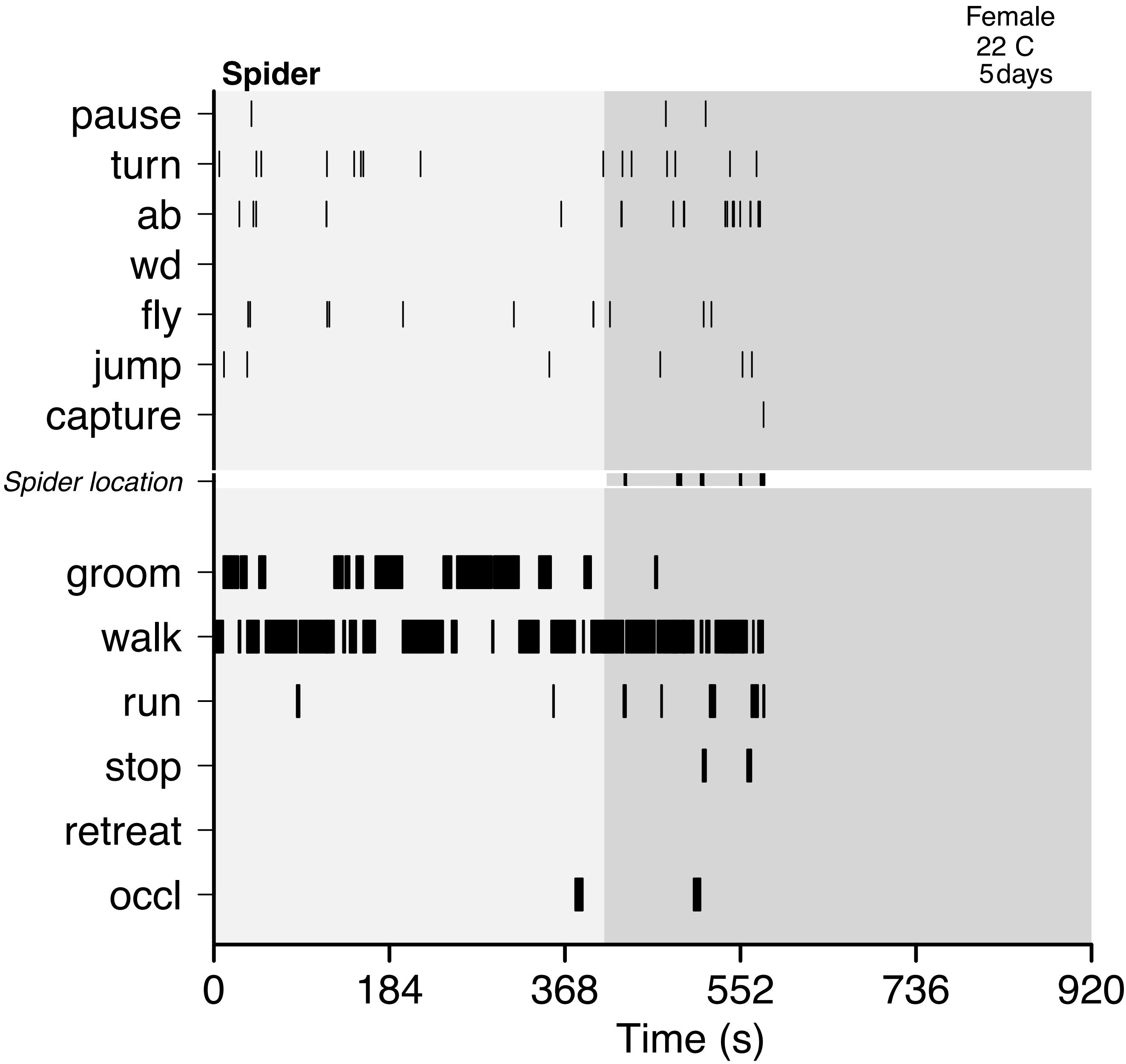
Female
22 C
5 days
Spider
pause
turn
ab
wd
fly
jump
capture
Spider location
groom
walk
run
stop
retreat
occl
0
184
368
552
736
920
Time (s)

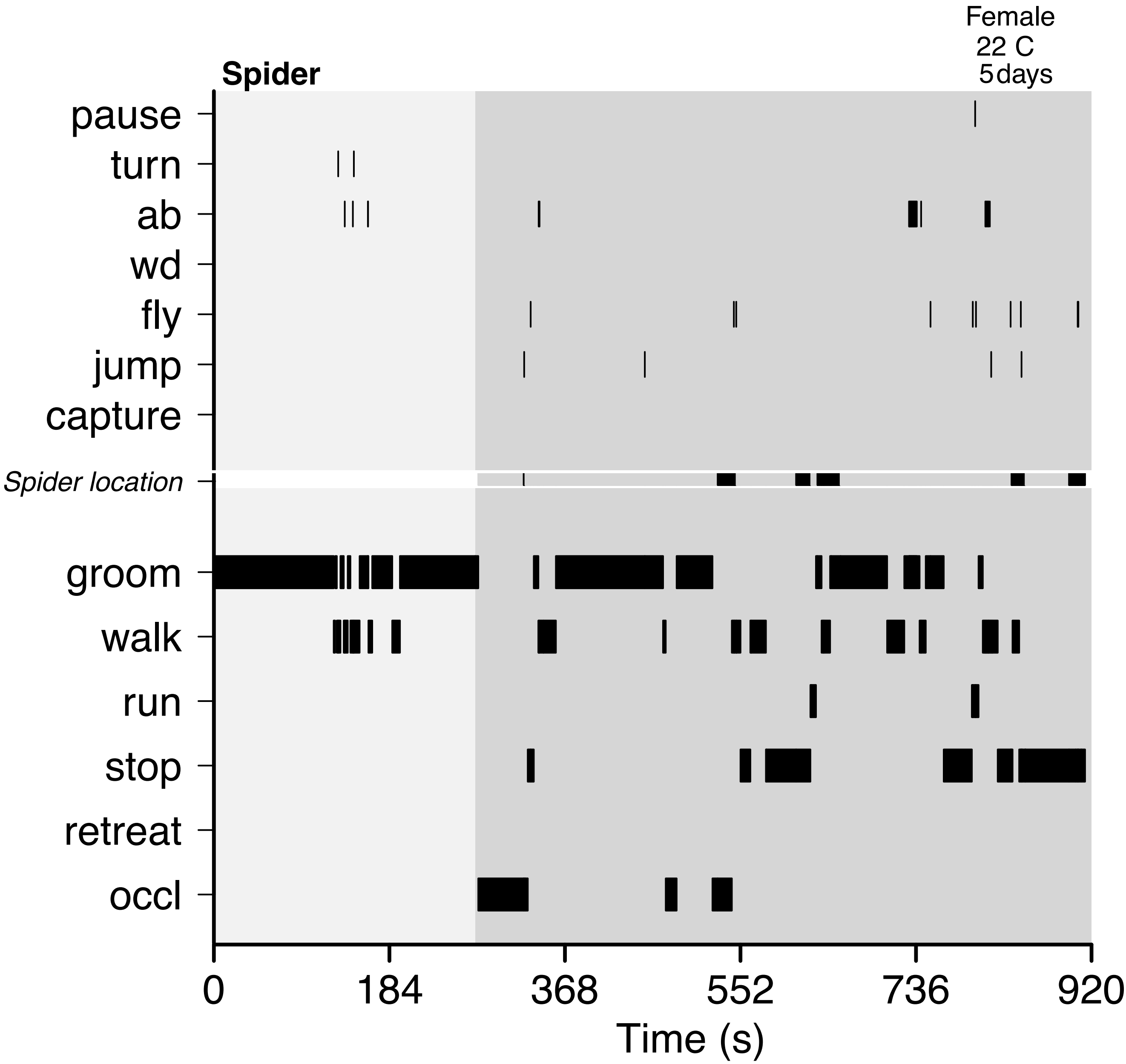

Female
22 C
5 days
Spider
pause
turn
ab
wd
fly
jump
capture
Spider location
groom
walk
run
stop
retreat
occl
0
184
368
552
736
920
Time (s)

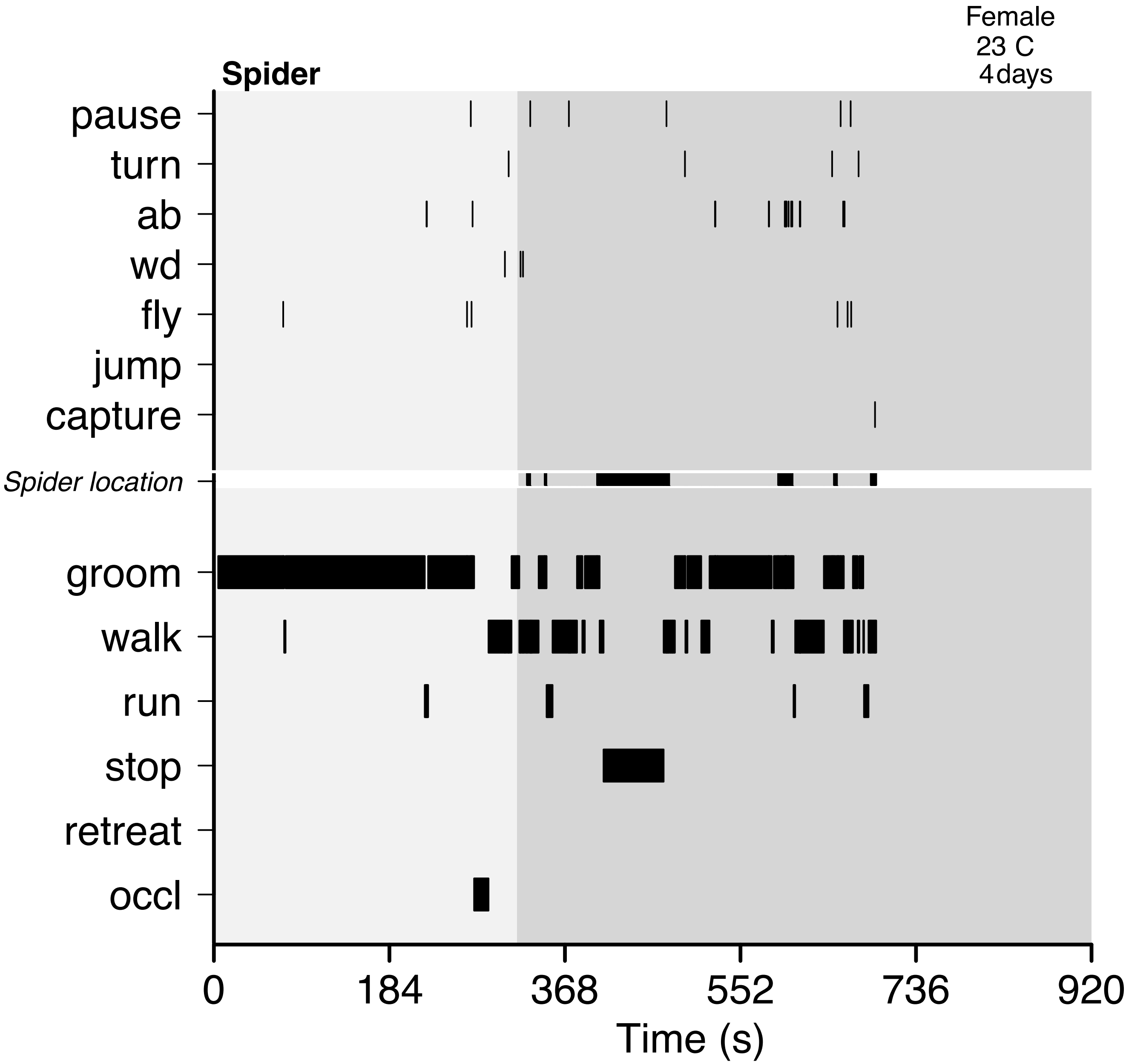

Female
23 C
4 days
Spider
pause
turn
ab
wd
fly
jump
capture
Spider location
groom
walk
run
stop
retreat
occl
0
184
368
552
736
920
Time (s)

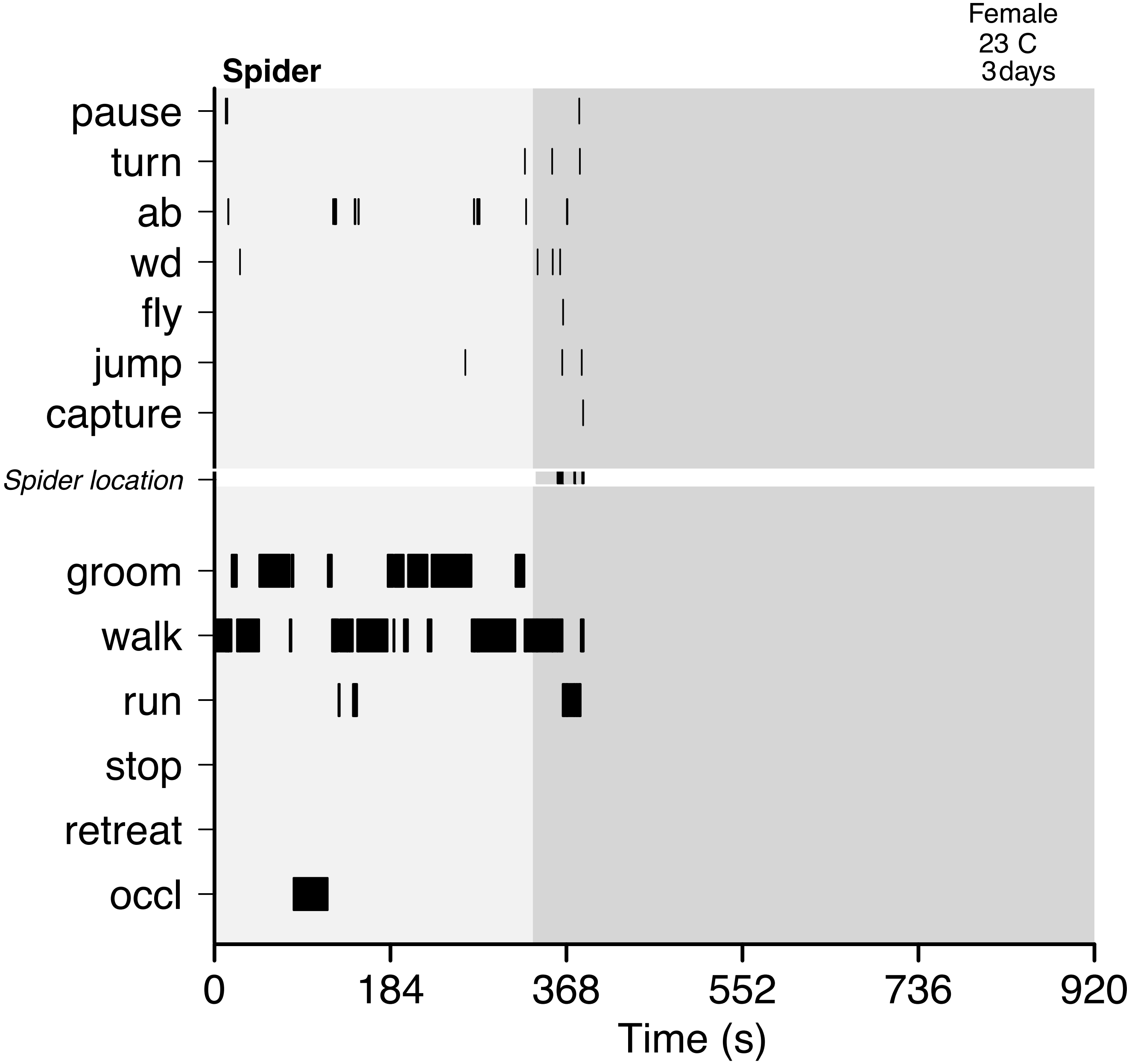

Female
23 C
3days
Spider
pause
turn
ab
wd
fly
jump
capture
Spider location
groom
walk
run
stop
retreat
occl
0
184
368
552
736
920
Time (s)

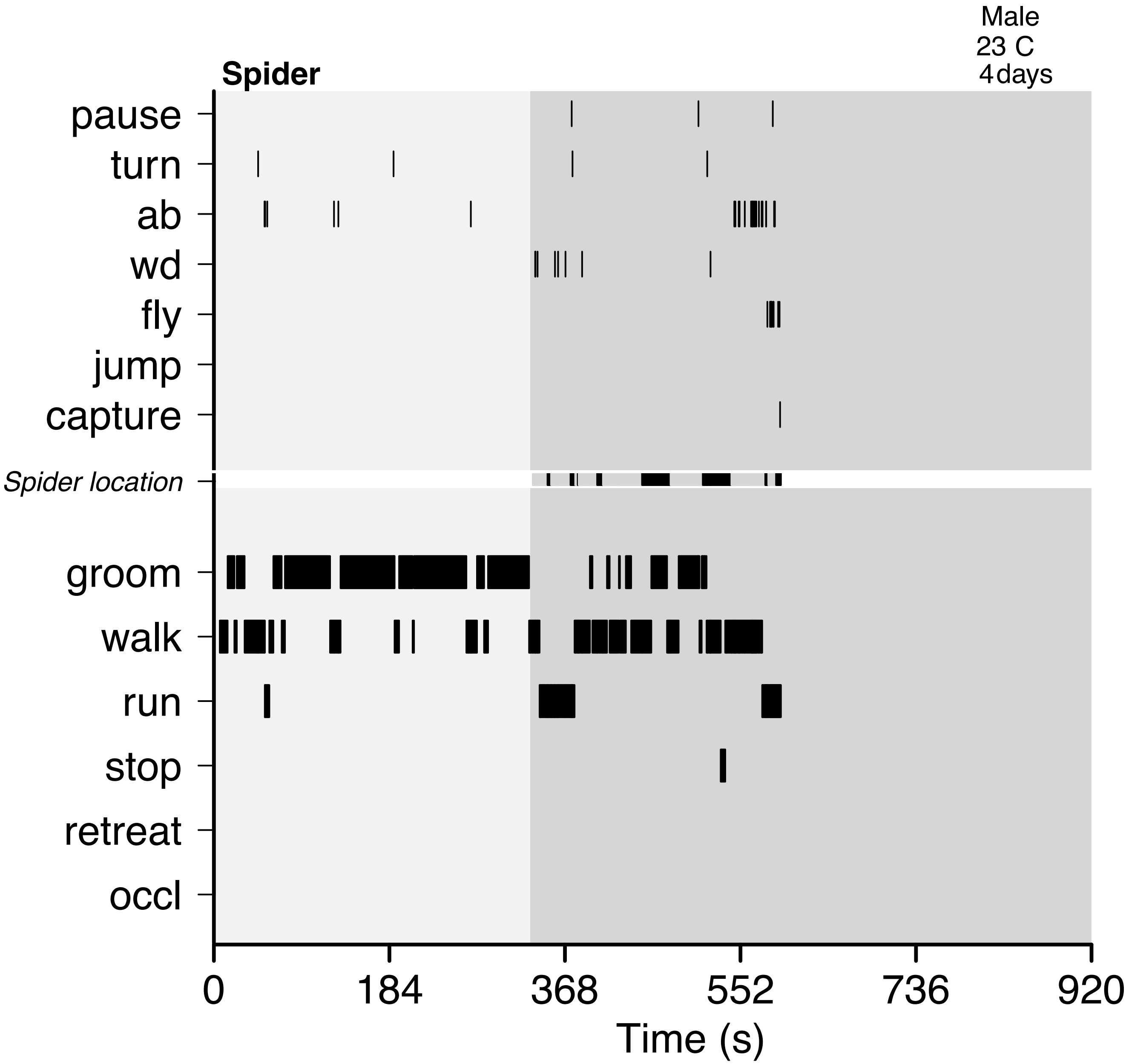

Male
23 C
4 days
Spider
pause
turn
ab
wd
fly
jump
capture
Spider location
groom
walk
run
stop
retreat
occl
0
184
368
552
736
920
Time (s)

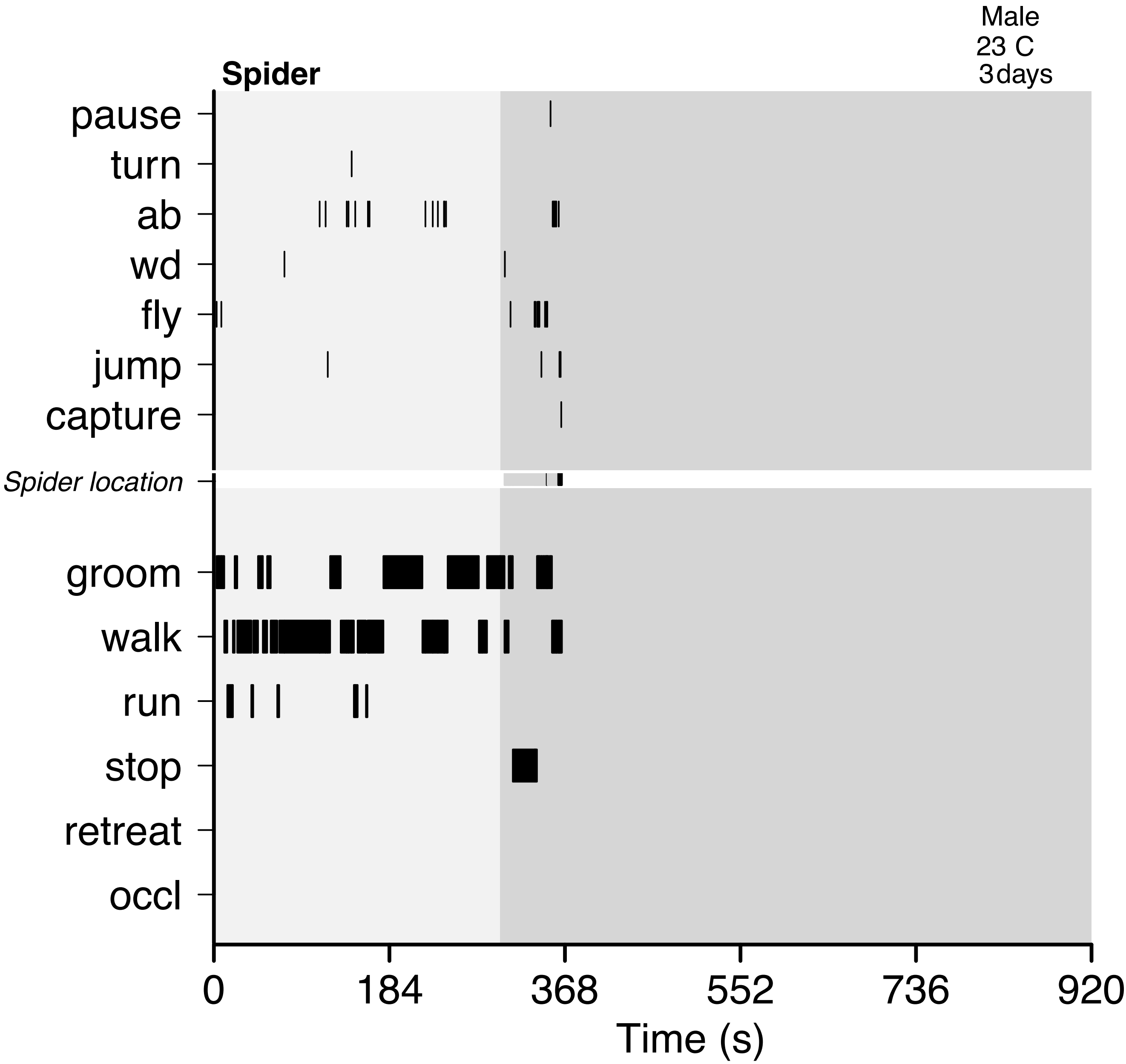
Male
23 C
3days
Spider
pause
turn
ab
wd
fly
jump
capture
Spider location
groom
walk
run
stop
retreat
occl
0
184
368
552
736
920
Time (s)

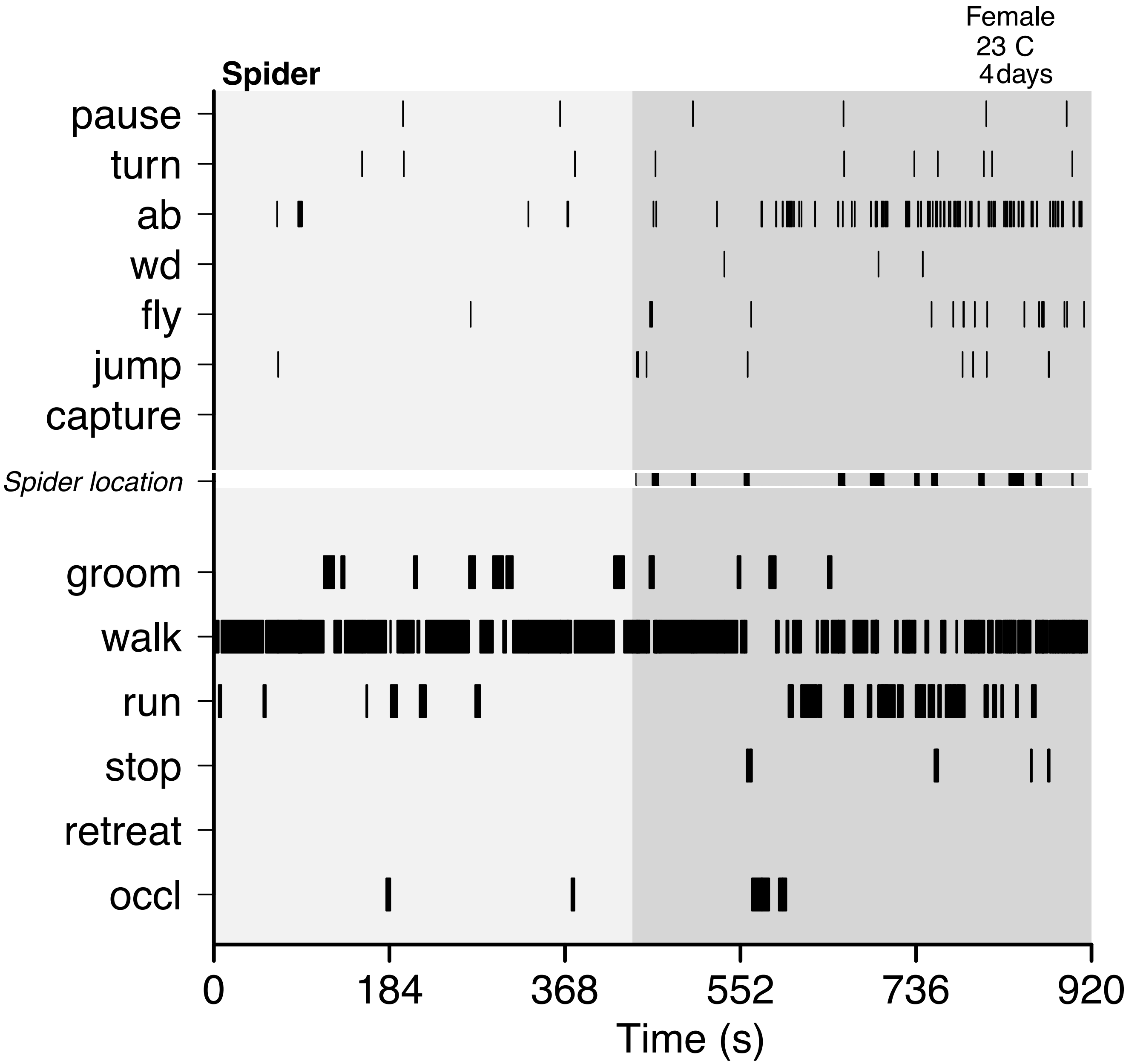

Female
23 C
4 days
Spider
pause
turn
ab
wd
fly
jump
capture
Spider location
groom
walk
run
stop
retreat
occl
0
184
368
552
736
920
Time (s)

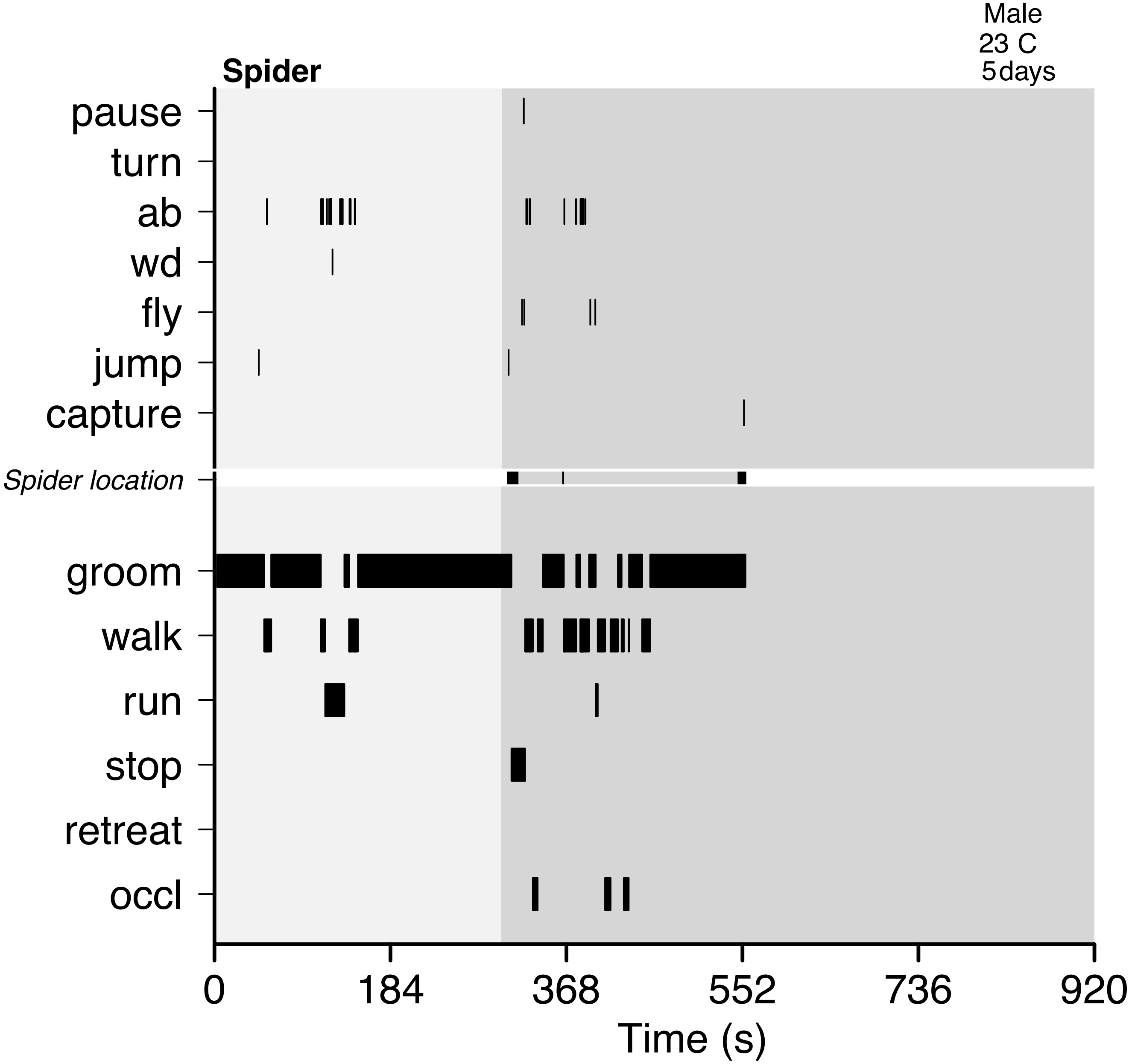
Male
23 C
5 days
Spider
pause
turn
ab
wd
fly
jump
capture
Spider location
groom
walk
run
stop
retreat
occl
0
184
368
552
736
920
Time (s)

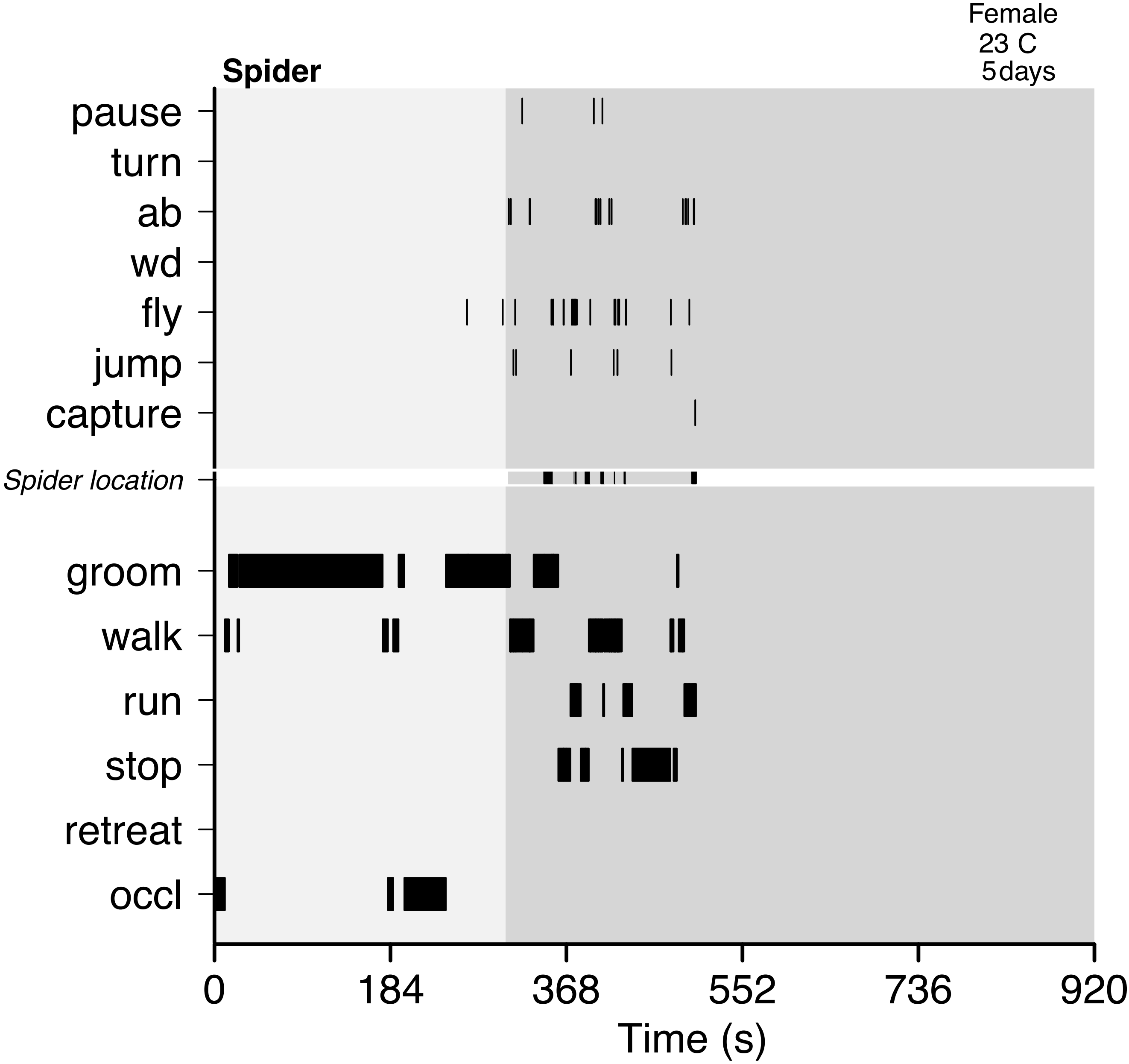
Female
23 C
5 days
Spider
pause
turn
ab
wd
fly
jump
capture
Spider location
groom
walk
run
stop
retreat
occl
0
184
368
552
736
920
Time (s)

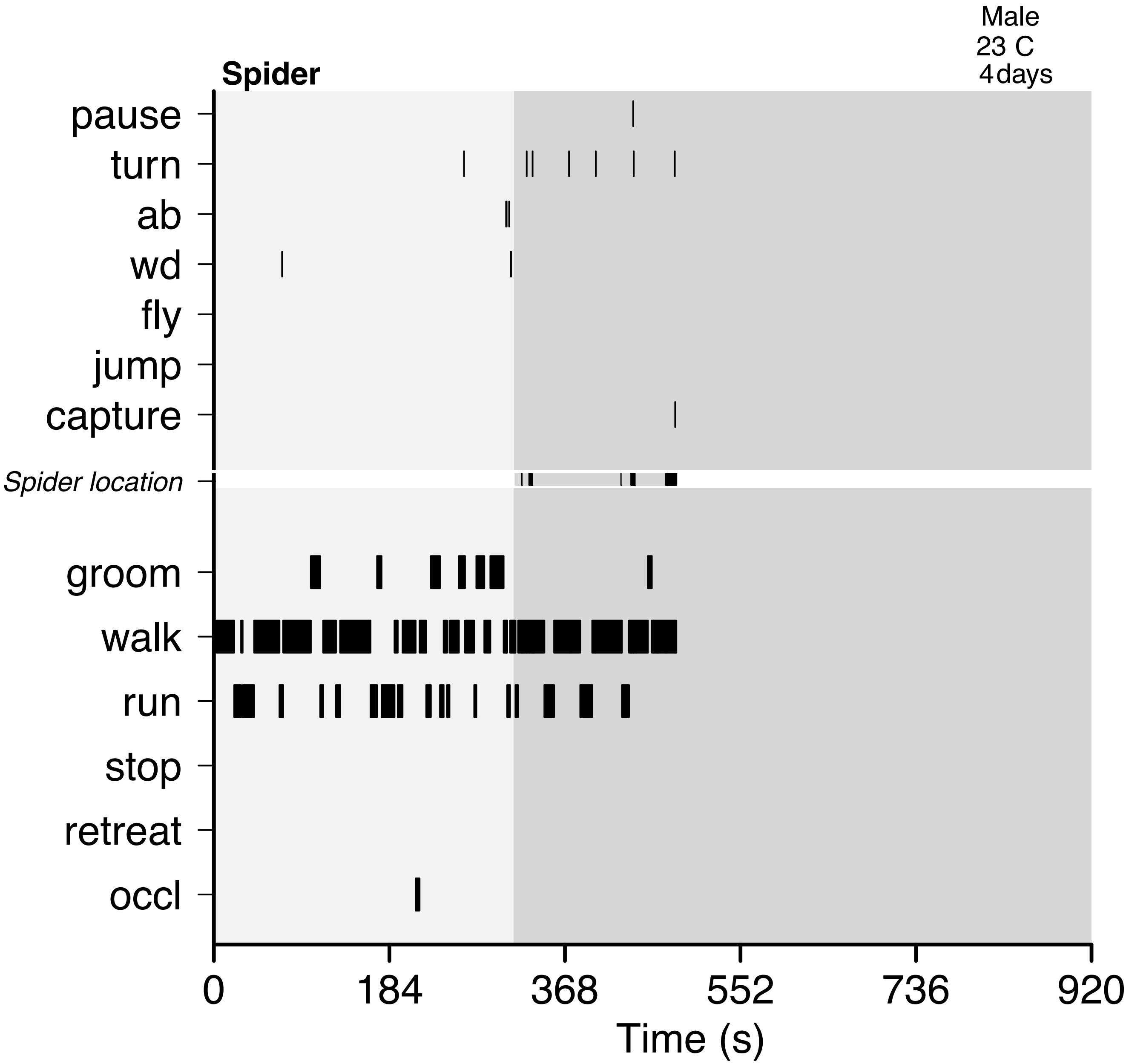
Male
23 C
4days
Spider
pause
turn
ab
wd
fly
jump
capture
Spider location
groom
walk
run
stop
retreat
occl
0
184
368
552
736
920
Time (s)

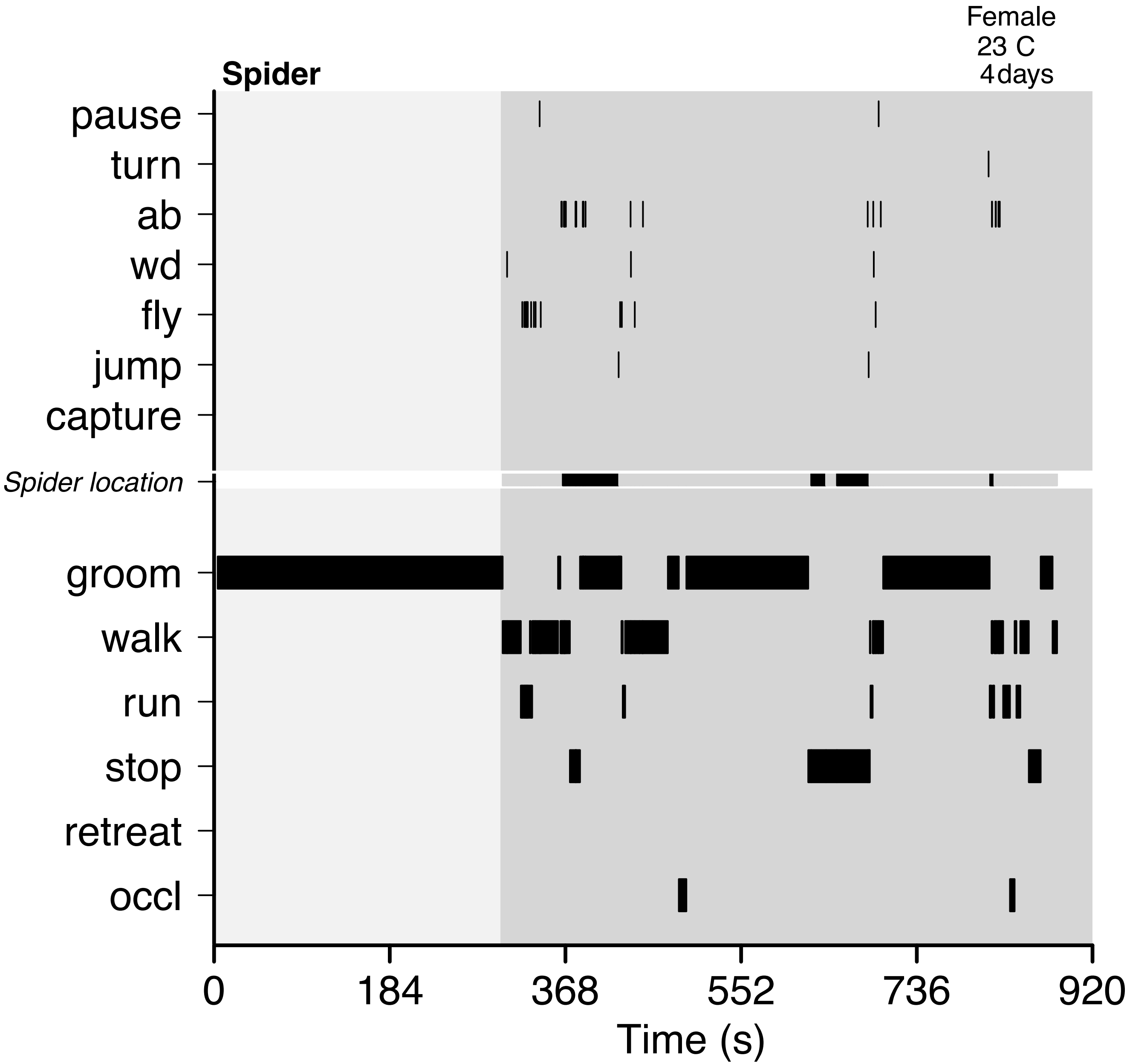

Female
23 C
4 days
Spider
pause
turn
ab
wd
fly
jump
capture
Spider location
groom
walk
run
stop
retreat
occl
0
184
368
552
736
920
Time (s)

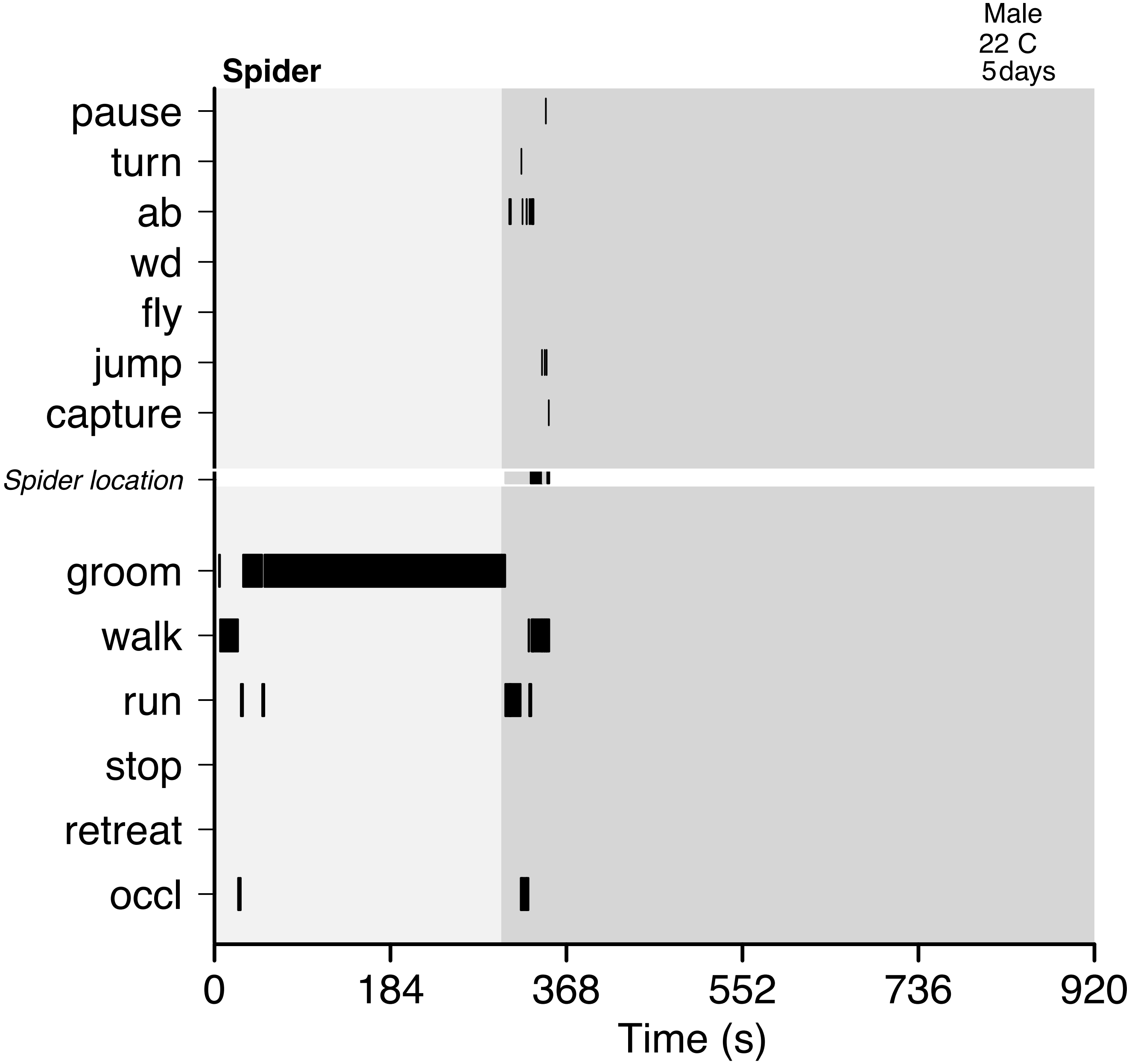

Male
22 C
5 days
Spider
pause
turn
ab
wd
fly
jump
capture
Spider location
groom
walk
run
stop
retreat
occl
0
184
368
552
736
920
Time (s)

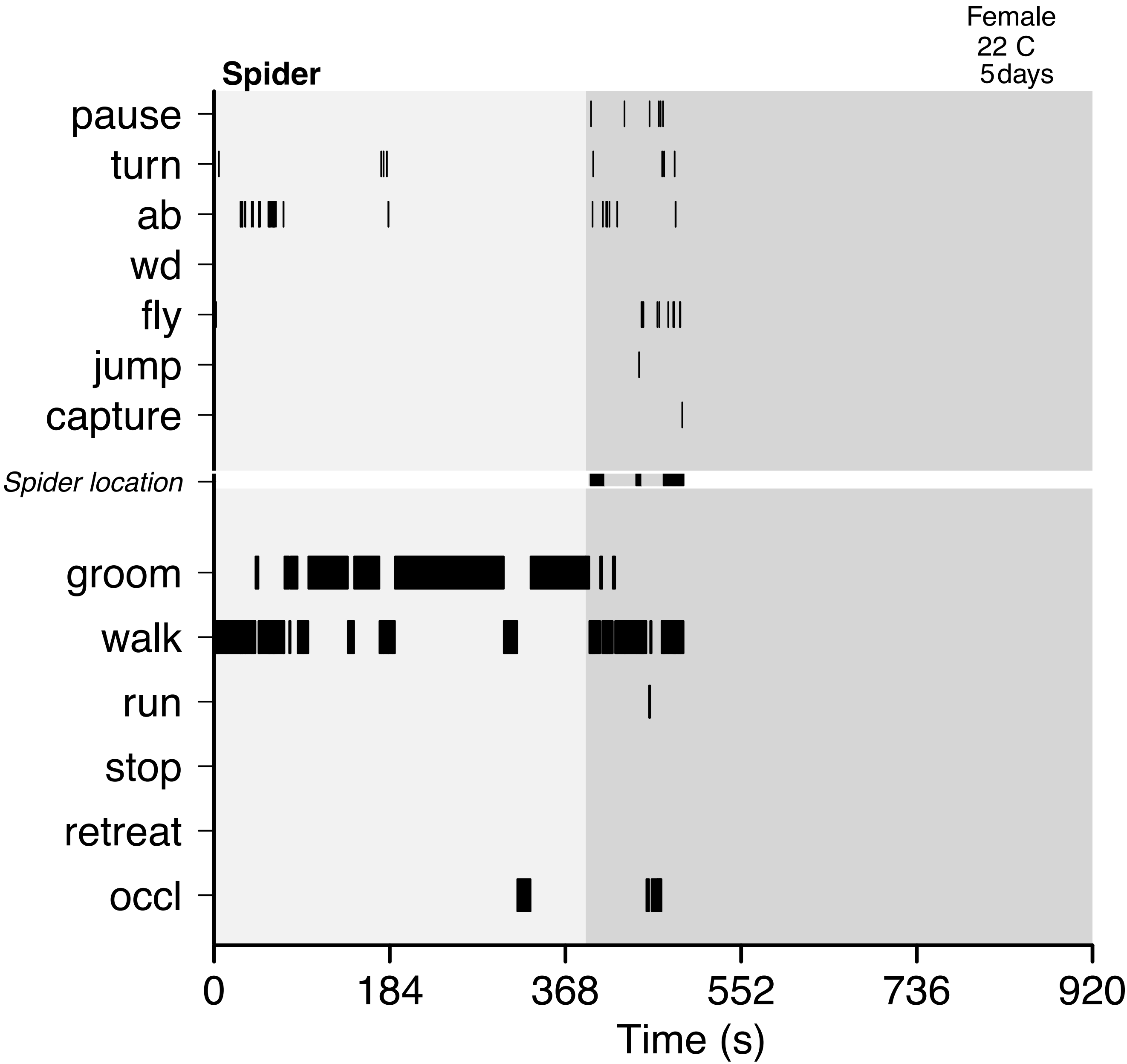
Female
22 C
5 days
Spider
pause
turn
ab
wd
fly
jump
capture
Spider location
groom
walk
run
stop
retreat
occl
0
184
368
552
736
920
Time (s)

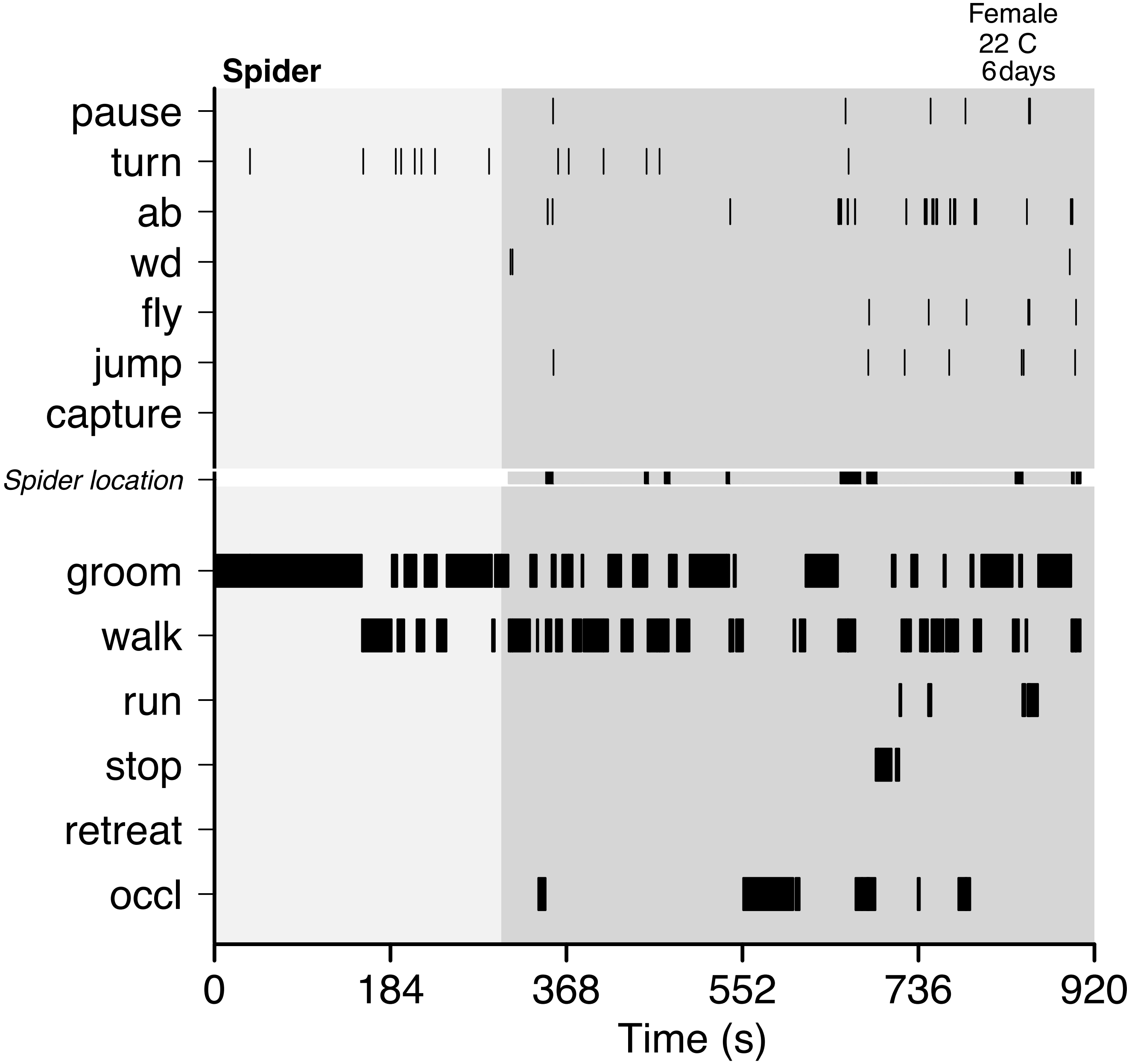

Female
22 C
6 days
Spider
pause
turn
ab
wd
fly
jump
capture
Spider location
groom
walk
run
stop
retreat
occl
0
184
368
552
736
920
Time (s)

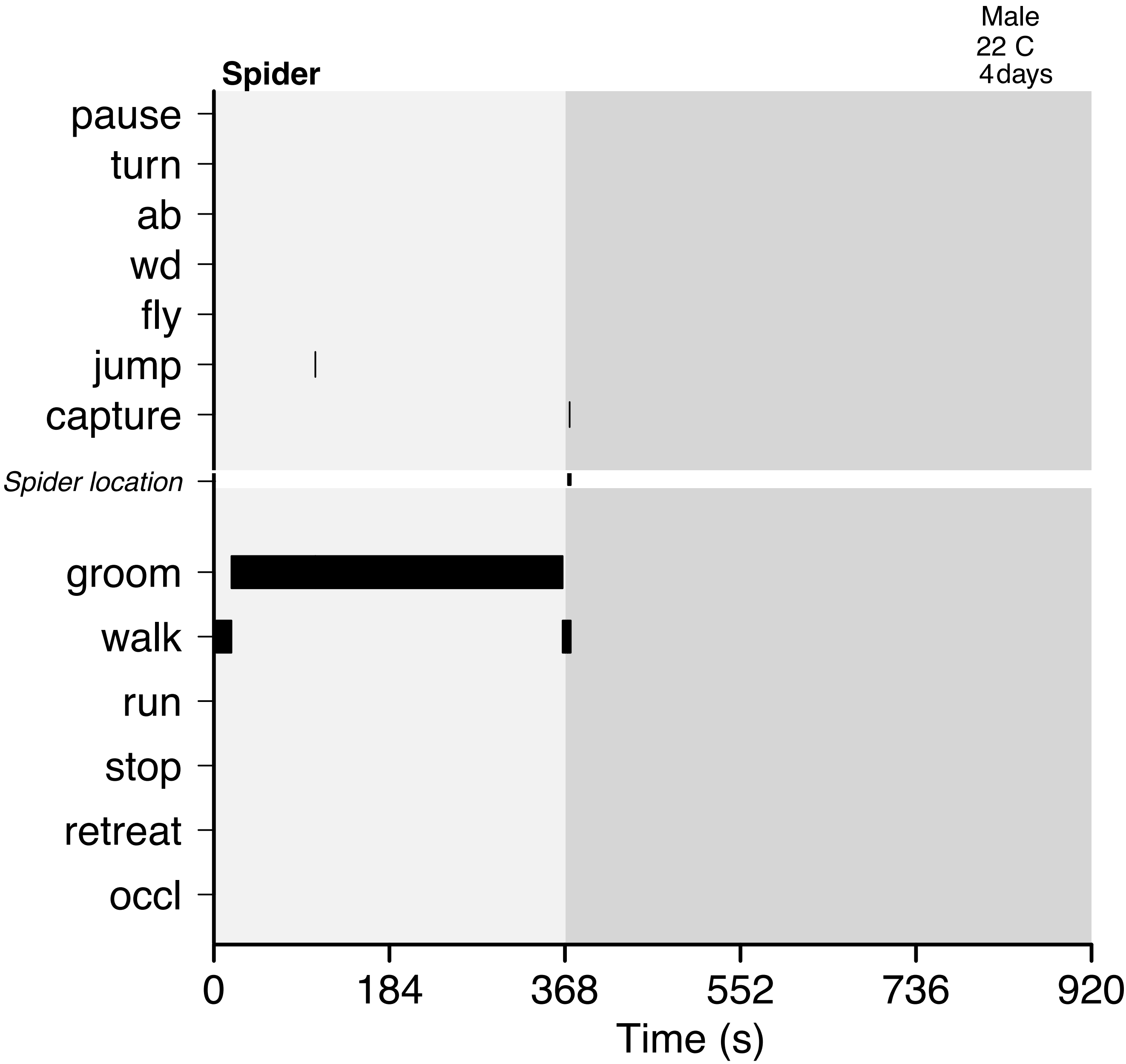
Male
22 C
4 days
Spider
pause
turn
ab
wd
fly
jump
capture
Spider location
groom
walk
run
stop
retreat
occl
0
184
368
552
736
920
Time (s)

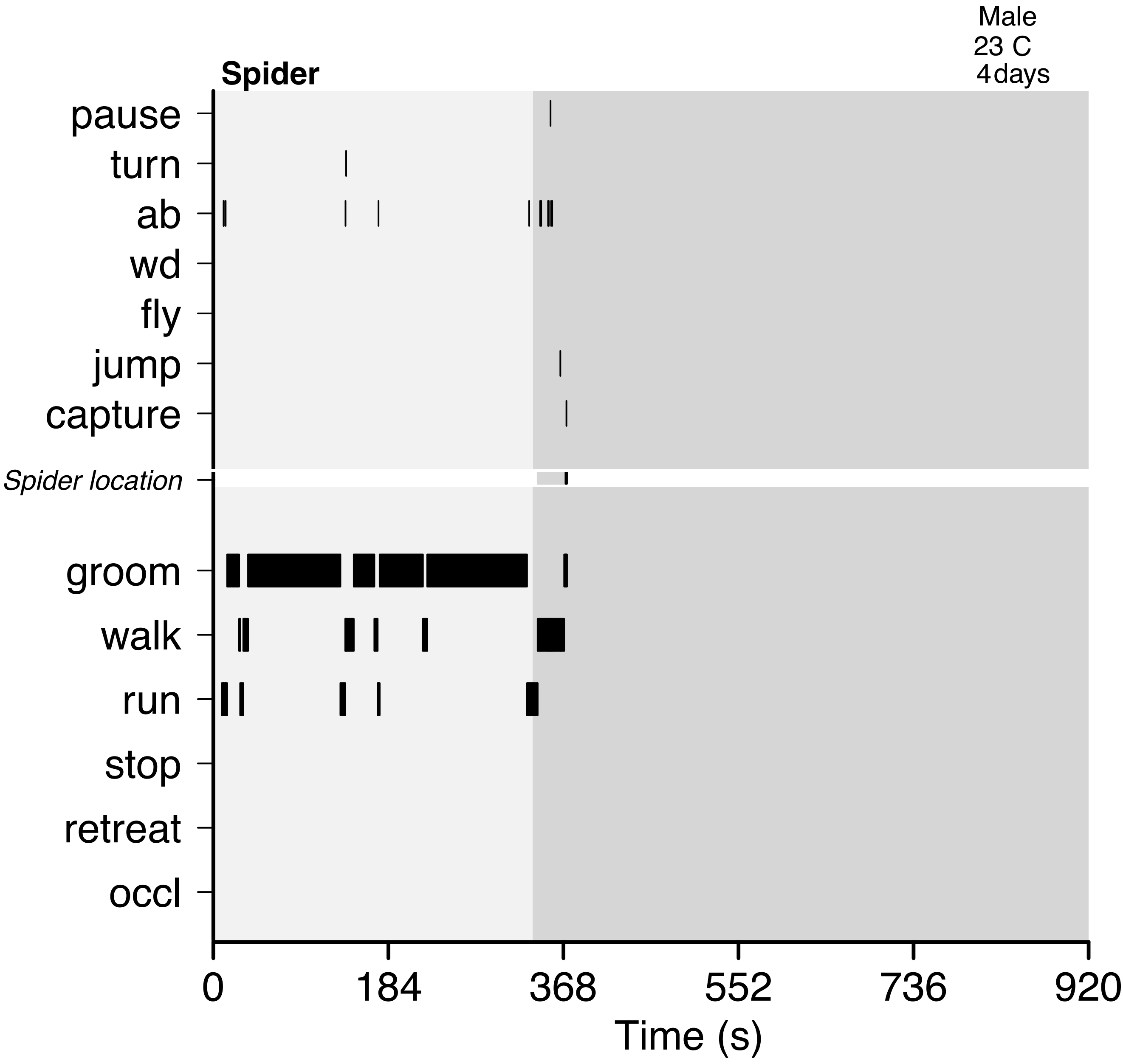
Male
23 C
4 days
Spider
pause
turn
ab
wd
fly
jump
capture
Spider location
groom
walk
run
stop
retreat
occl
0
184
368
552
736
920
Time (s)

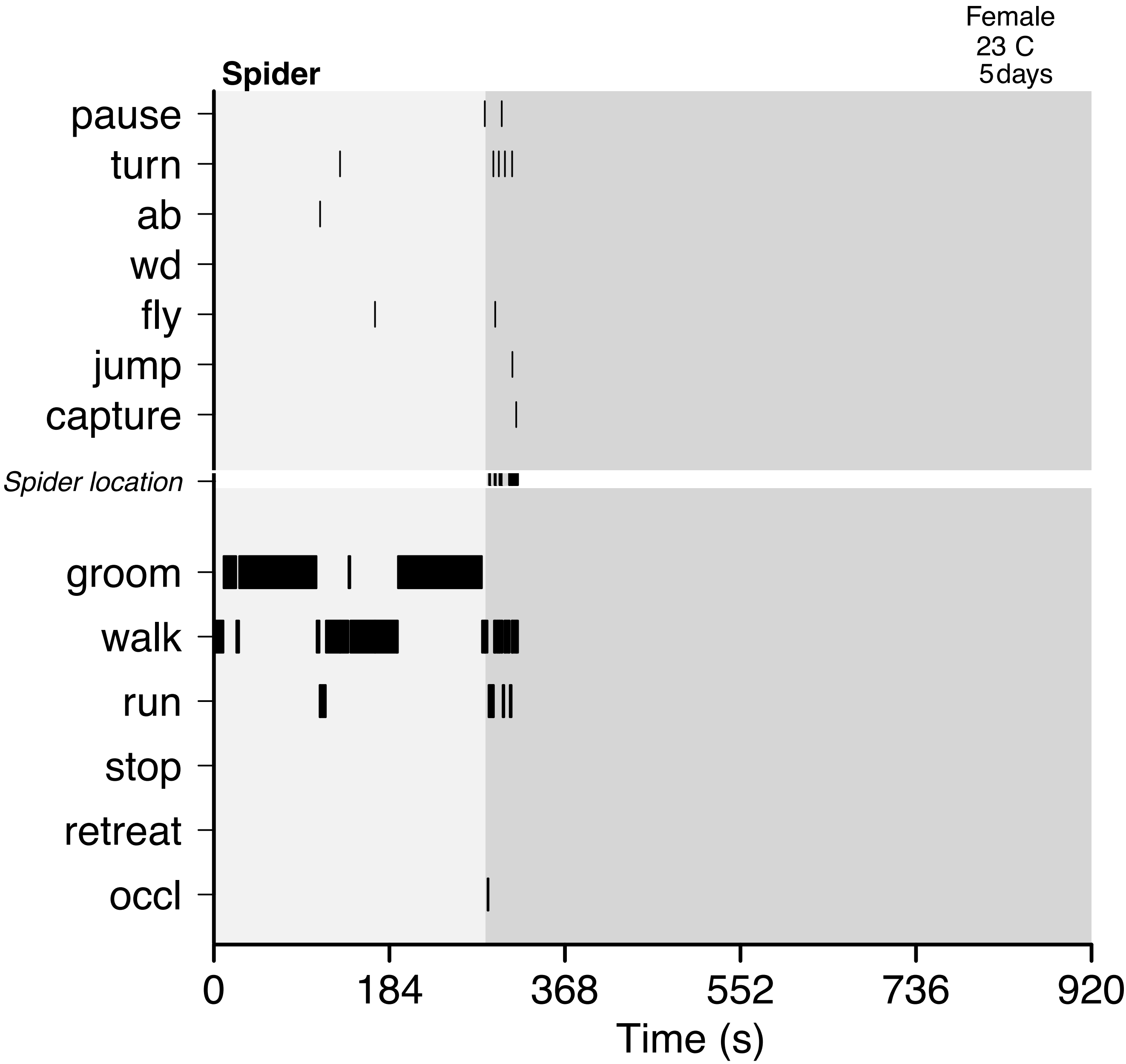

Female
23 C
5 days
Spider
pause
turn
ab
wd
fly
jump
capture
Spider location
groom
walk
run
stop
retreat
occl
0
184
368
552
736
920
Time (s)

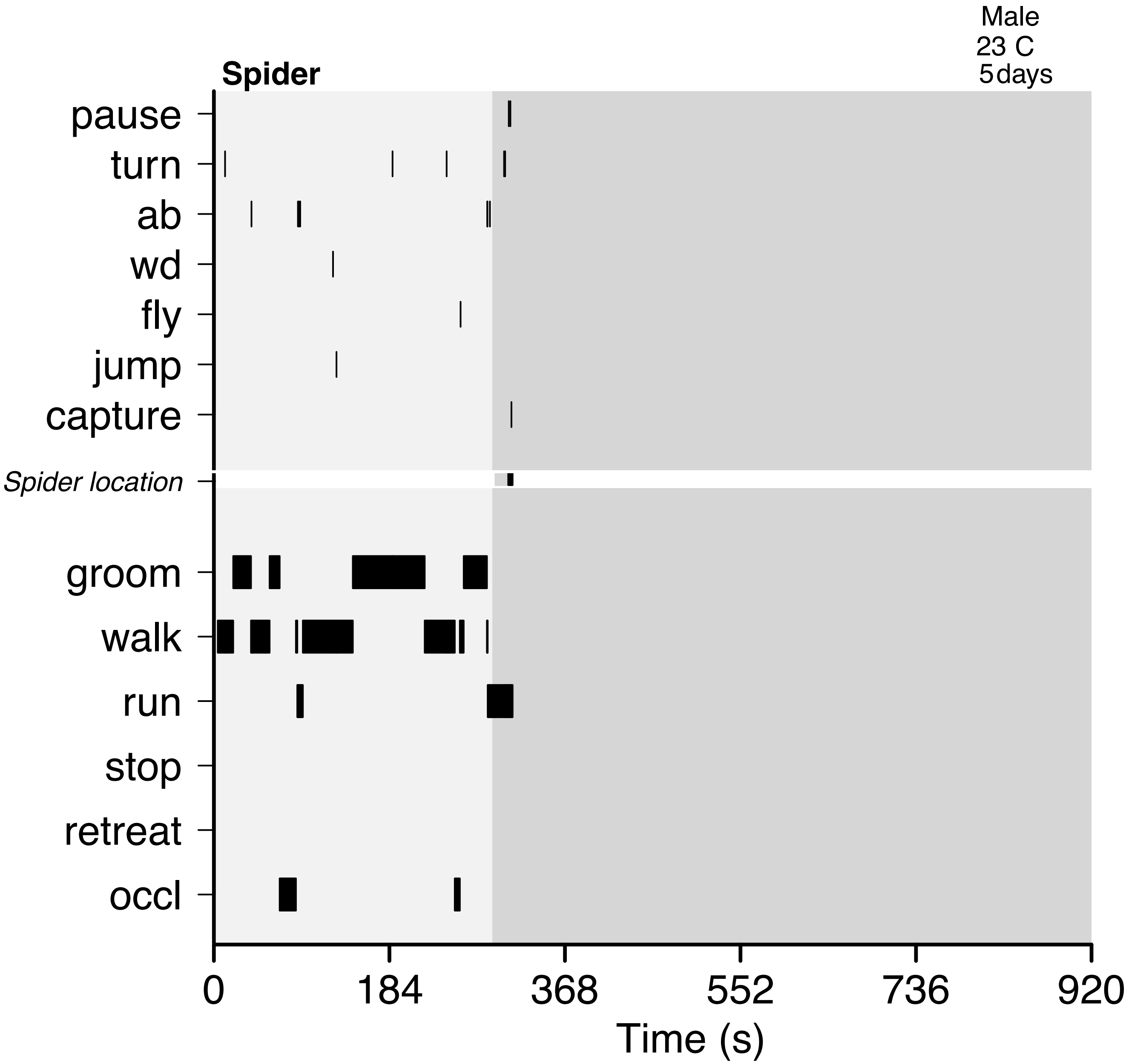
Male
23 C
5 days
Spider
pause
turn
ab
wd
fly
jump
capture
Spider location
groom
walk
run
stop
retreat
occl
0
184
368
552
736
920
Time (s)

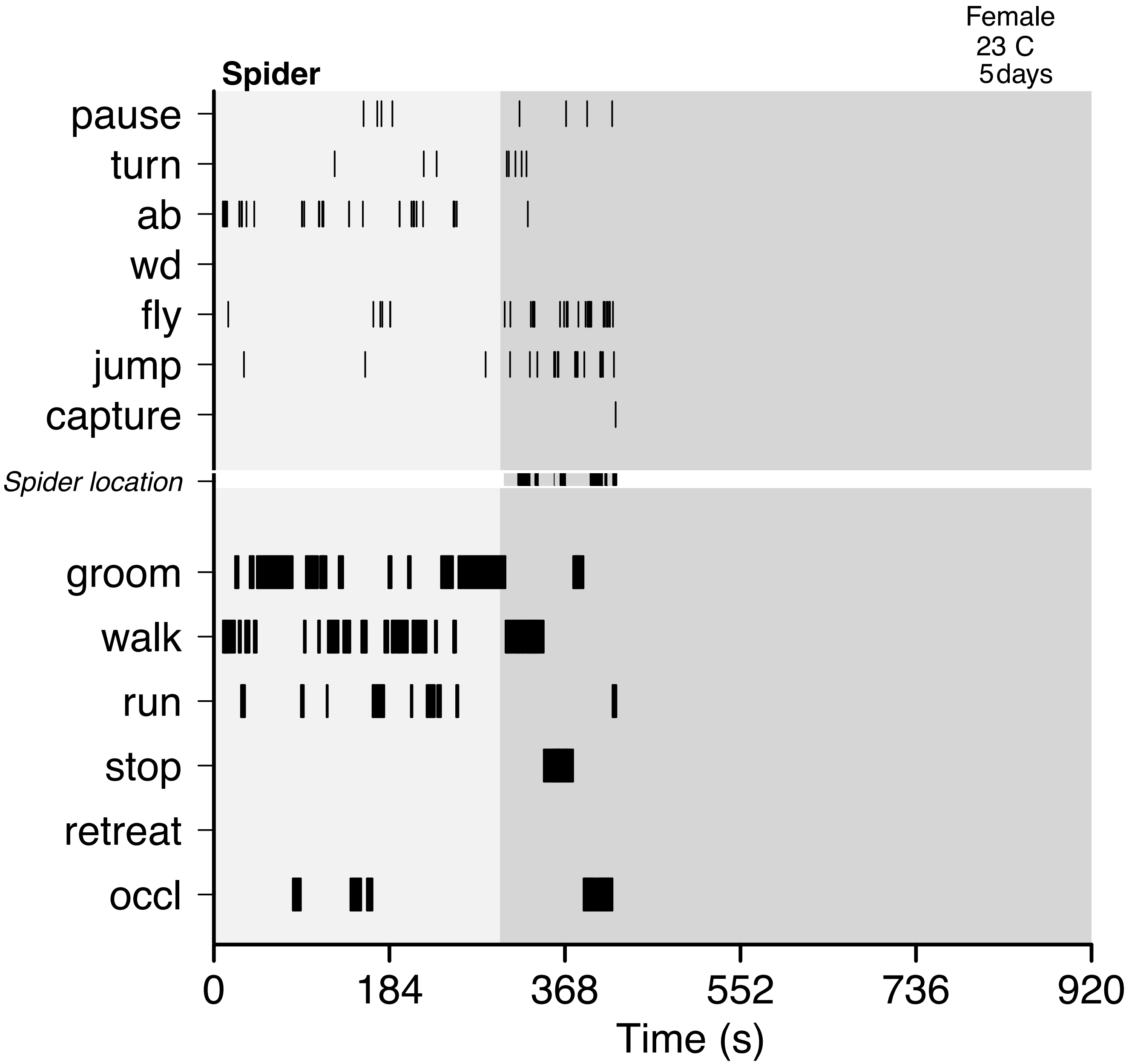

Female
23 C
5 days
Spider
pause
turn
ab
wd
fly
jump
capture
Spider location
groom
walk
run
stop
retreat
occl
0
184
368
552
736
920
Time (s)

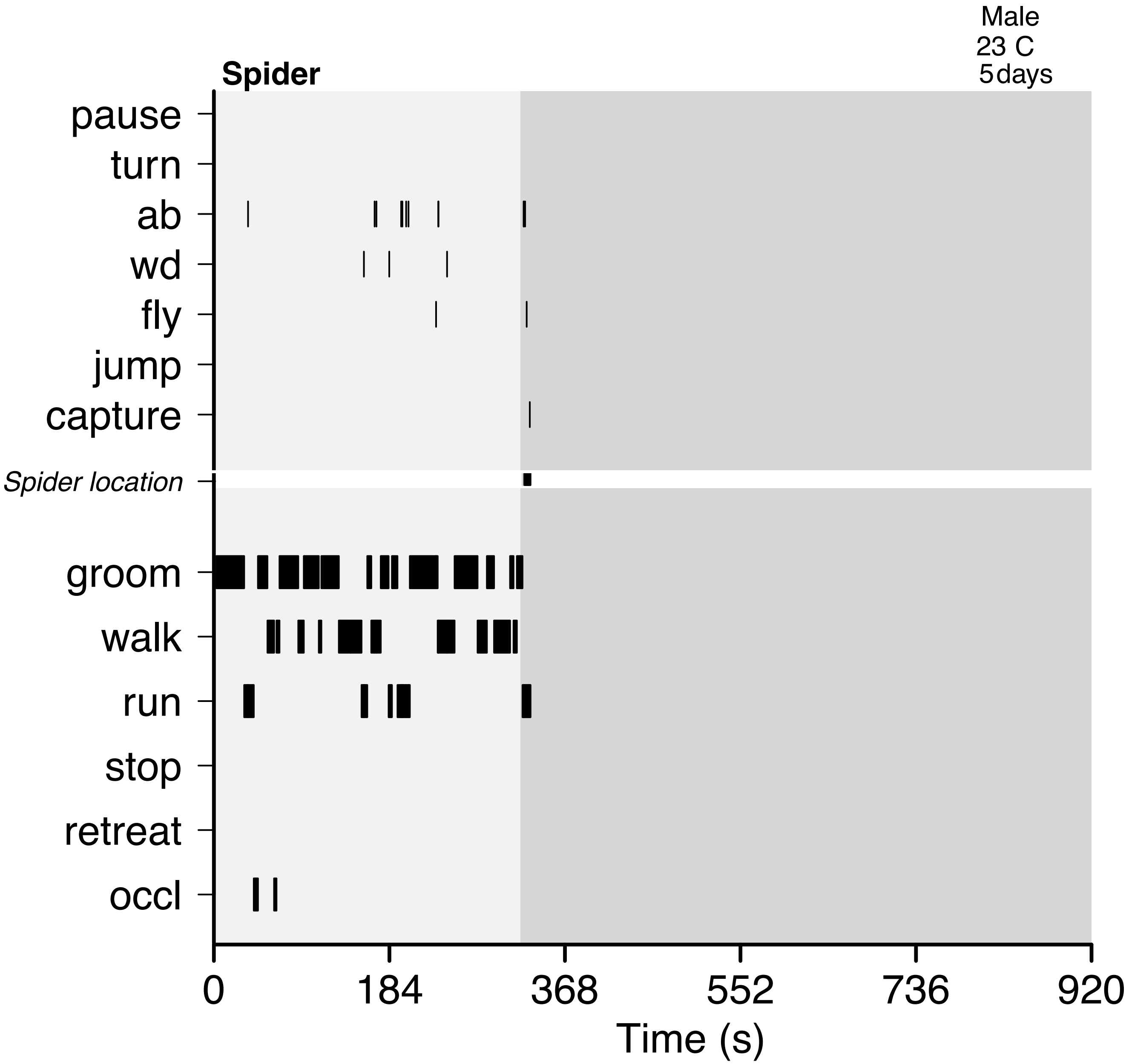

Male
23 C
5 days
Spider
pause
turn
ab
wd
fly
jump
capture
Spider location
groom
walk
run
stop
retreat
occl
0
184
368
552
736
920
Time (s)

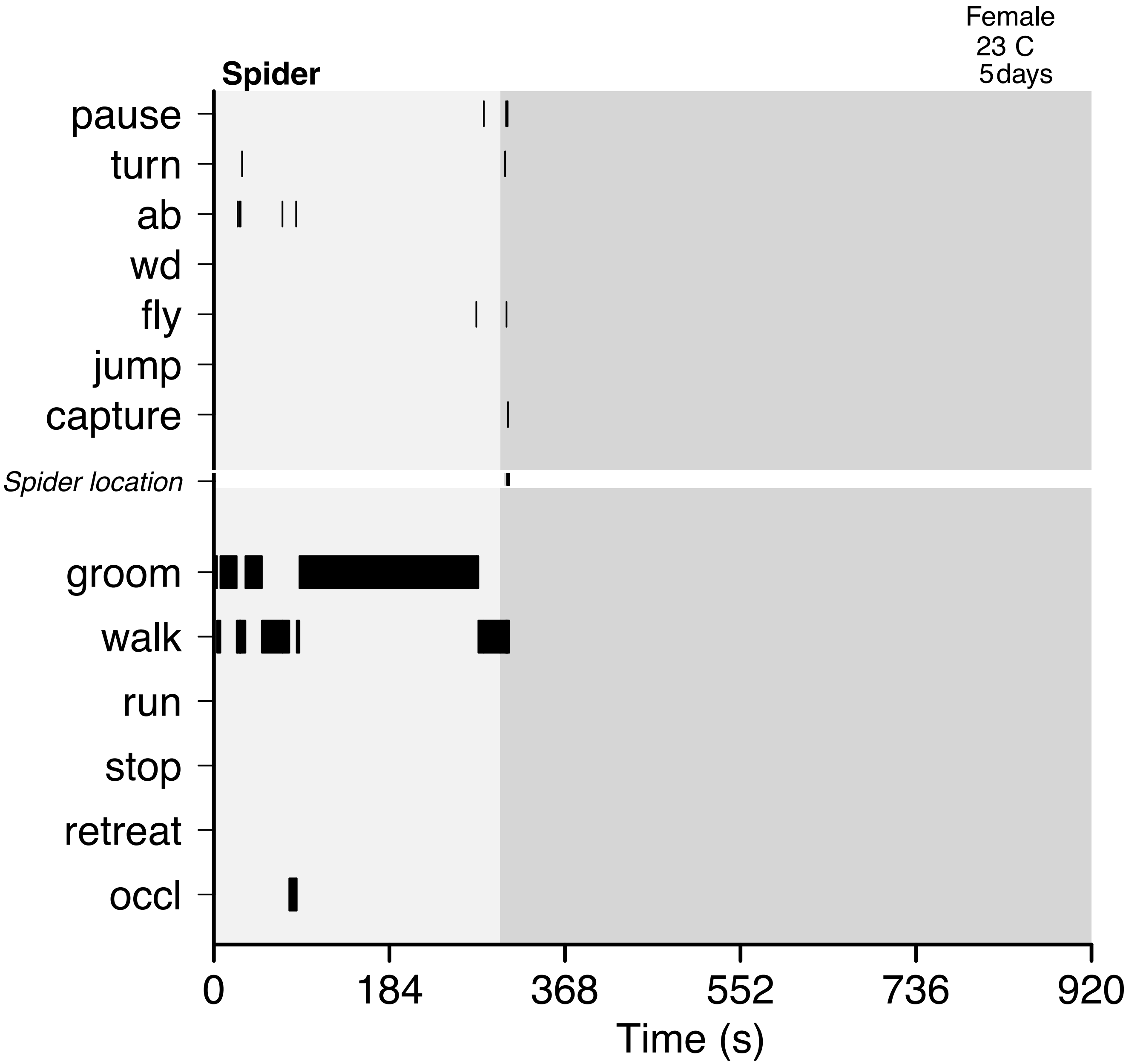
Female
23 C
5 days
Spider
pause
turn
ab
wd
fly
jump
capture
Spider location
groom
walk
run
stop
retreat
occl
0
184
368
552
736
920
Time (s)

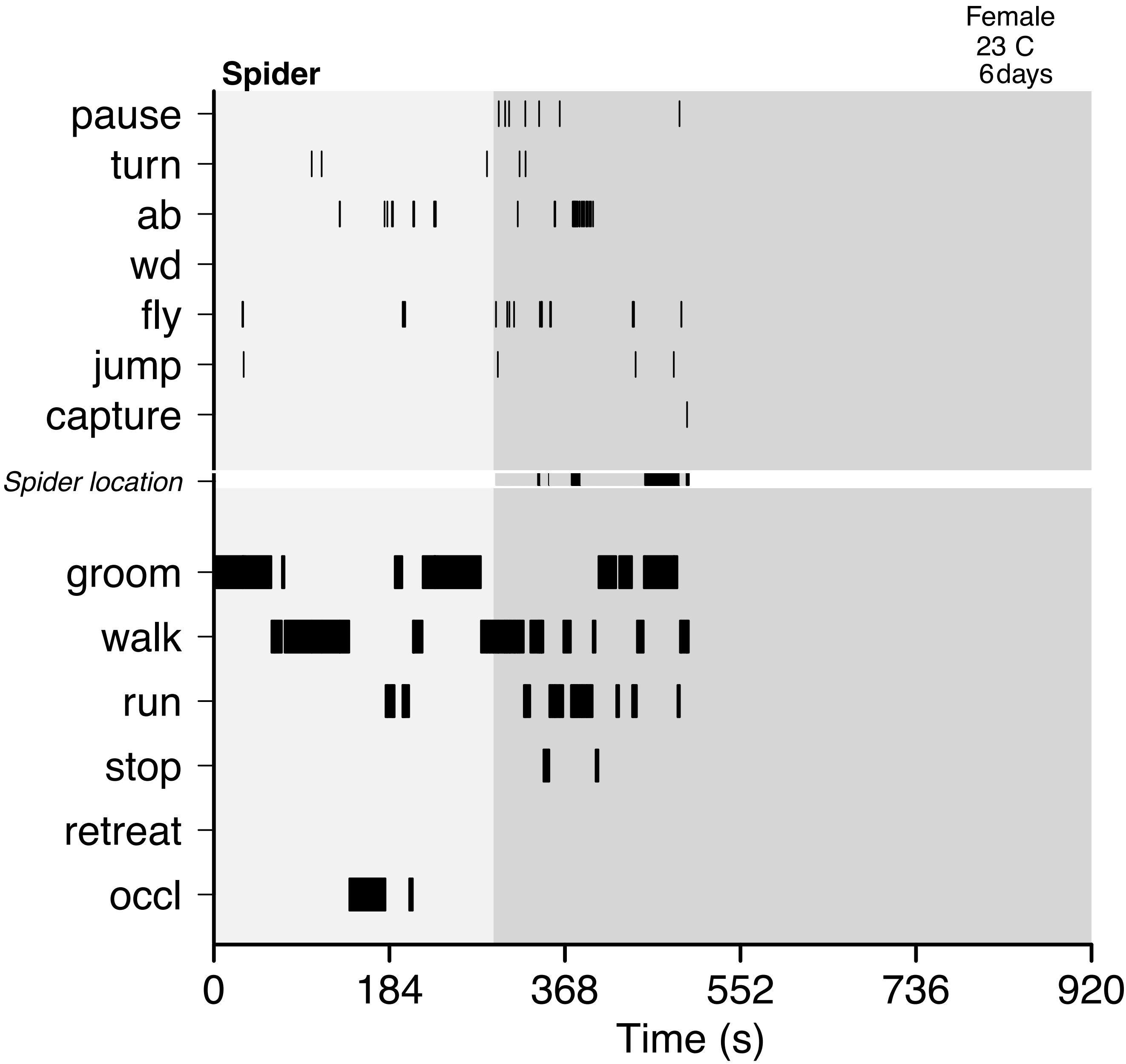
Female
23 C
6 days
Spider
pause
turn
ab
wd
fly
jump
capture
Spider location
groom
walk
run
stop
retreat
occl
0
184
368
552
736
920
Time (s)

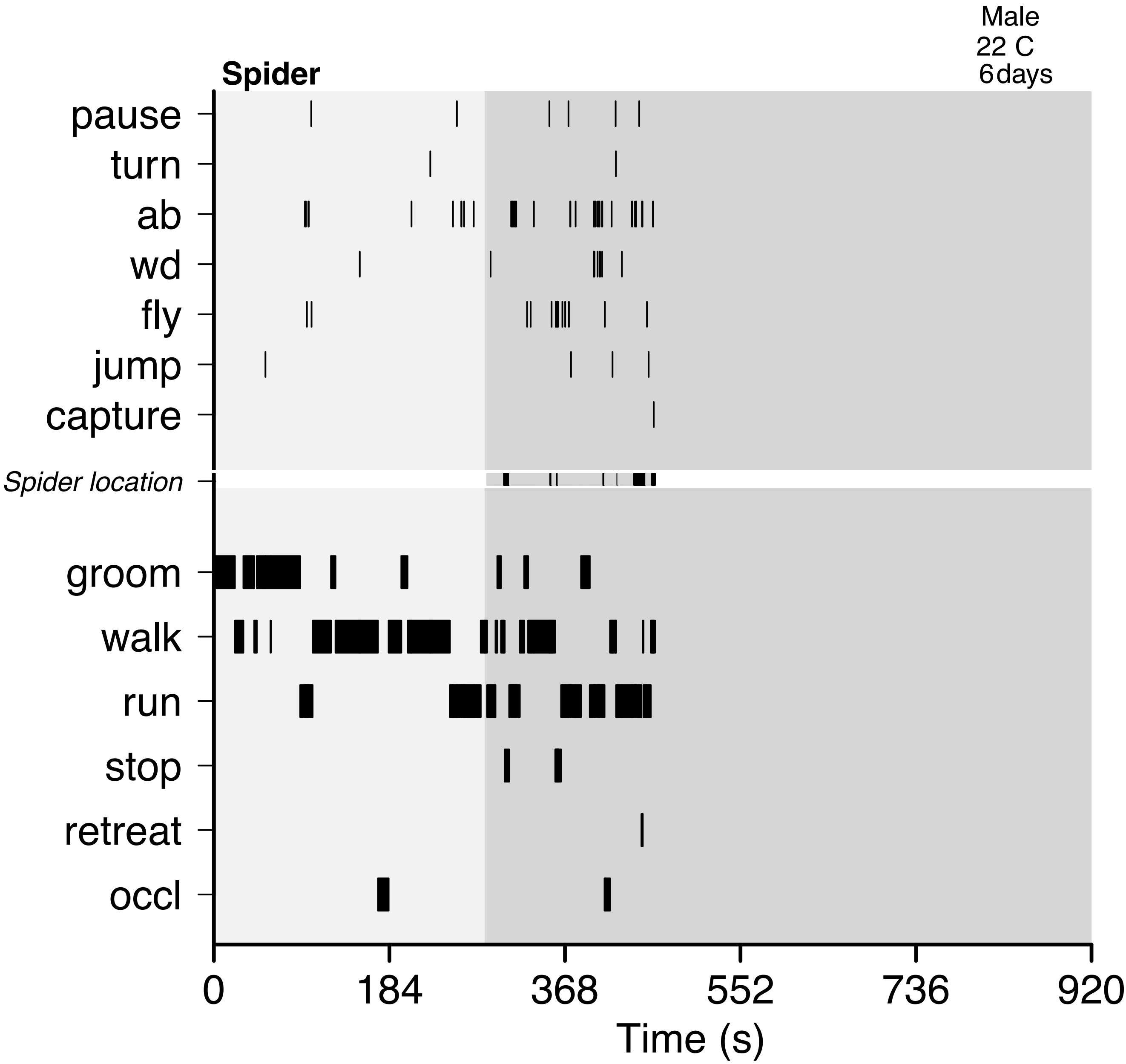

Male
22 C
6 days
Spider
pause
turn
ab
wd
fly
jump
capture
Spider location
groom
walk
run
stop
retreat
occl
0
184
368
552
736
920
Time (s)

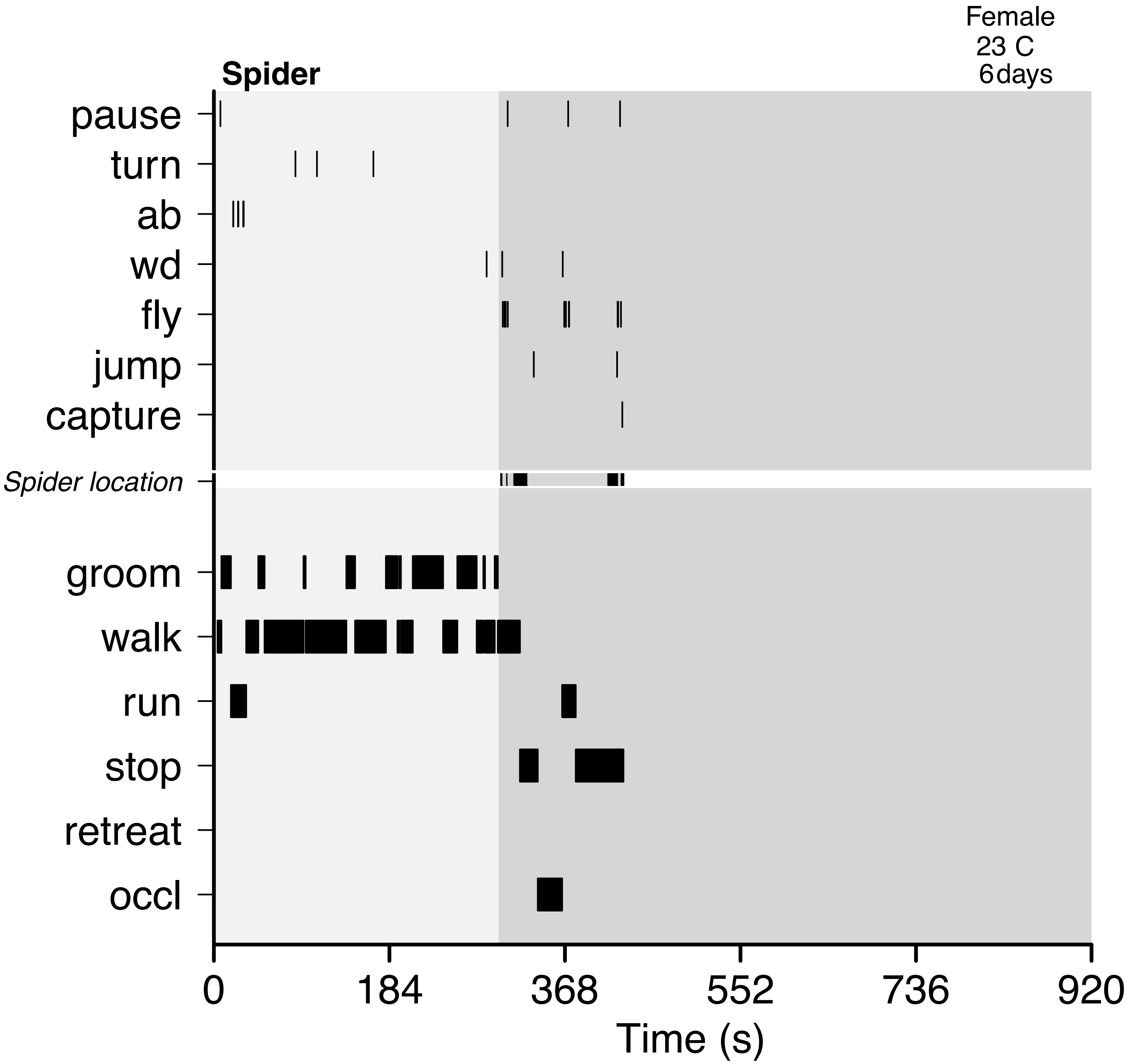
Female
23 C
6 days
Spider
pause
turn
ab
wd
fly
jump
capture
Spider location
groom
walk
run
stop
retreat
occl
0
184
368
552
736
920
Time (s)

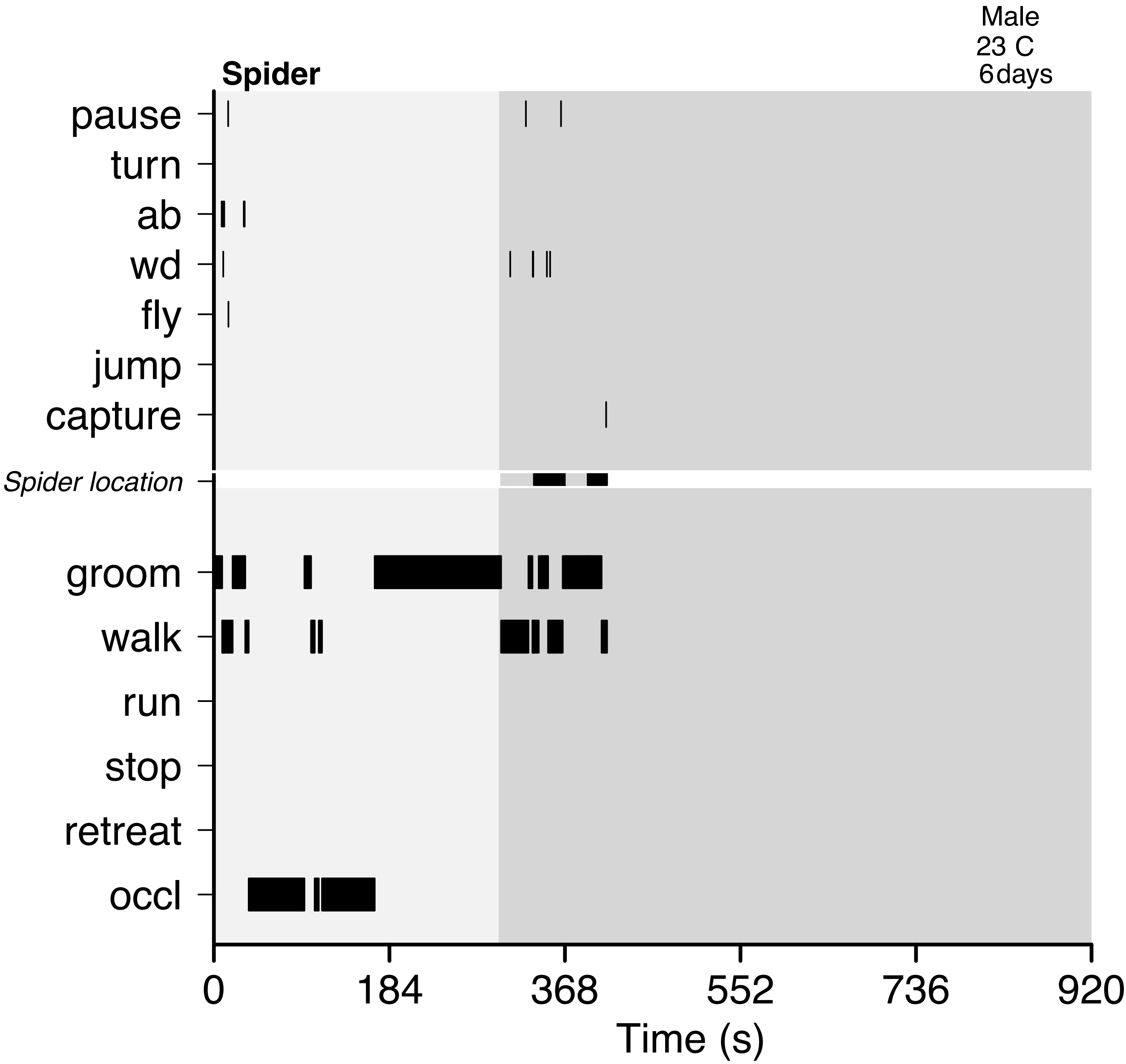
Male
23 C
6 days
Spider
pause
turn
ab
wd
fly
jump
capture
Spider location
groom
walk
run
stop
retreat
occl
0
184
368
552
736
920
Time (s)

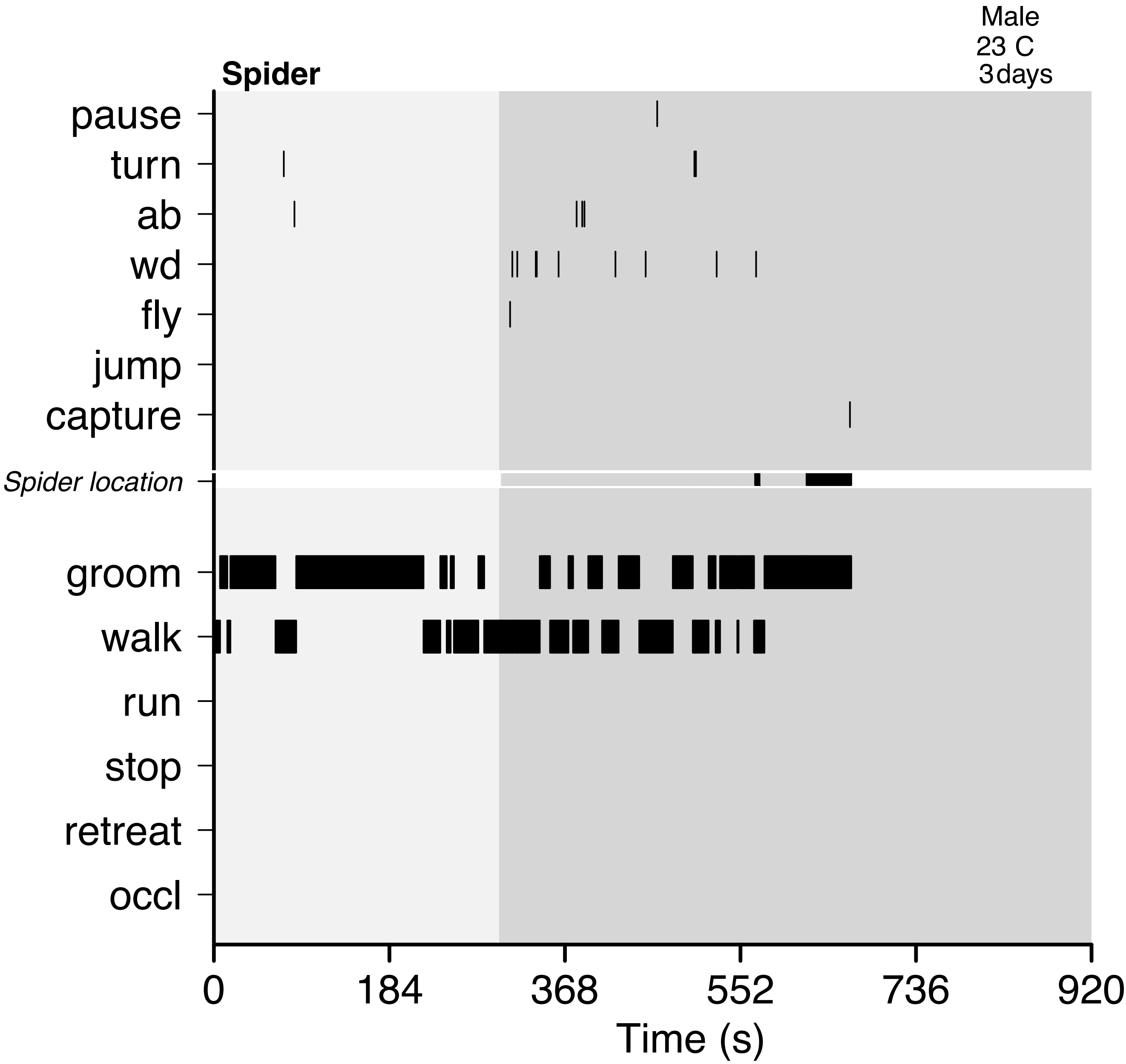
Male
23 C
3days
Spider
pause
turn
ab
wd
fly
jump
capture
Spider location
groom
walk
run
stop
retreat
occl
0
184
368
552
736
920
Time (s)

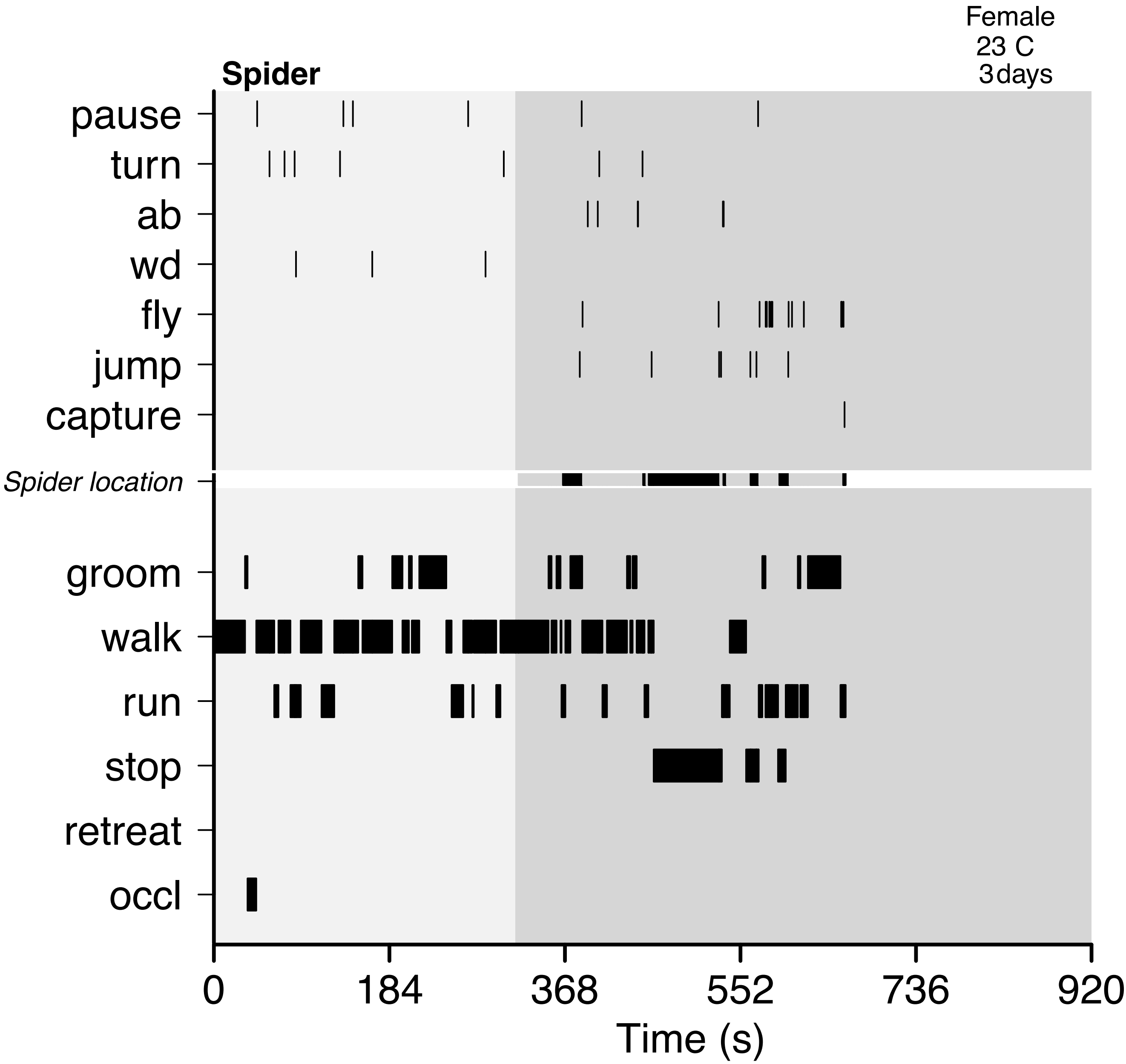
Female
23 C
3days
Spider
pause
turn
ab
wd
fly
jump
capture
Spider location
groom
walk
run
stop
retreat
occl
0
184
368
552
736
920
Time (s)

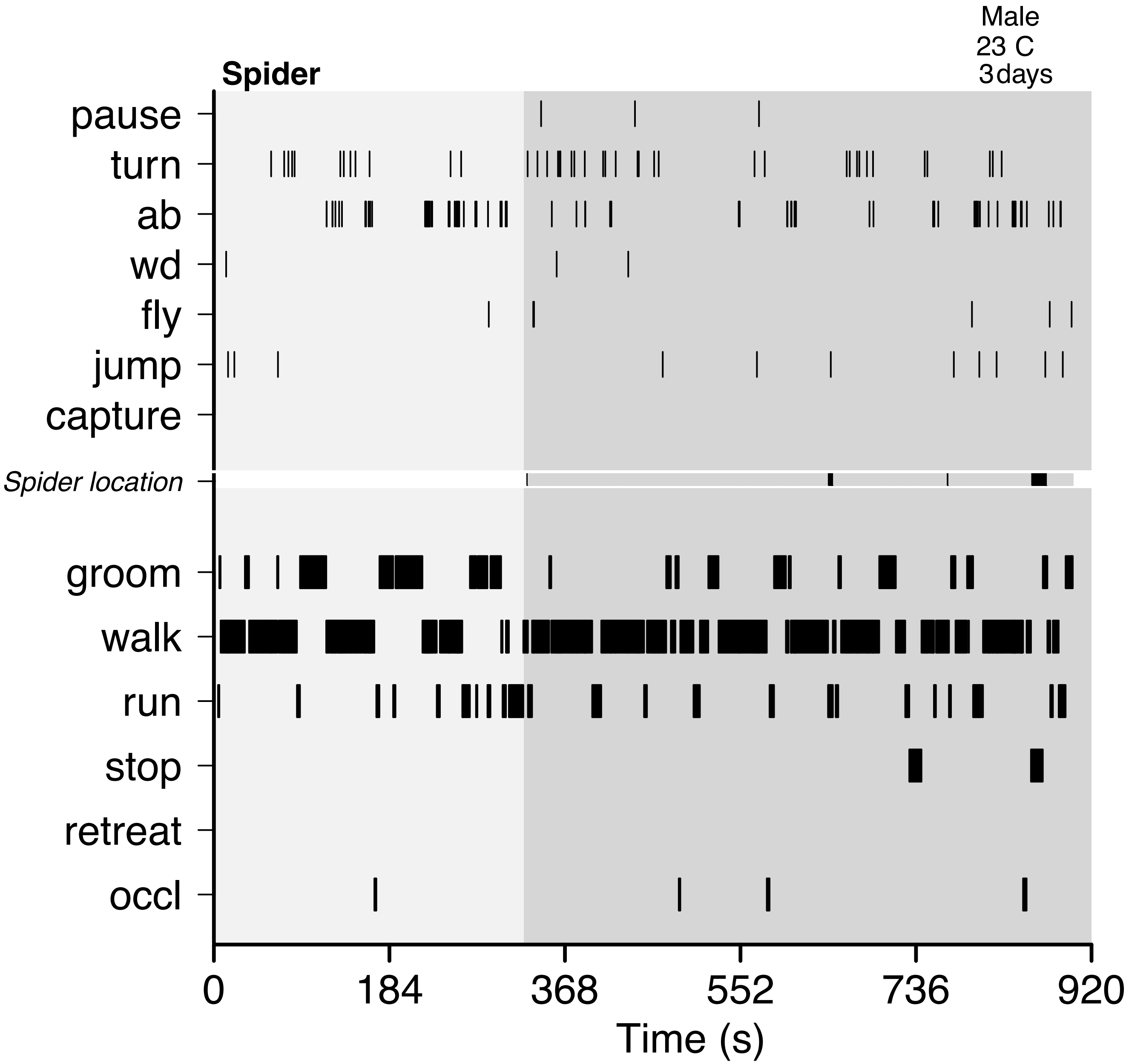

Male
23 C
3days
Spider
pause
turn
ab
wd
fly
jump
capture
Spider location
groom
walk
run
stop
retreat
occl
0
184
368
552
736
920
Time (s)

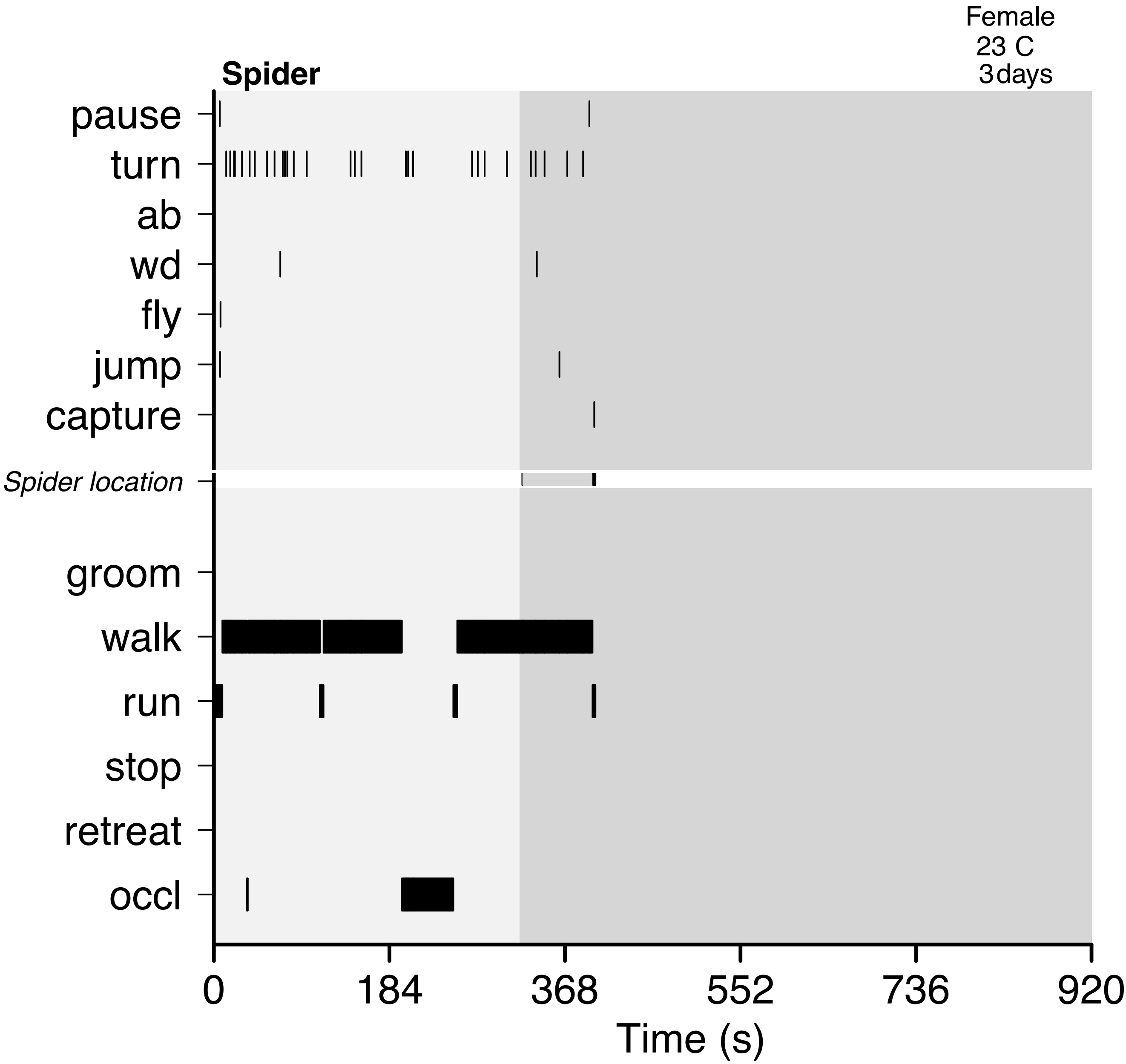
Female
23 C
3days
Spider
pause
turn
ab
wd
fly
jump
capture
Spider location
groom
walk
run
stop
retreat
occl
0
184
368
552
736
920
Time (s)

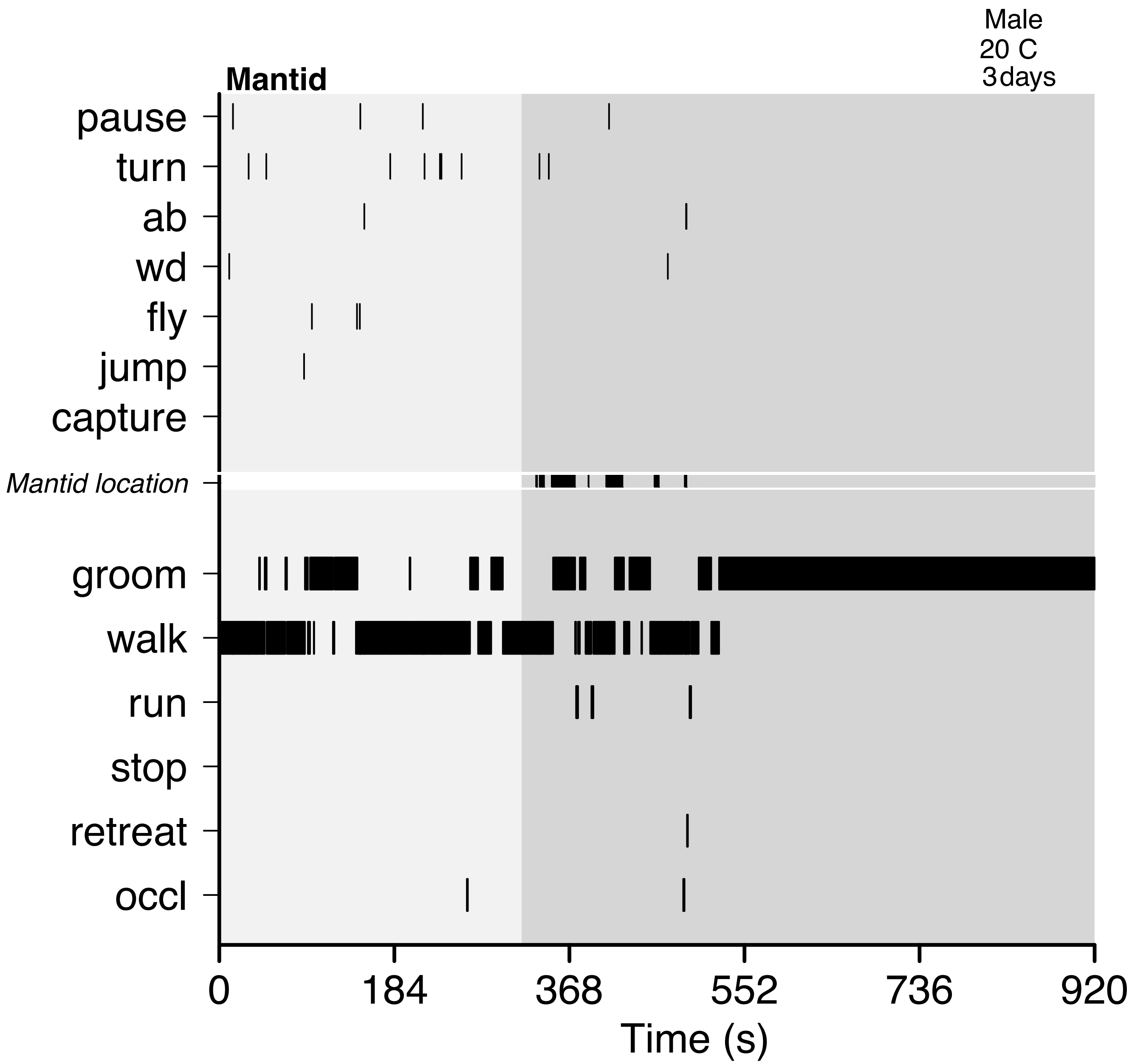

Male
20 C
3days
Mantid
pause
turn
ab
wd
fly
jump
capture
Mantid location
groom
walk
run
stop
retreat
occl
0
184
368
552
736
920
Time (s)

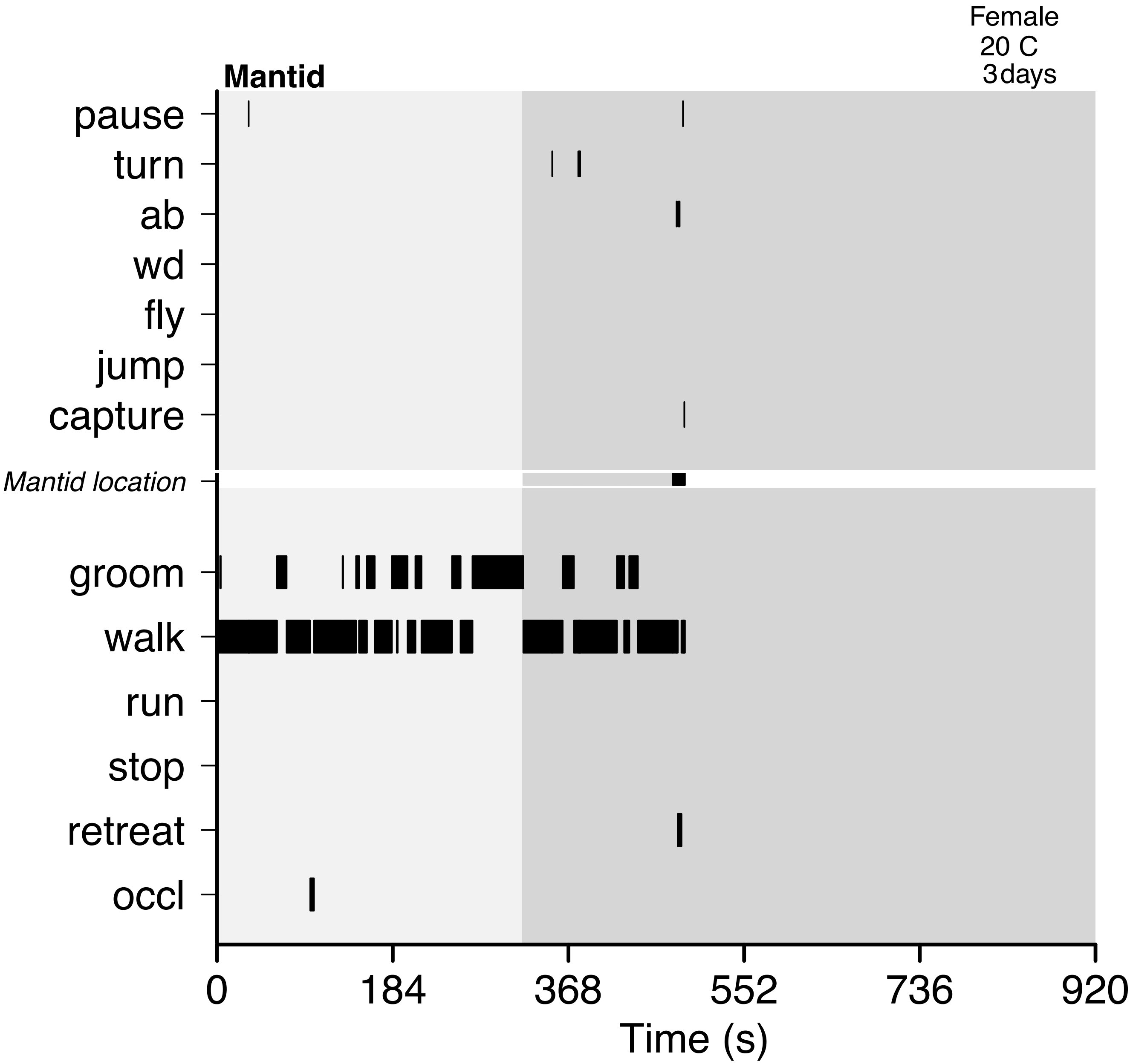
Female
20 C
3days
Mantid
pause
turn
ab
wd
fly
jump
capture
Mantid location
groom
walk
run
stop
retreat
occl
0
184
368
552
736
920
Time (s)

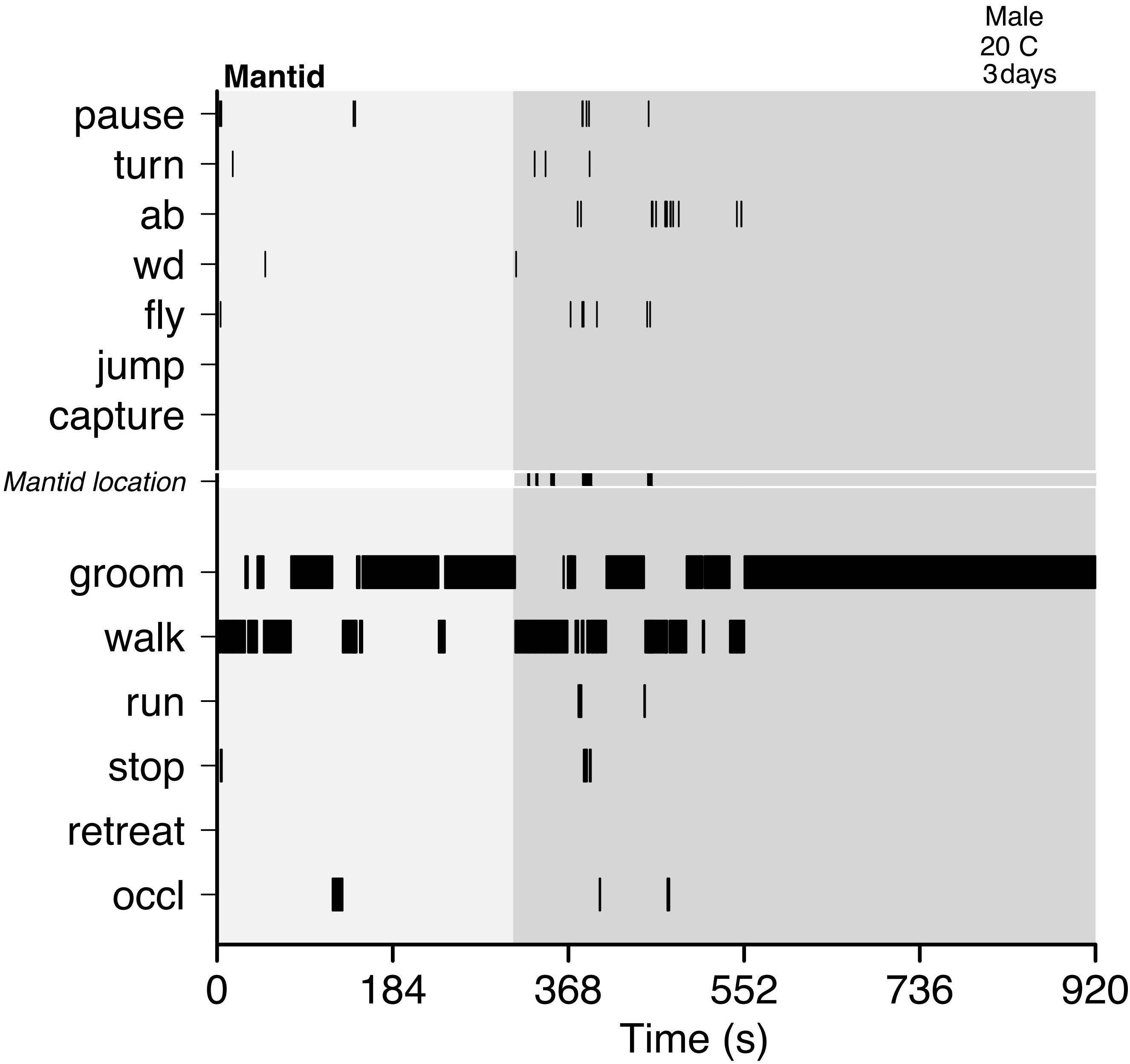

Male
20 C
3days
Mantid
pause
turn
ab
wd
fly
jump
capture
Mantid location
groom
walk
run
stop
retreat
occl
0
184
368
552
736
920
Time (s)

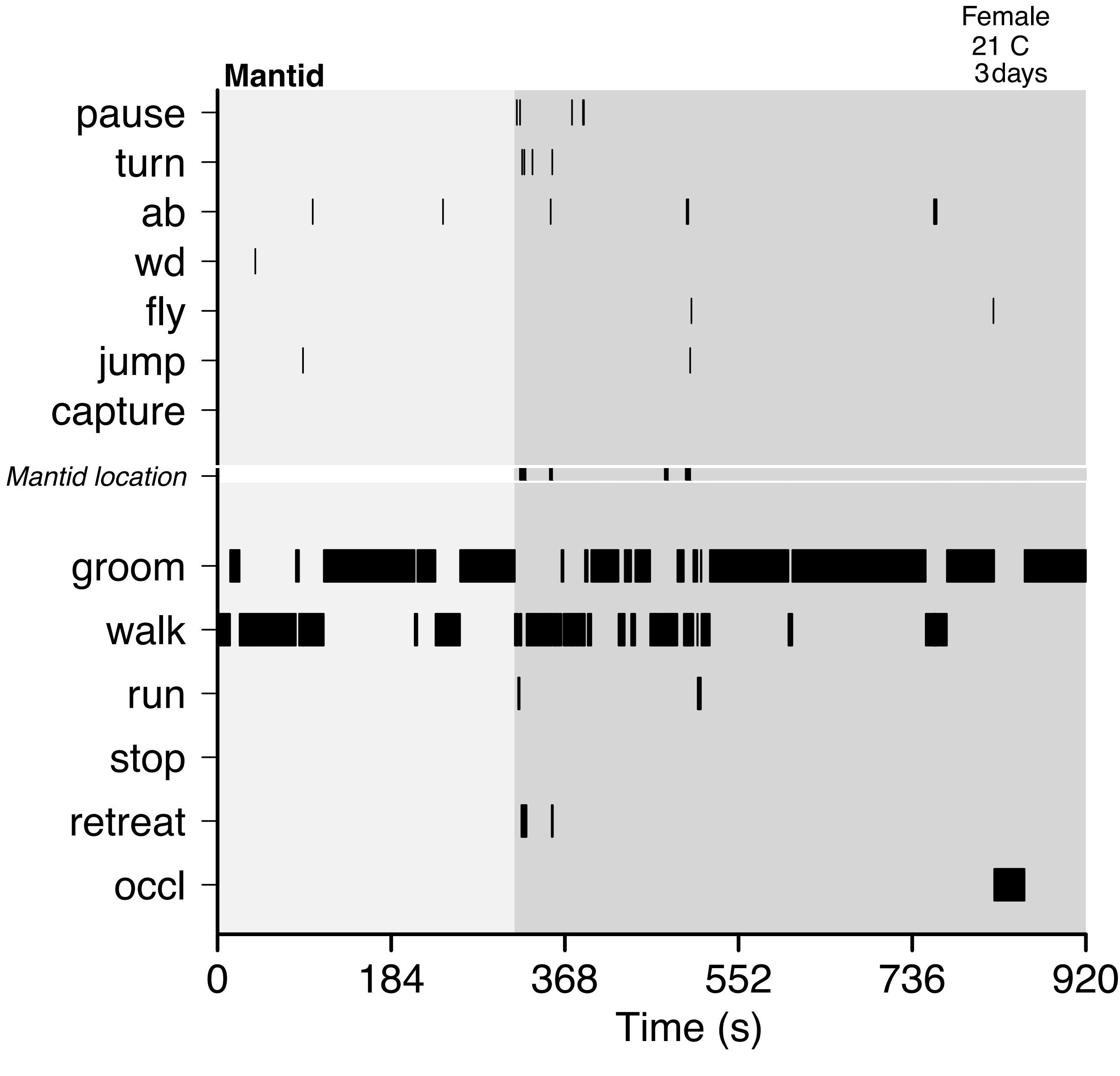

Female
21 C
3days
Mantid
pause
turn
ab
wd
fly
jump
capture
Mantid location
groom
walk
run
stop
retreat
occl
0
184
368
552
736
920
Time (s)

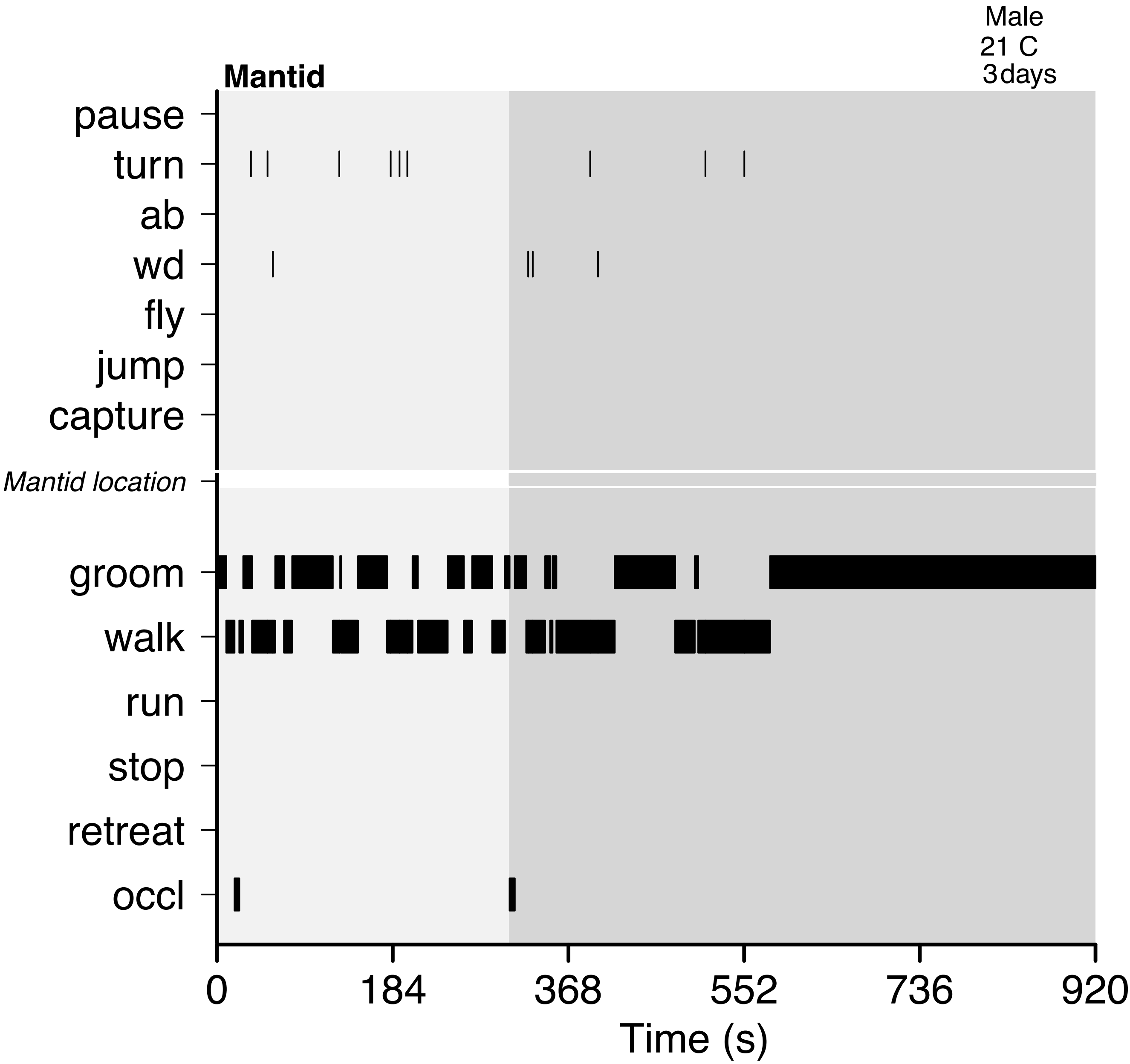

Male
21 C
3days
Mantid
pause
turn
ab
wd
fly
jump
capture
Mantid location
groom
walk
run
stop
retreat
occl
0
184
368
552
736
920
Time (s)

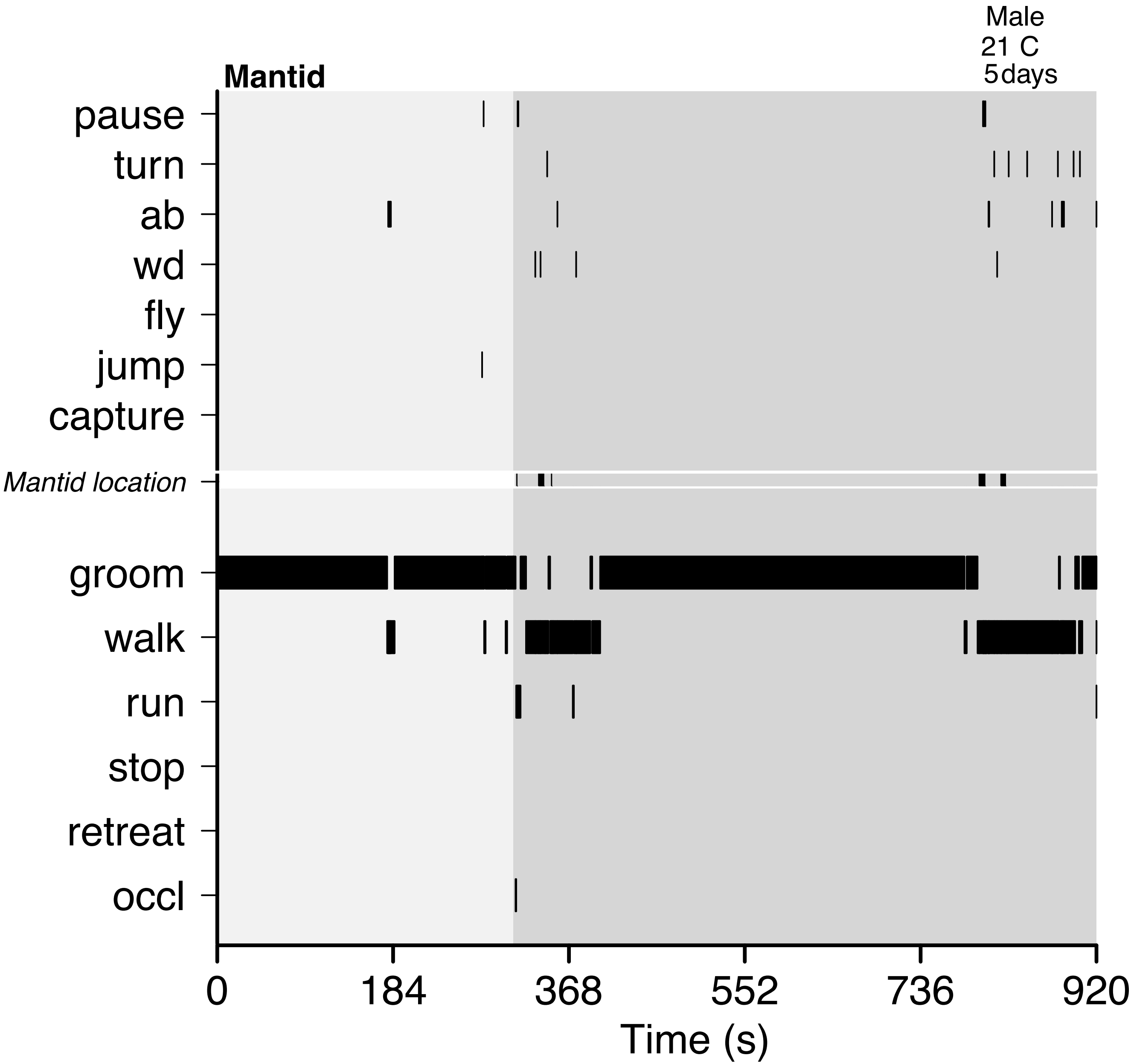
Male
21 C
5 days
Mantid
pause
turn
ab
wd
fly
jump
capture
Mantid location
groom
walk
run
stop
retreat
occl
0
184
368
552
736
920
Time (s)

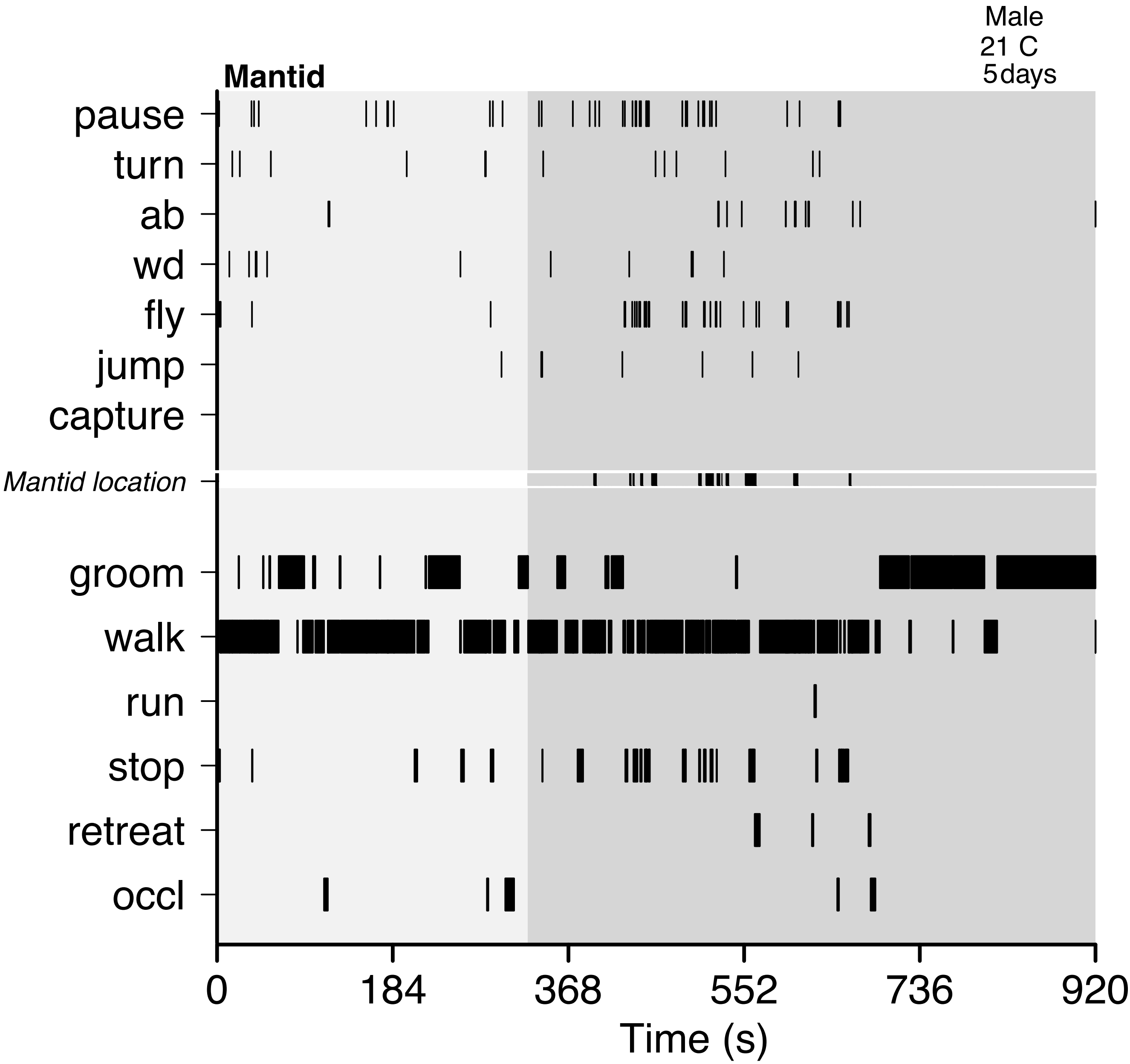

Male
21 C
5 days
Mantid
pause
turn
ab
wd
fly
jump
capture
Mantid location
groom
walk
run
stop
retreat
occl
0
184
368
552
736
920
Time (s)

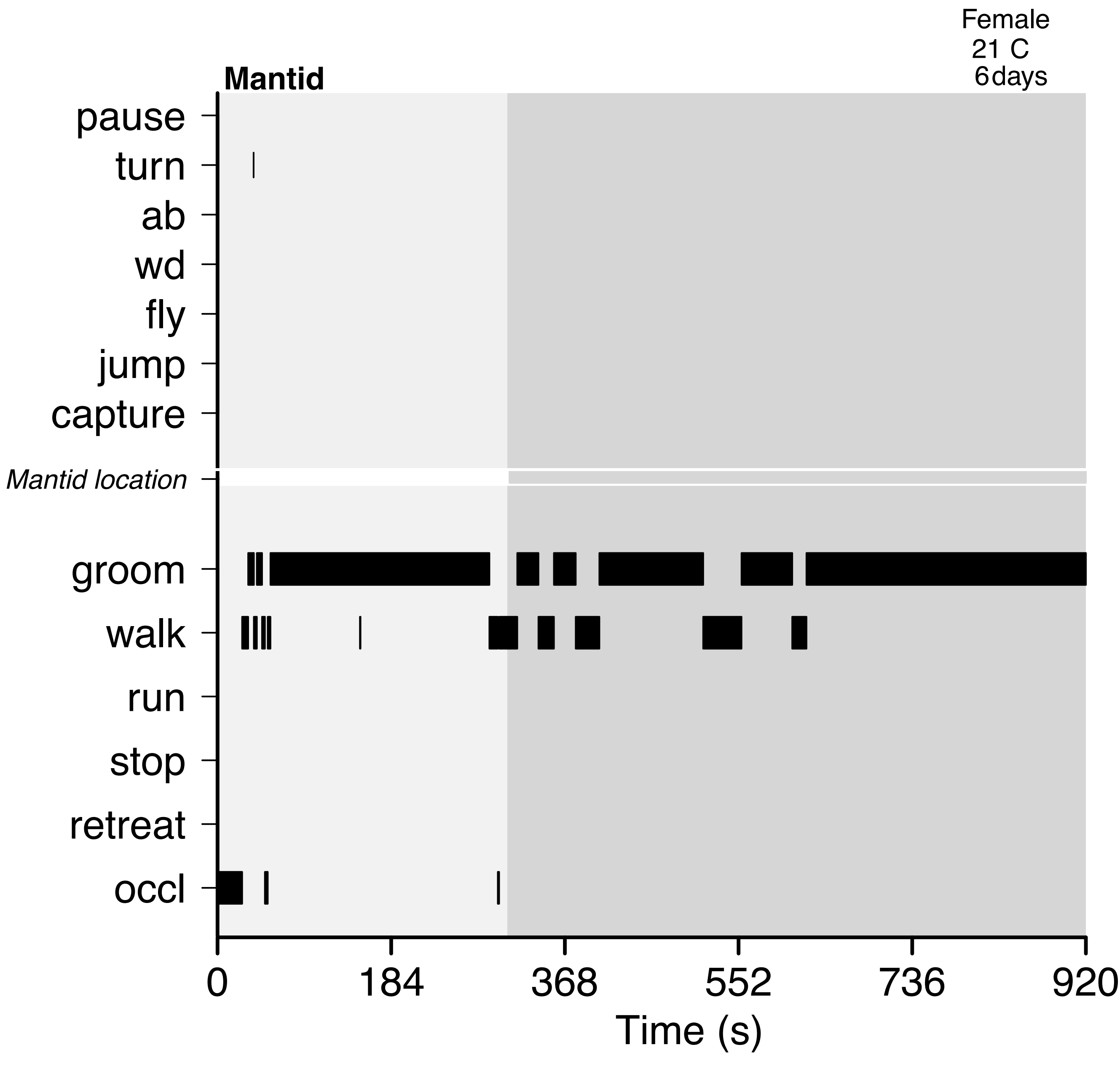
Female
21 C
6 days
Mantid
pause
turn
ab
wd
fly
jump
capture
Mantid location
groom
walk
run
stop
retreat
occl
0
184
368
552
736
920
Time (s)

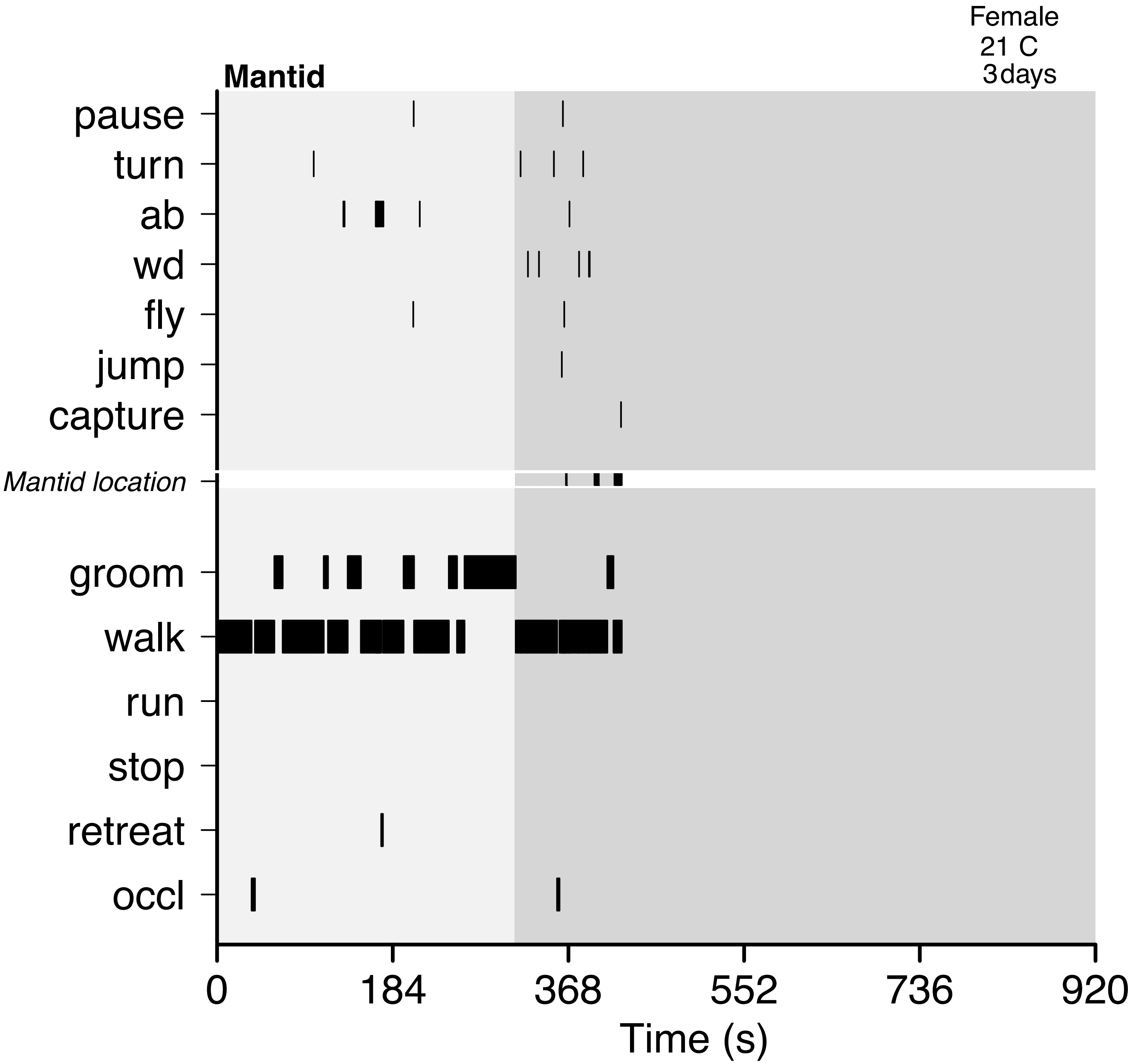

Female
21 C
3days
Mantid
pause
turn
ab
wd
fly
jump
capture
Mantid location
groom
walk
run
stop
retreat
occl
0
184
368
552
736
920
Time (s)

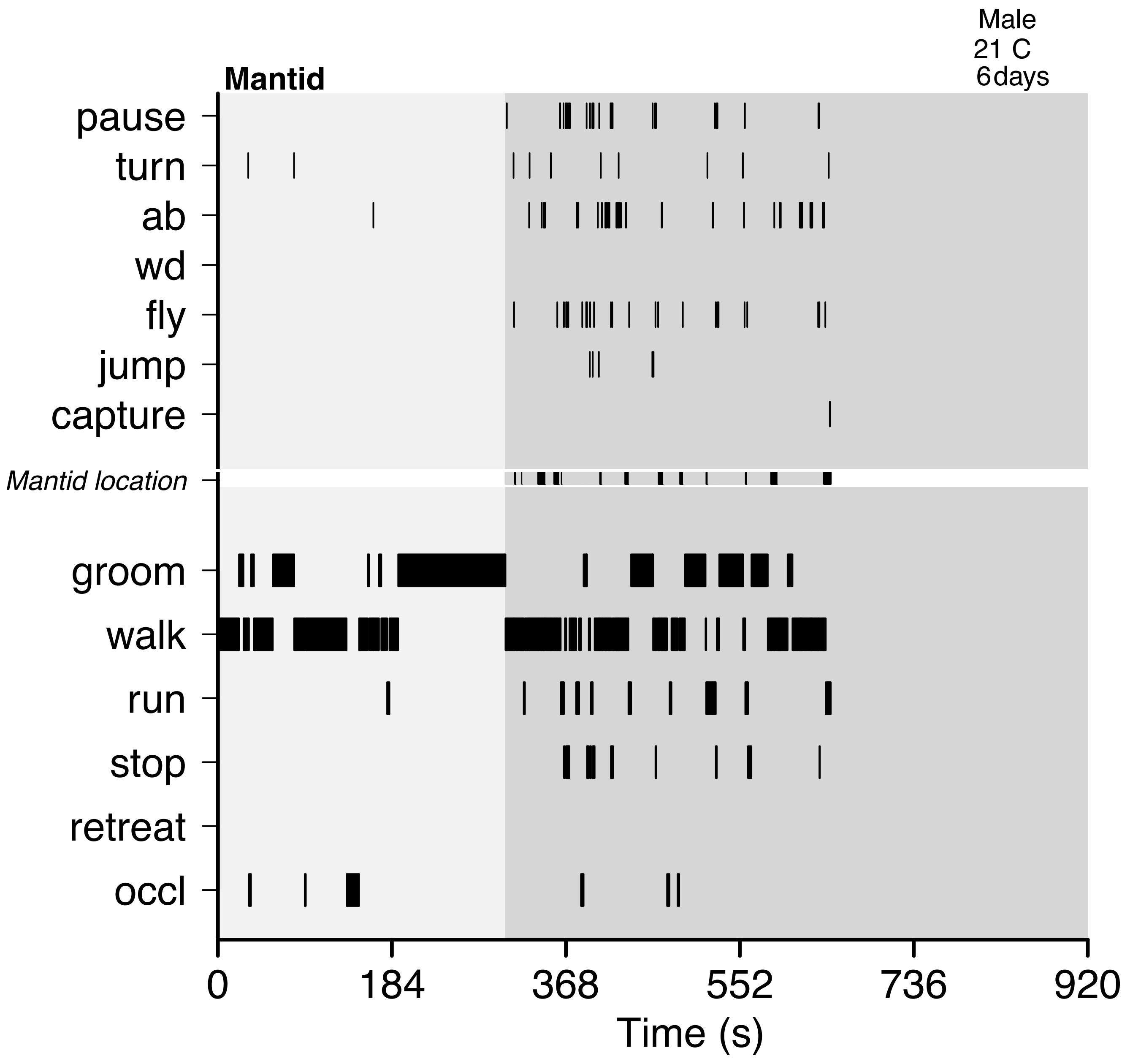

Male
21 C
6 days
Mantid
pause
turn
ab
wd
fly
jump
capture
Mantid location
groom
walk
run
stop
retreat
occl
0
184
368
552
736
920
Time (s)

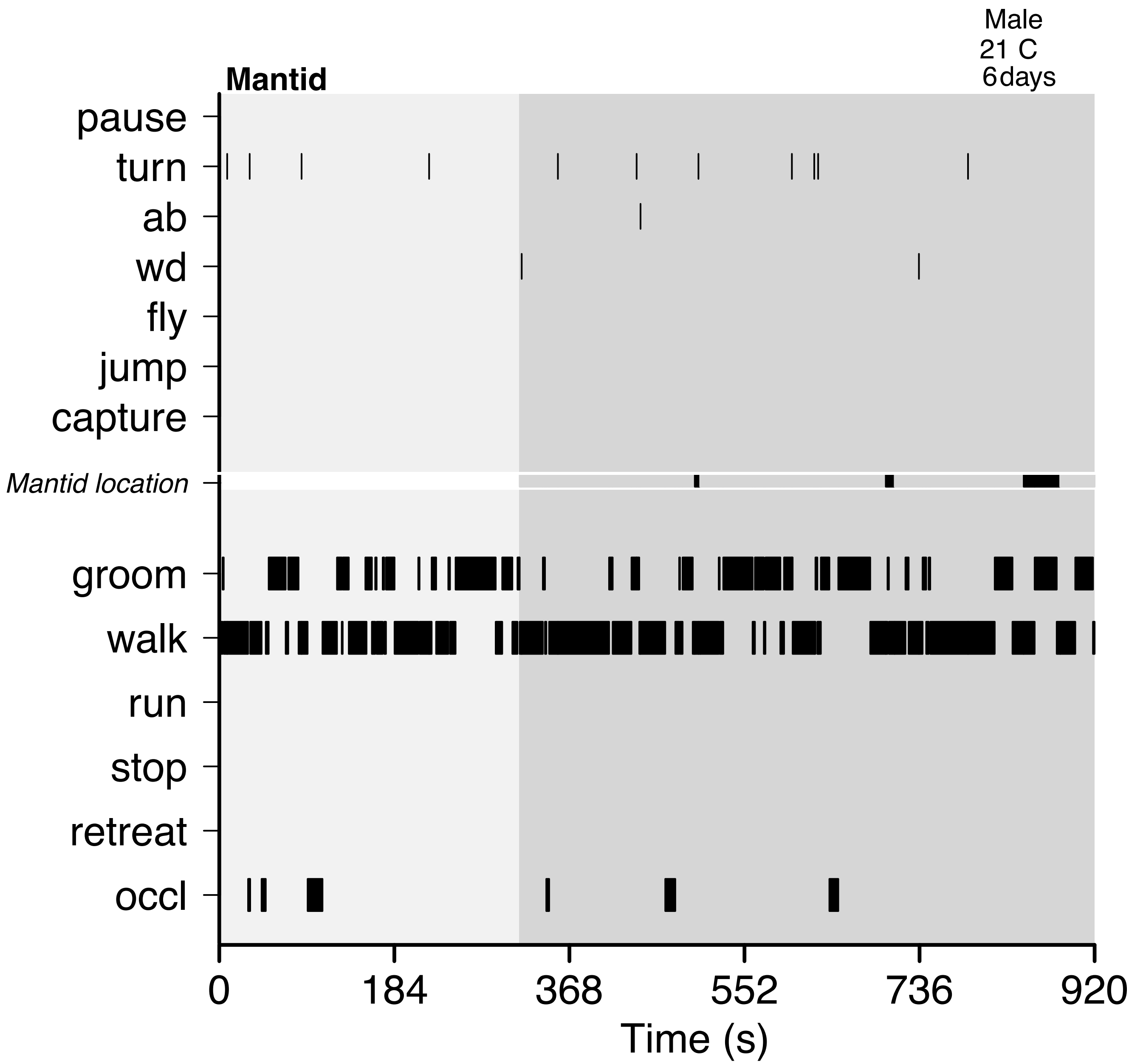
Male
21 C
6 days
Mantid
pause
turn
ab
wd
fly
jump
capture
Mantid location
groom
walk
run
stop
retreat
occl
0
184
368
552
736
920
Time (s)

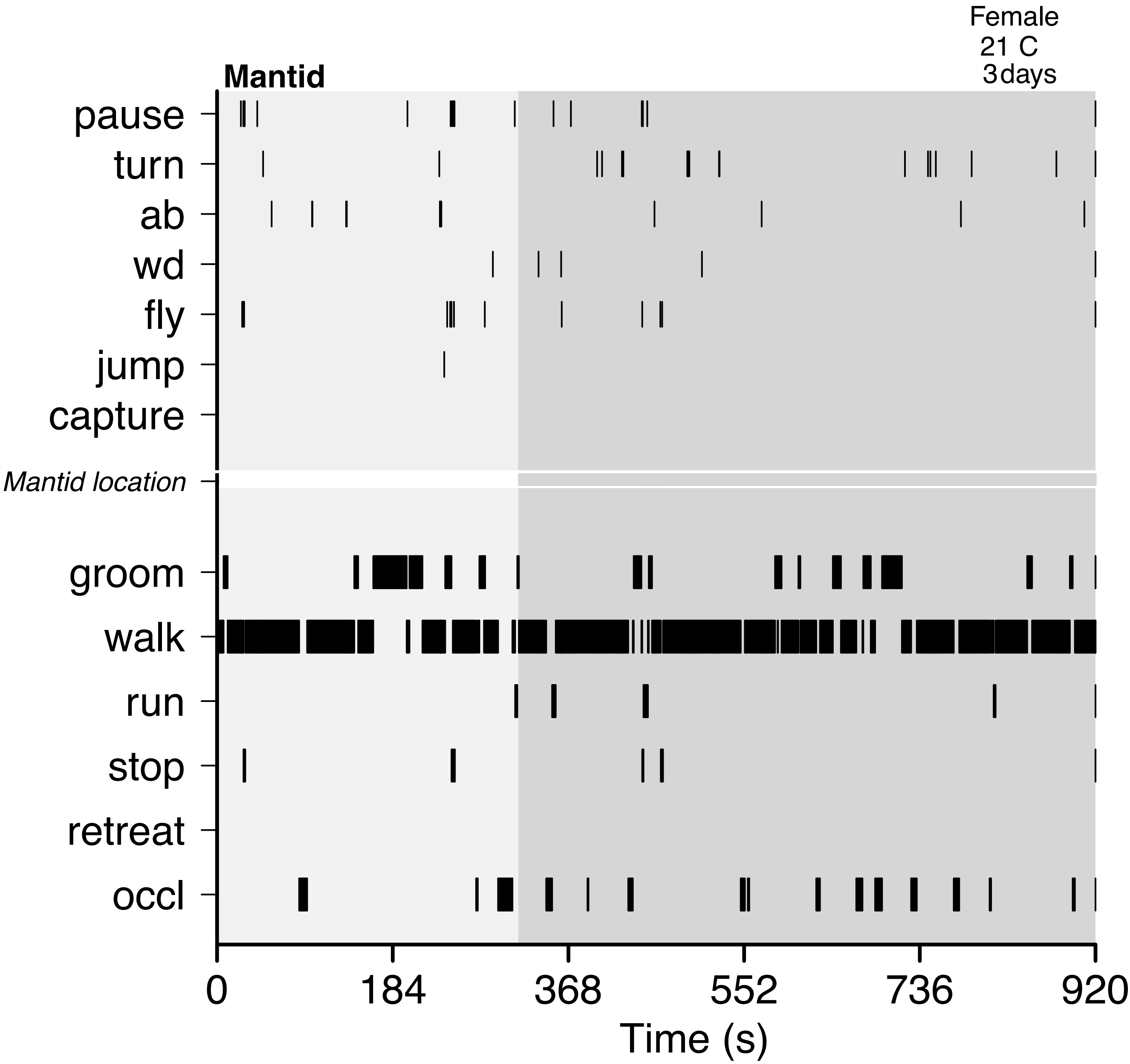

Female
21 C
3days
Mantid
pause
turn
ab
wd
fly
jump
capture
Mantid location
groom
walk
run
stop
retreat
occl
0
184
368
552
736
920
Time (s)

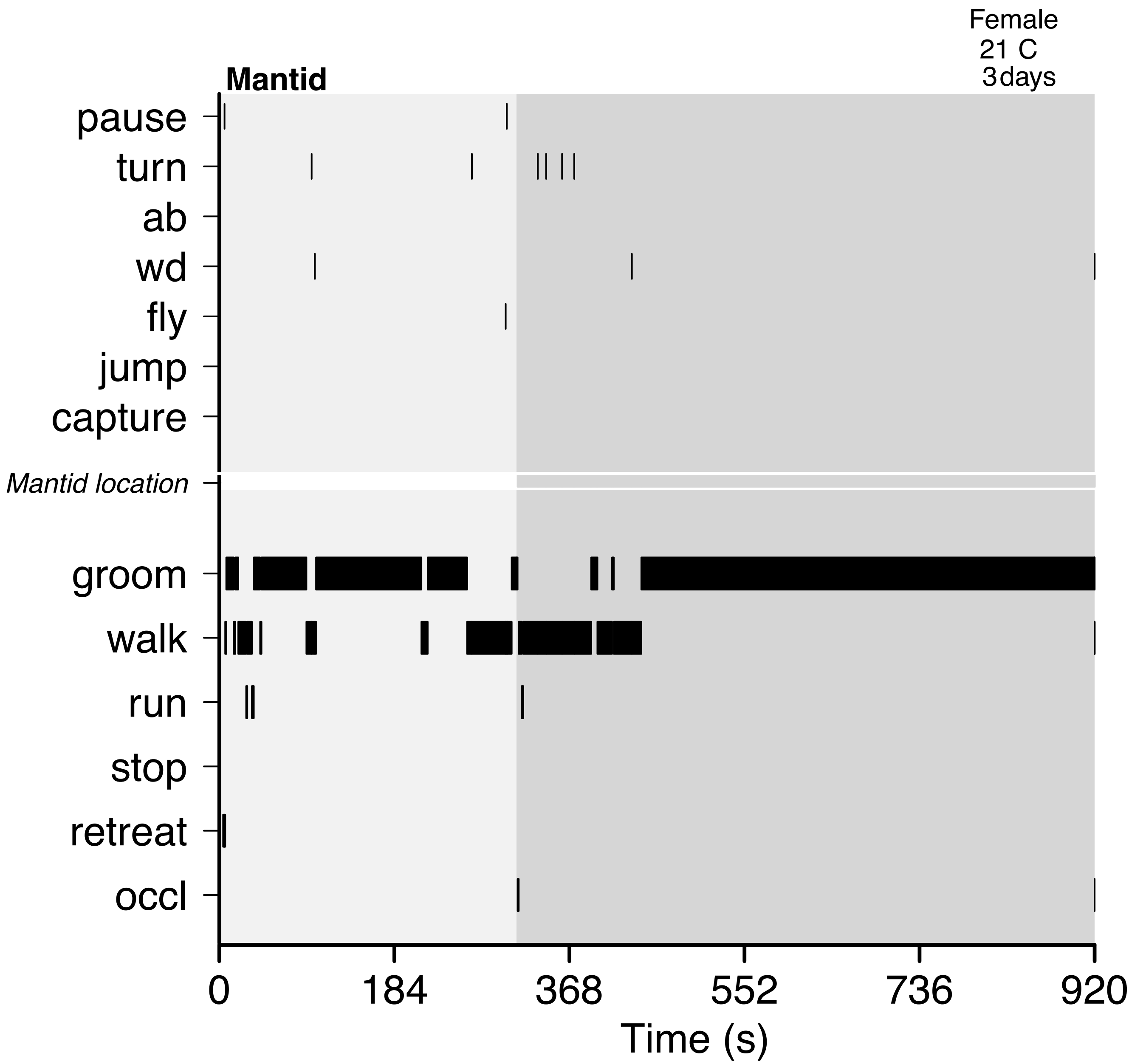
Female
21 C
3days
Mantid
pause
turn
ab
wd
fly
jump
capture
Mantid location
groom
walk
run
stop
retreat
occl
0
184
368
552
736
920
Time (s)

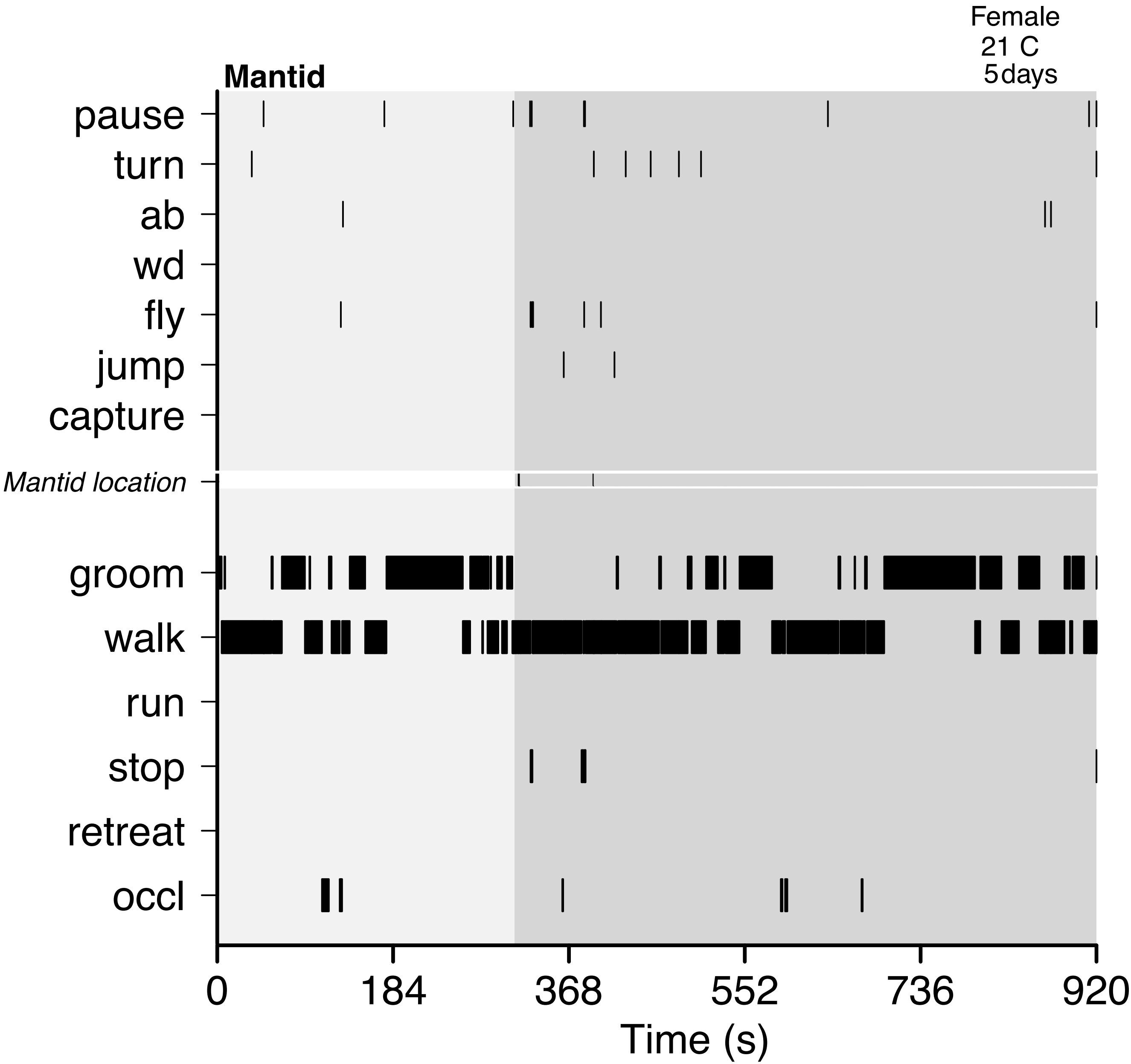

Female
21 C
5 days
Mantid
pause
turn
ab
wd
fly
jump
capture
Mantid location
groom
walk
run
stop
retreat
occl
0
184
368
552
736
920
Time (s)

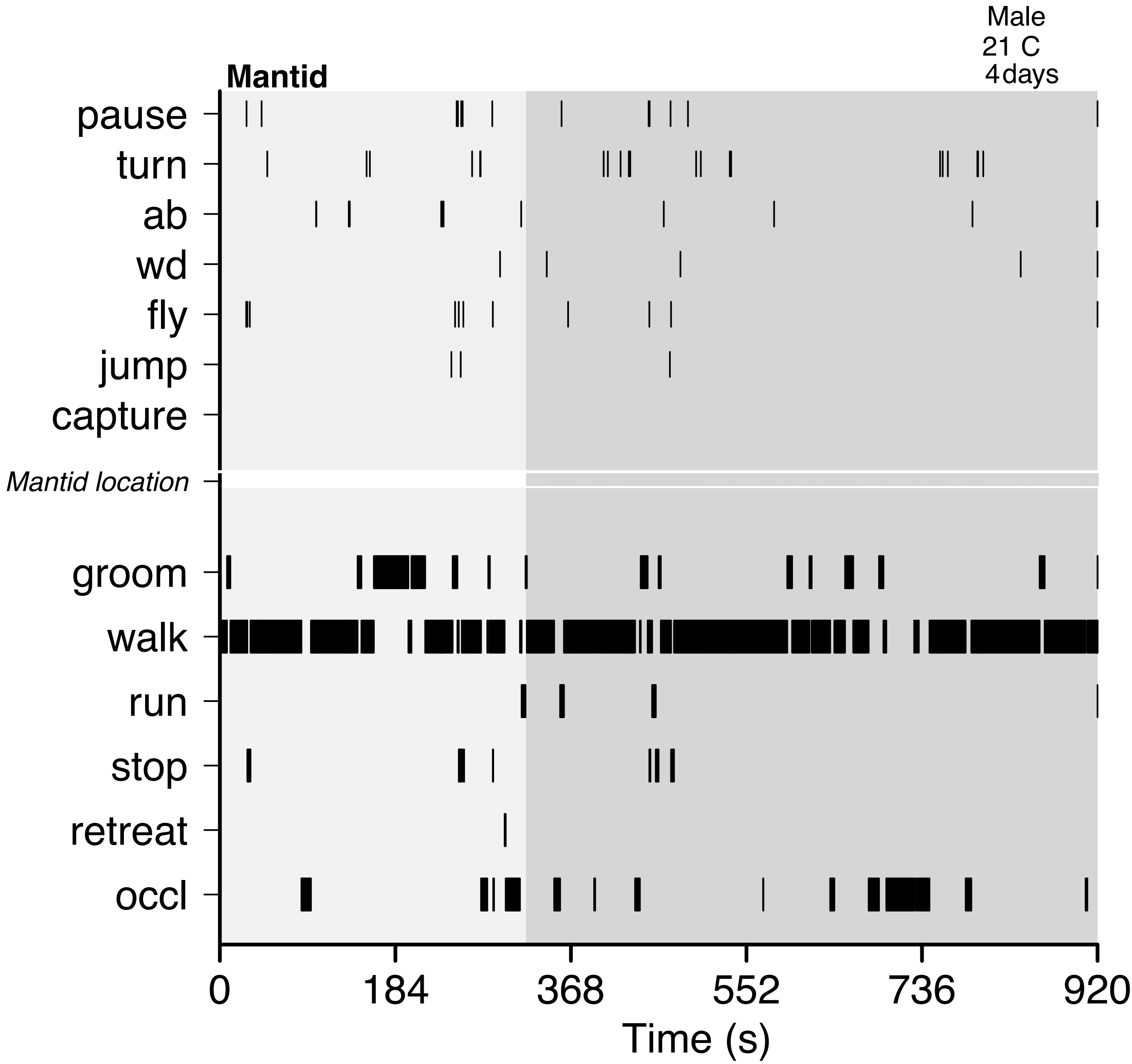

Male
21 C
4days
Mantid
pause
turn
ab
wd
fly
jump
capture
Mantid location
groom
walk
run
stop
retreat
occl
0
184
368
552
736
920
Time (s)

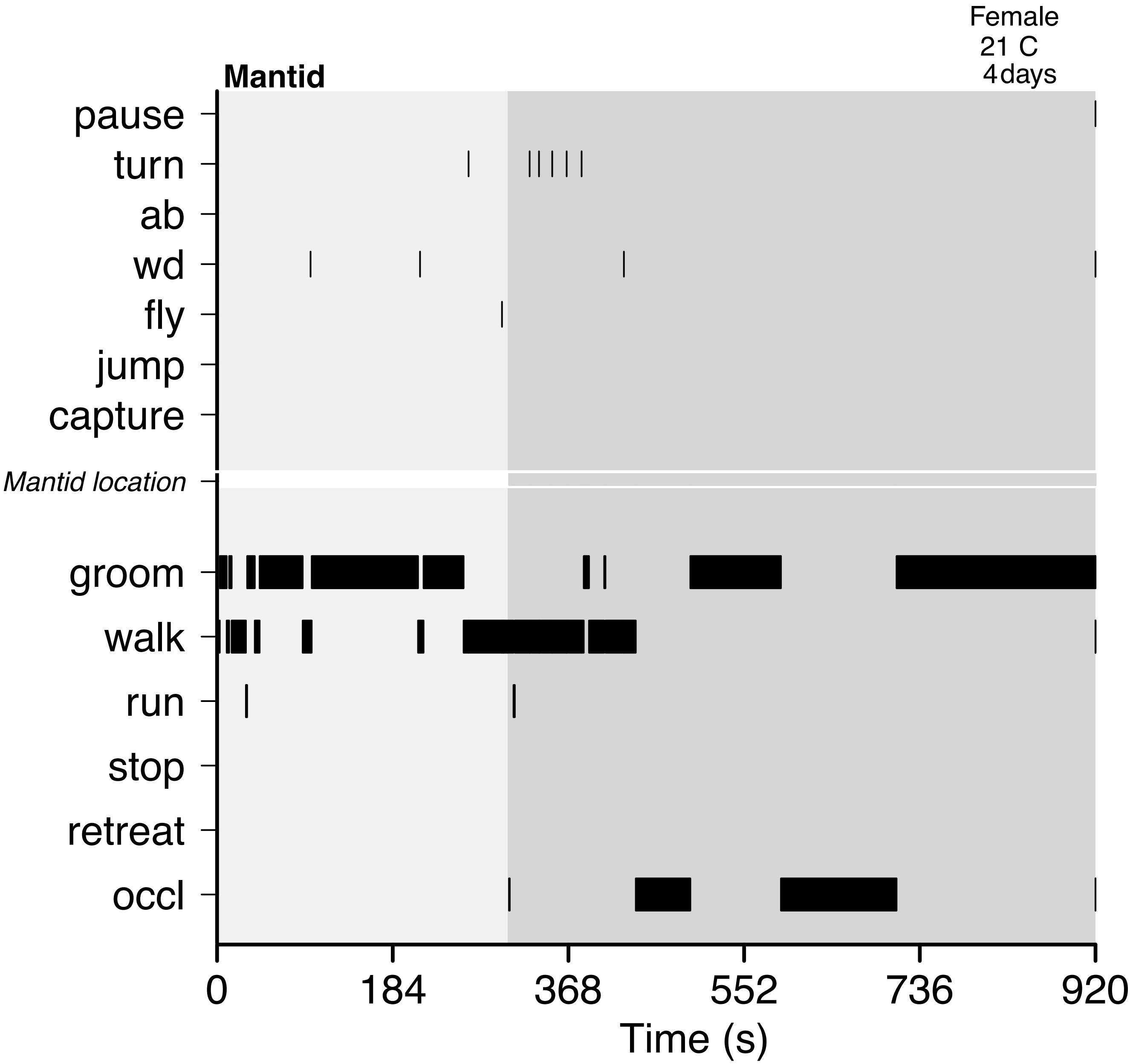
Female
21 C
4days
Mantid
pause
turn
ab
wd
fly
jump
capture
Mantid location
groom
walk
run
stop
retreat
occl
0
184
368
552
736
920
Time (s)

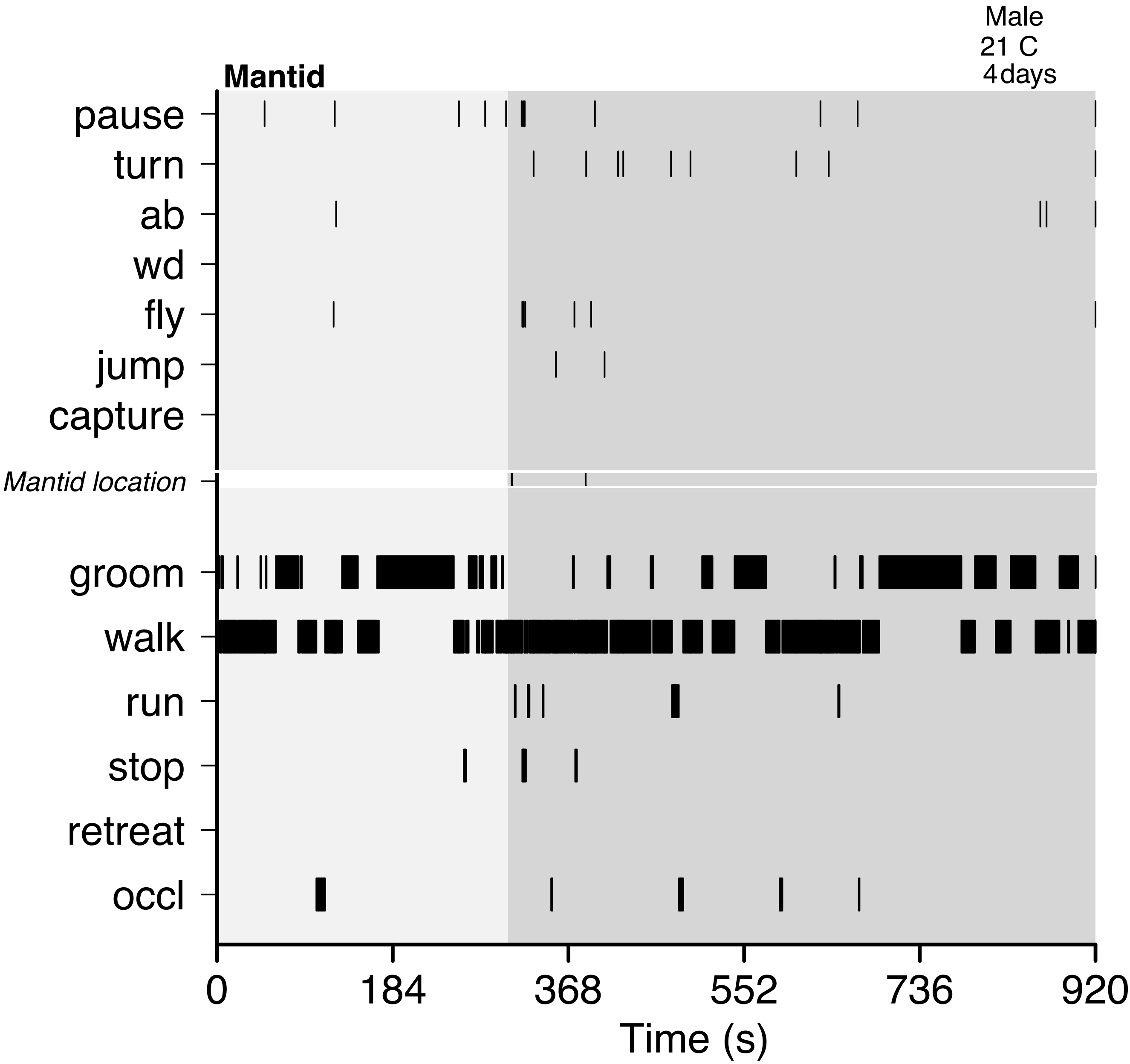

Male
21 C
4days
Mantid
pause
turn
ab
wd
fly
jump
capture
Mantid location
groom
walk
run
stop
retreat
occl
0
184
368
552
736
920
Time (s)

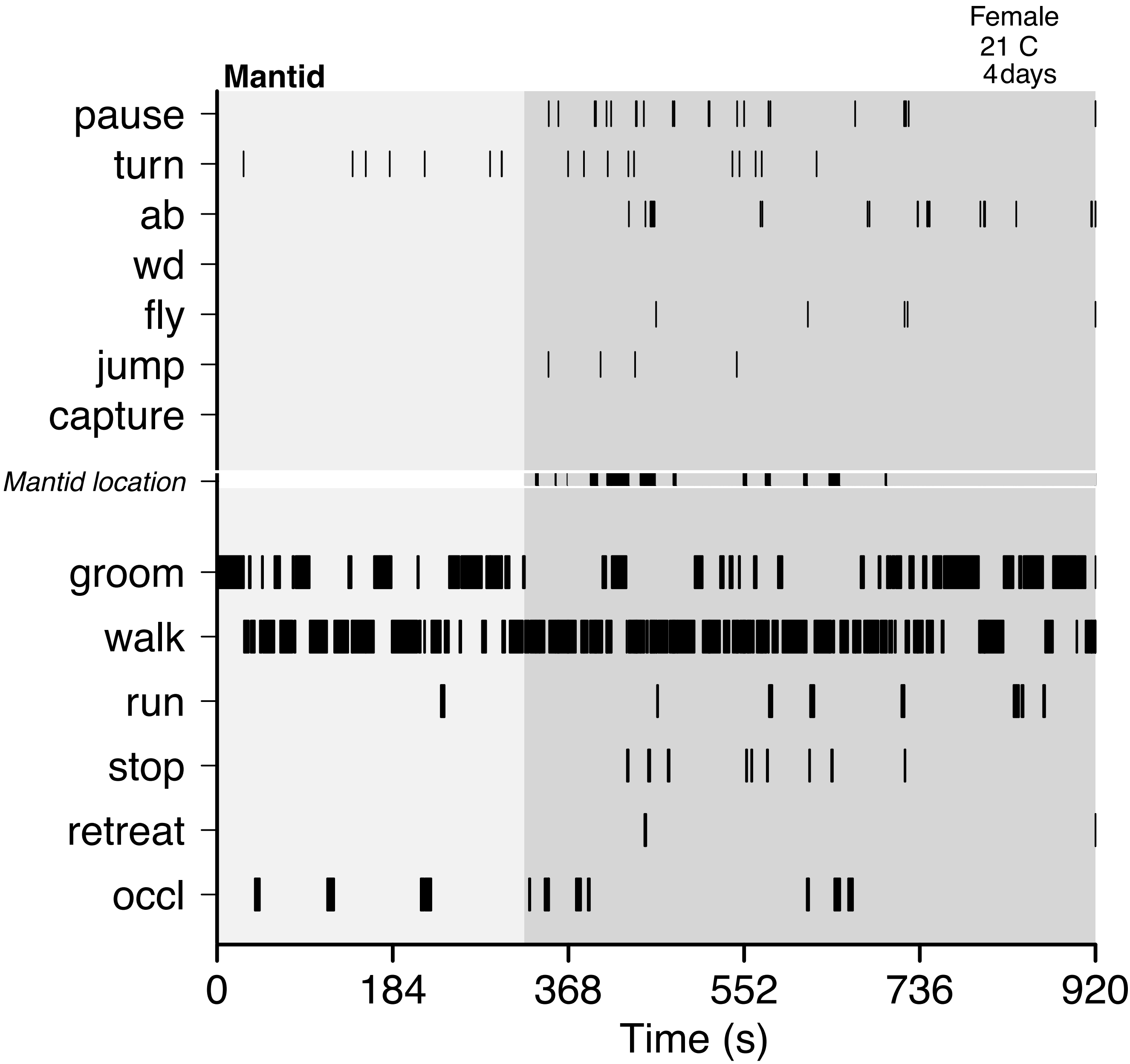
Female
21 C
4days
Mantid
pause
turn
ab
wd
fly
jump
capture
Mantid location
groom
walk
run
stop
retreat
occl
0
184
368
552
736
920
Time (s)

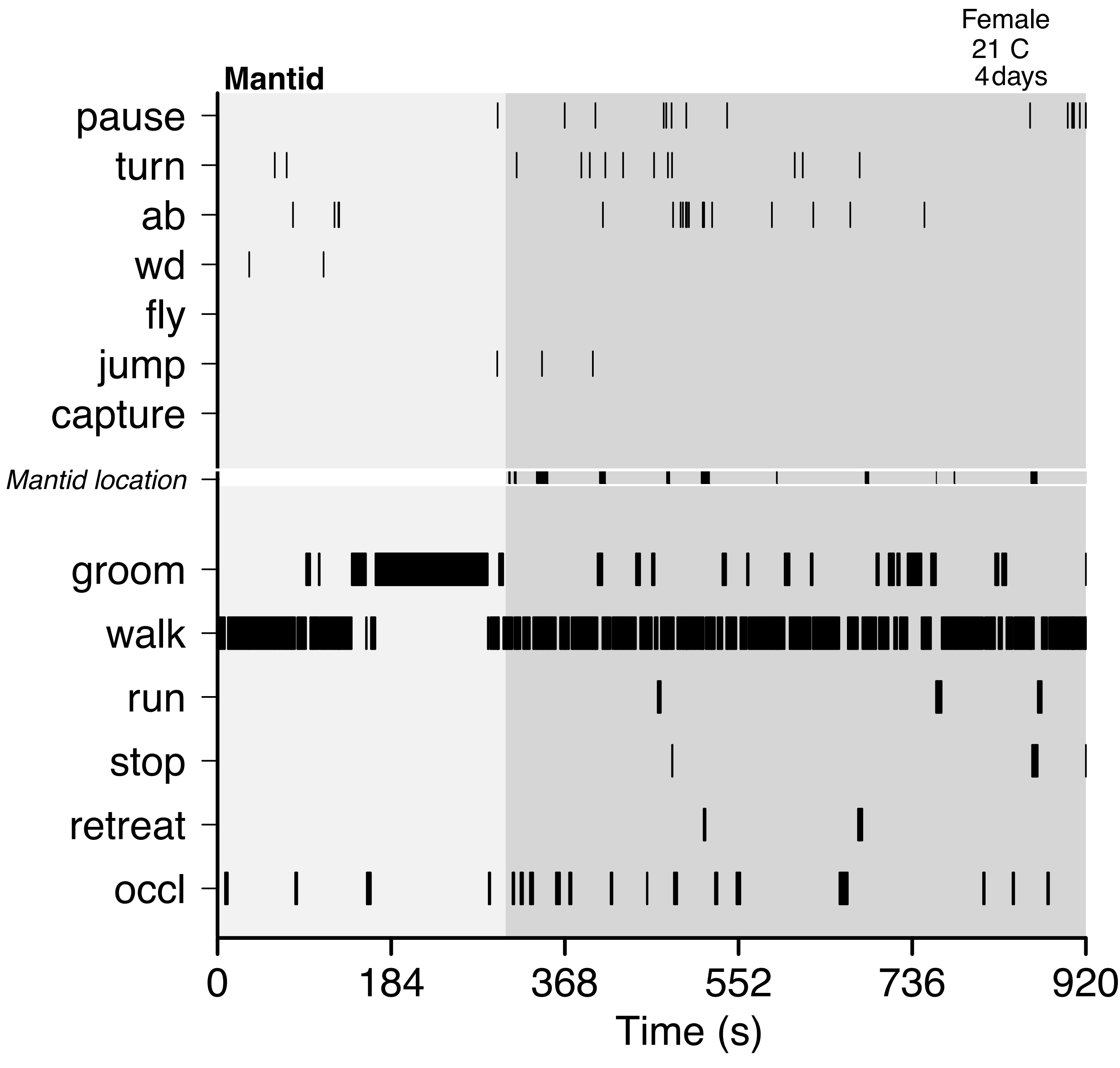
Female
21 C
4days
Mantid
pause
turn
ab
wd
fly
jump
capture
Mantid location
groom
walk
run
stop
retreat
occl
0
184
368
552
736
920
Time (s)

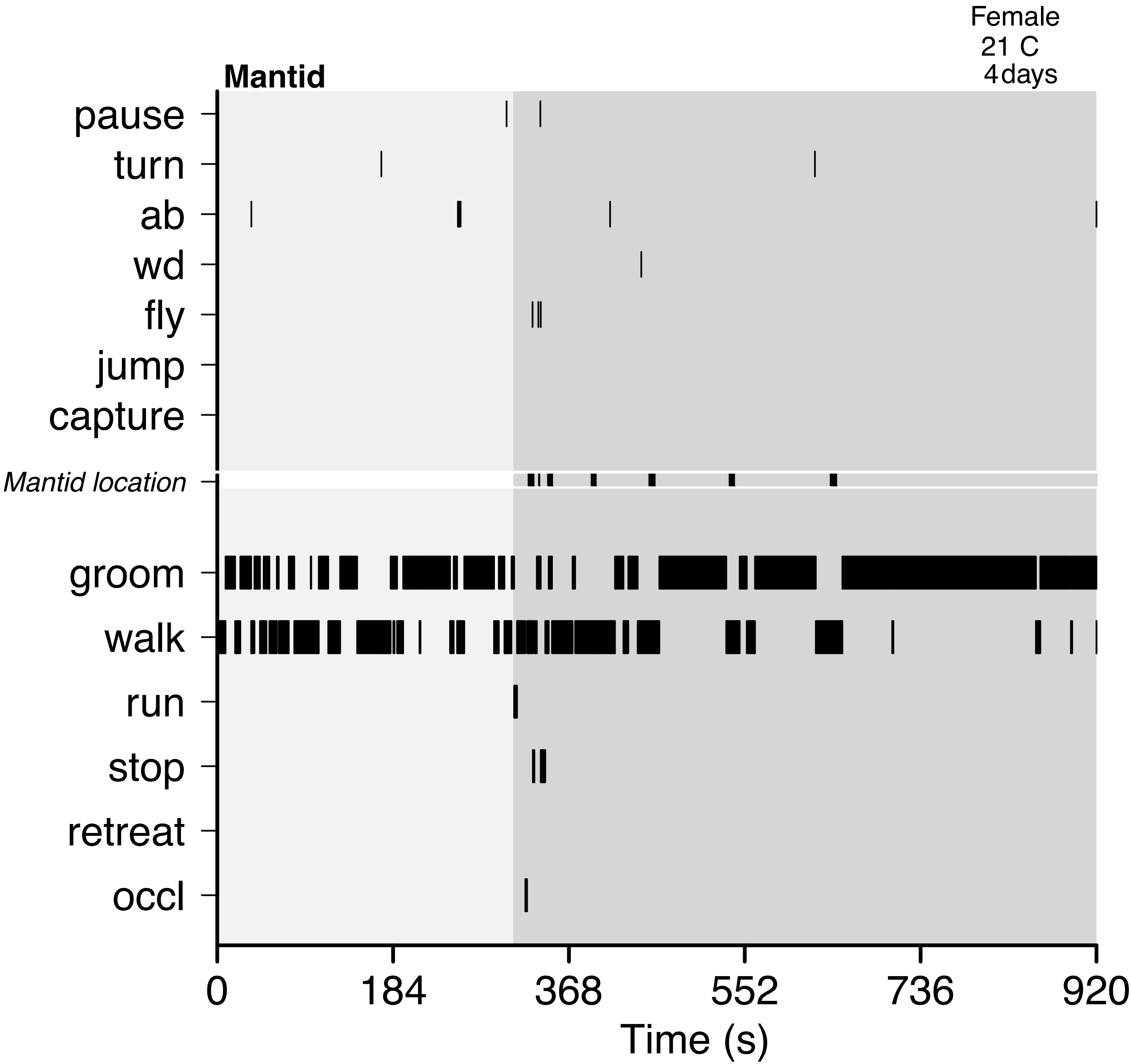

Female
21 C
4 days
Mantid
pause
turn
ab
wd
fly
jump
capture
Mantid location
groom
walk
run
stop
retreat
occl
0
184
368
552
736
920
Time (s)

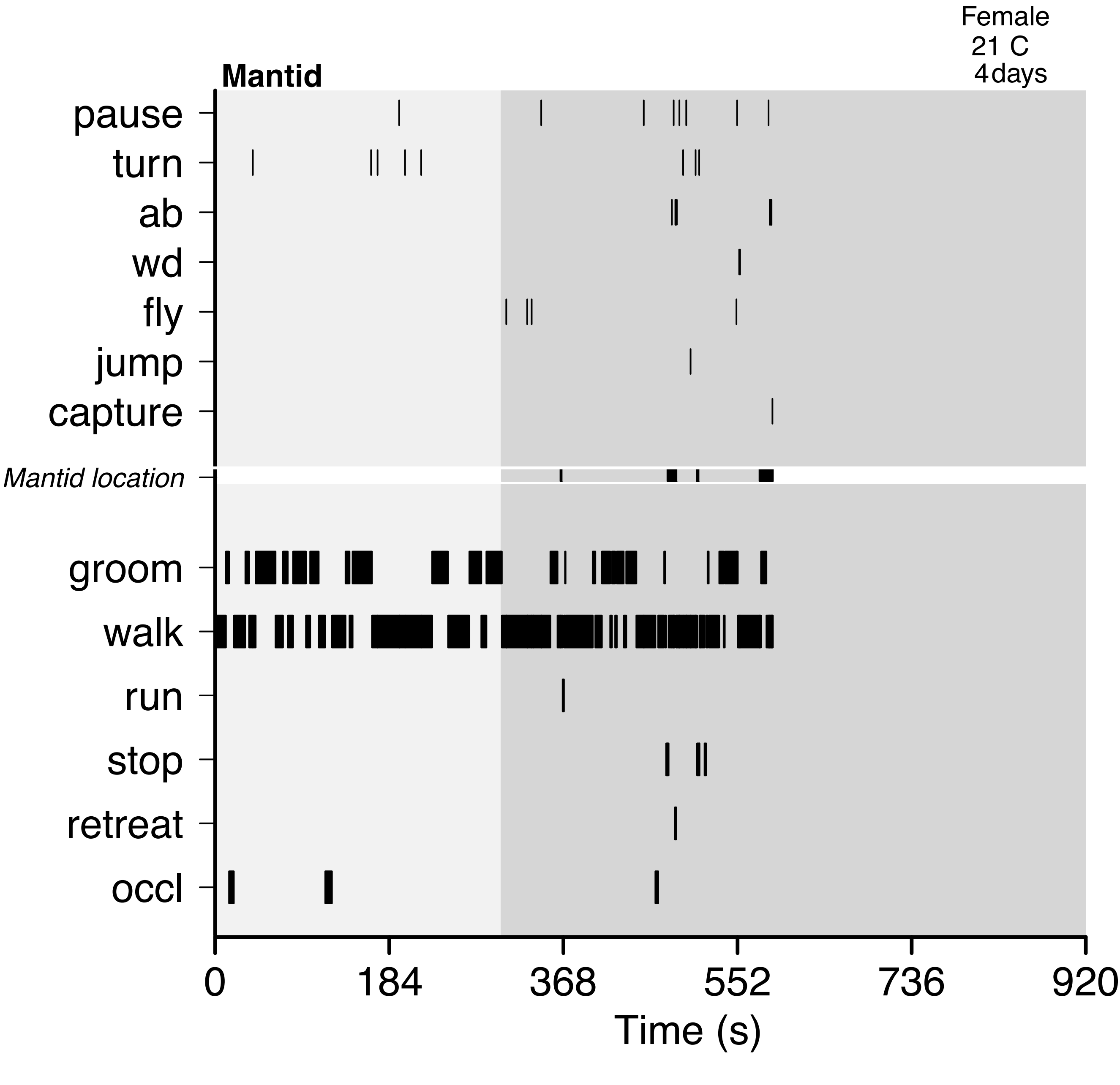

Female
21 C
4days
Mantid
pause
turn
ab
wd
fly
jump
capture
Mantid location
groom
walk
run
stop
retreat
occl
0
184
368
552
736
920
Time (s)

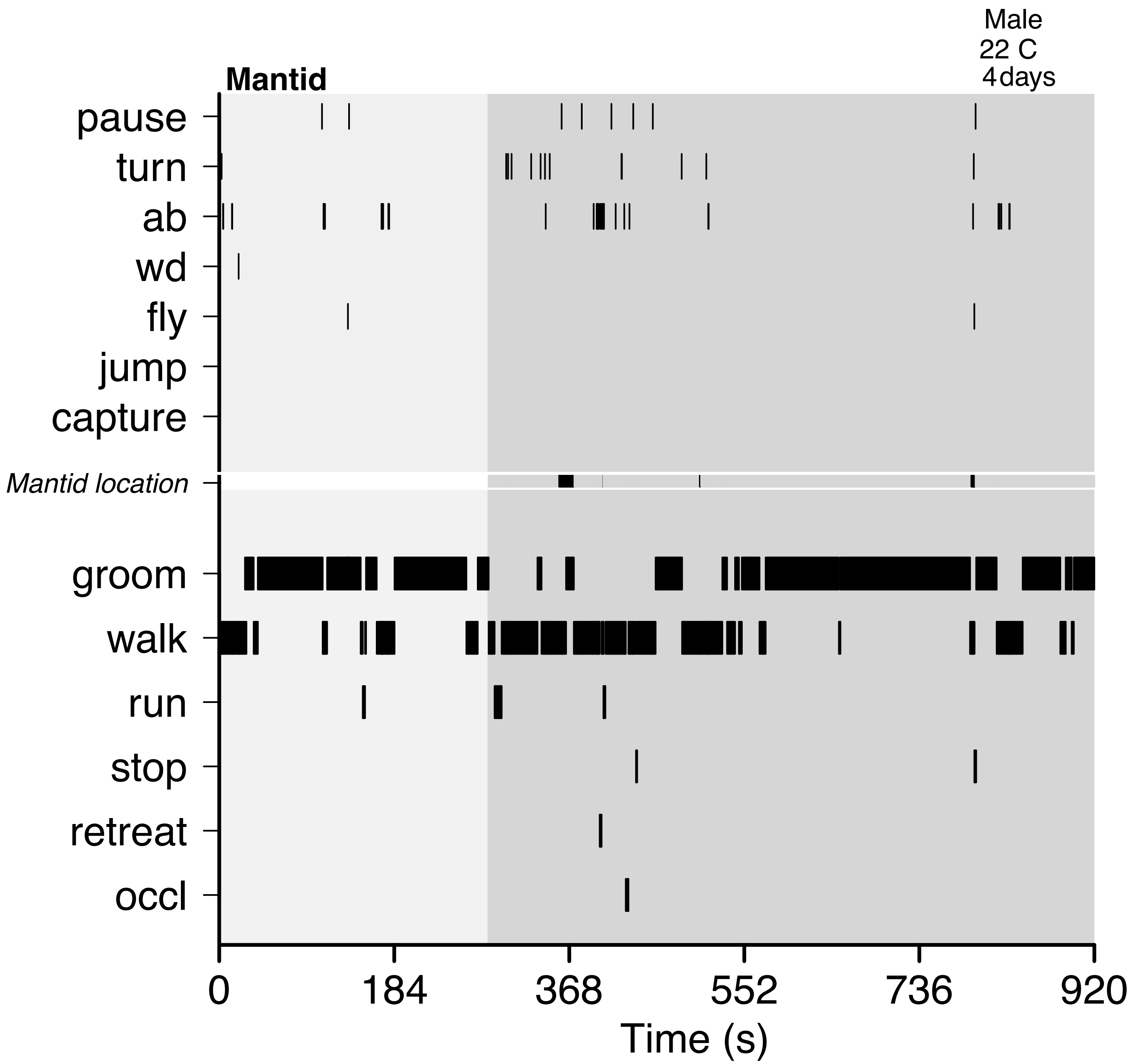

Male
22 C
4 days
Mantid
pause
turn
ab
wd
fly
jump
capture
Mantid location
groom
walk
run
stop
retreat
occl
0
184
368
552
736
920
Time (s)

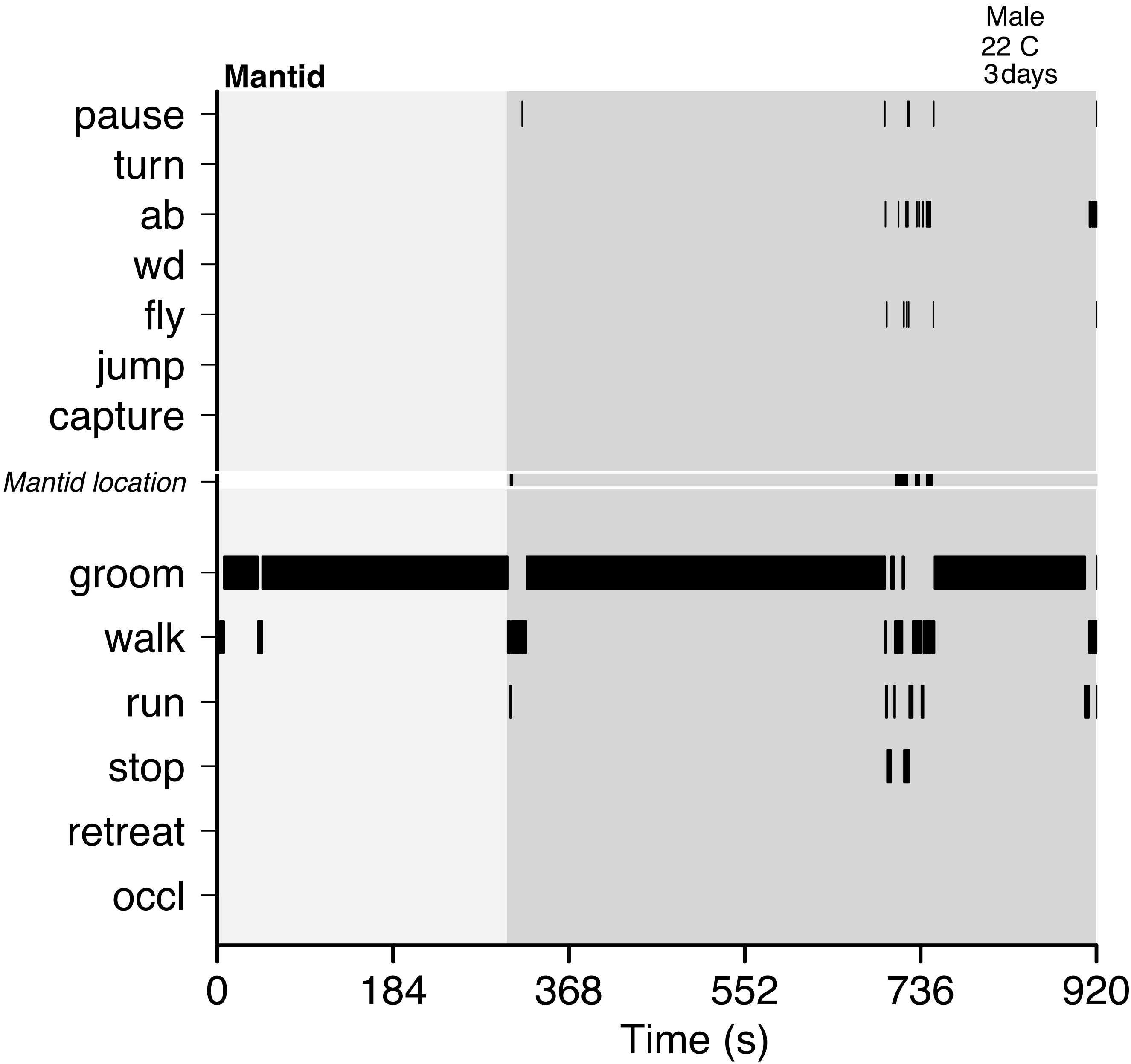

Mantid
Male
22 C
3days
pause
turn
ab
wd
fly
jump
capture
Mantid location
groom
walk
run
stop
retreat
occl
0
184
368
552
736
920
Time (s)

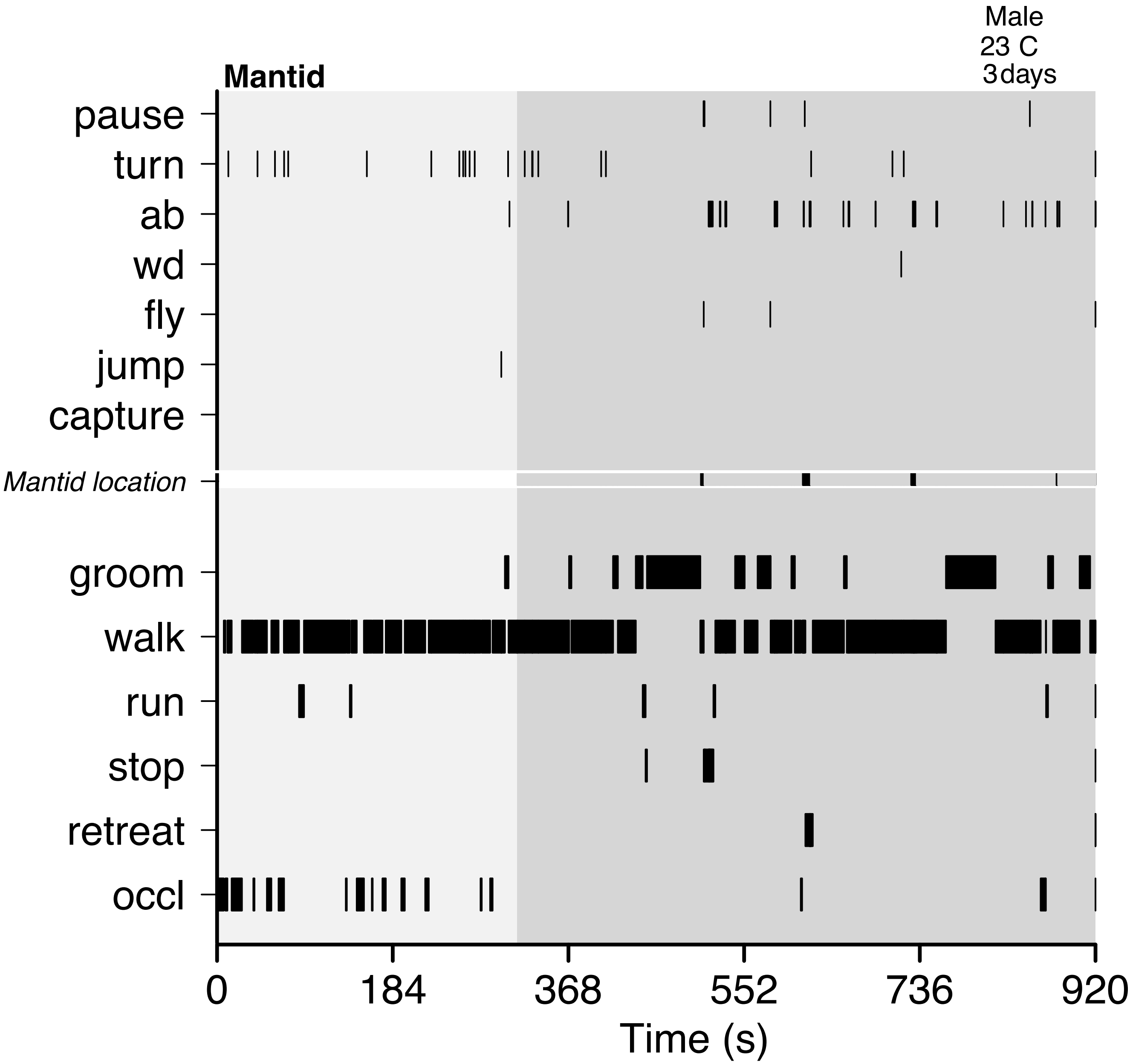

Male
23 C
3days
Mantid
pause
turn
ab
wd
fly
jump
capture
Mantid location
groom
walk
run
stop
retreat
occl
0
184
368
552
736
920
Time (s)

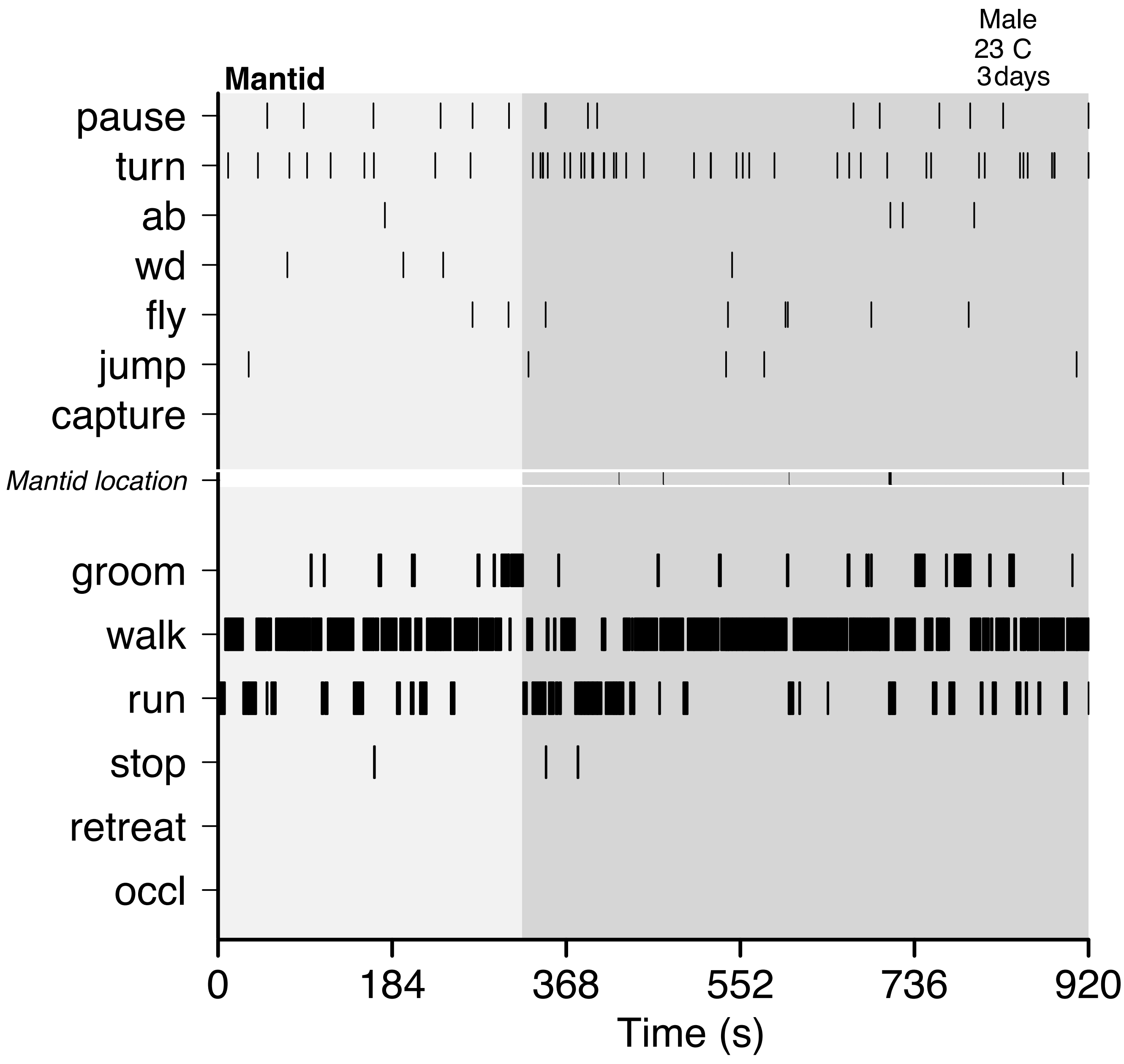
Male
23 C
3days
Mantid
pause
turn
ab
wd
fly
jump
capture
Mantid location
groom
walk
run
stop
retreat
occl
0
184
368
552
736
920
Time (s)

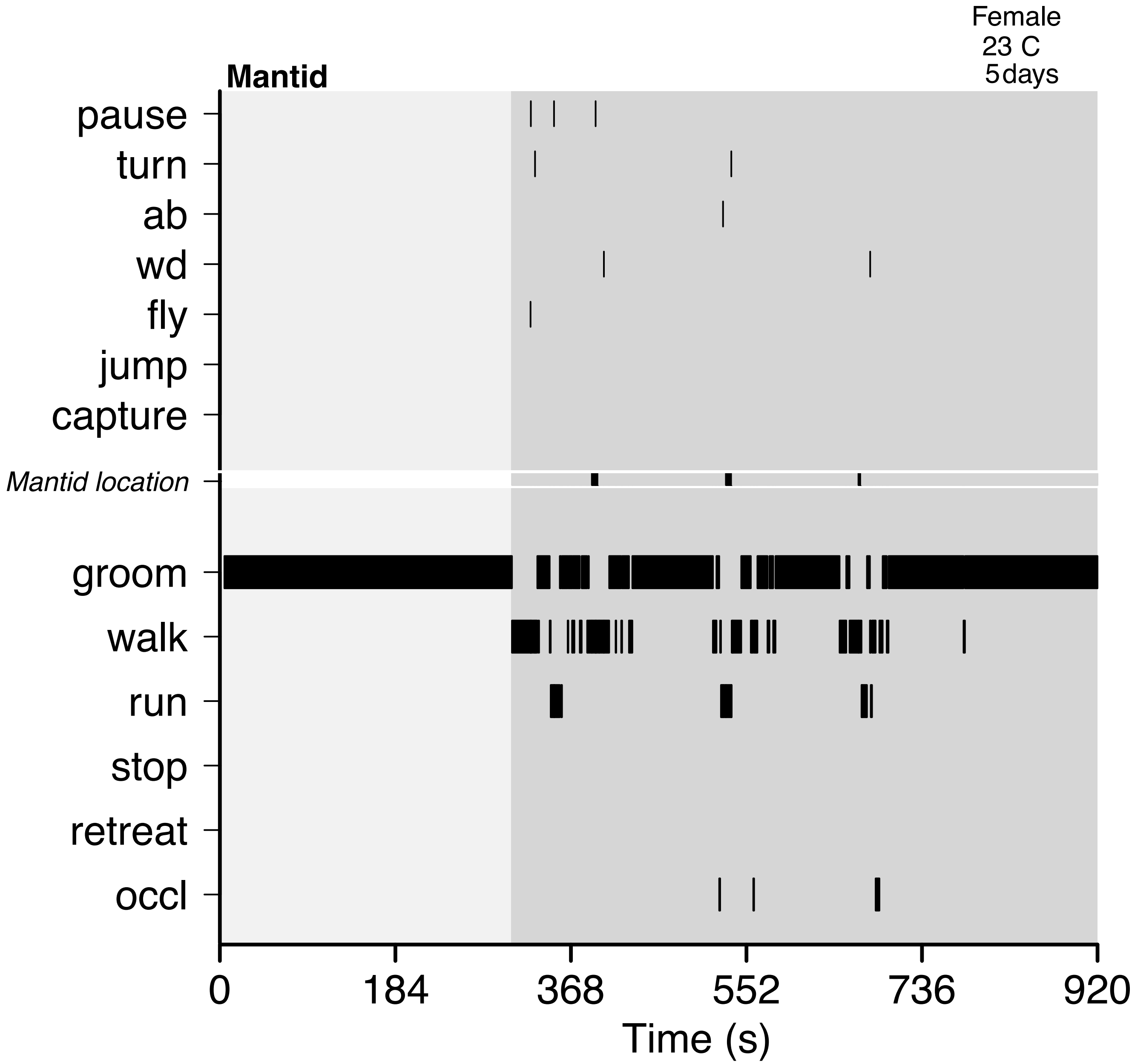

Female
23 C
5 days
Mantid
pause
turn
ab
wd
fly
jump
capture
Mantid location
groom
walk
run
stop
retreat
occl
0
184
368
552
736
920
Time (s)

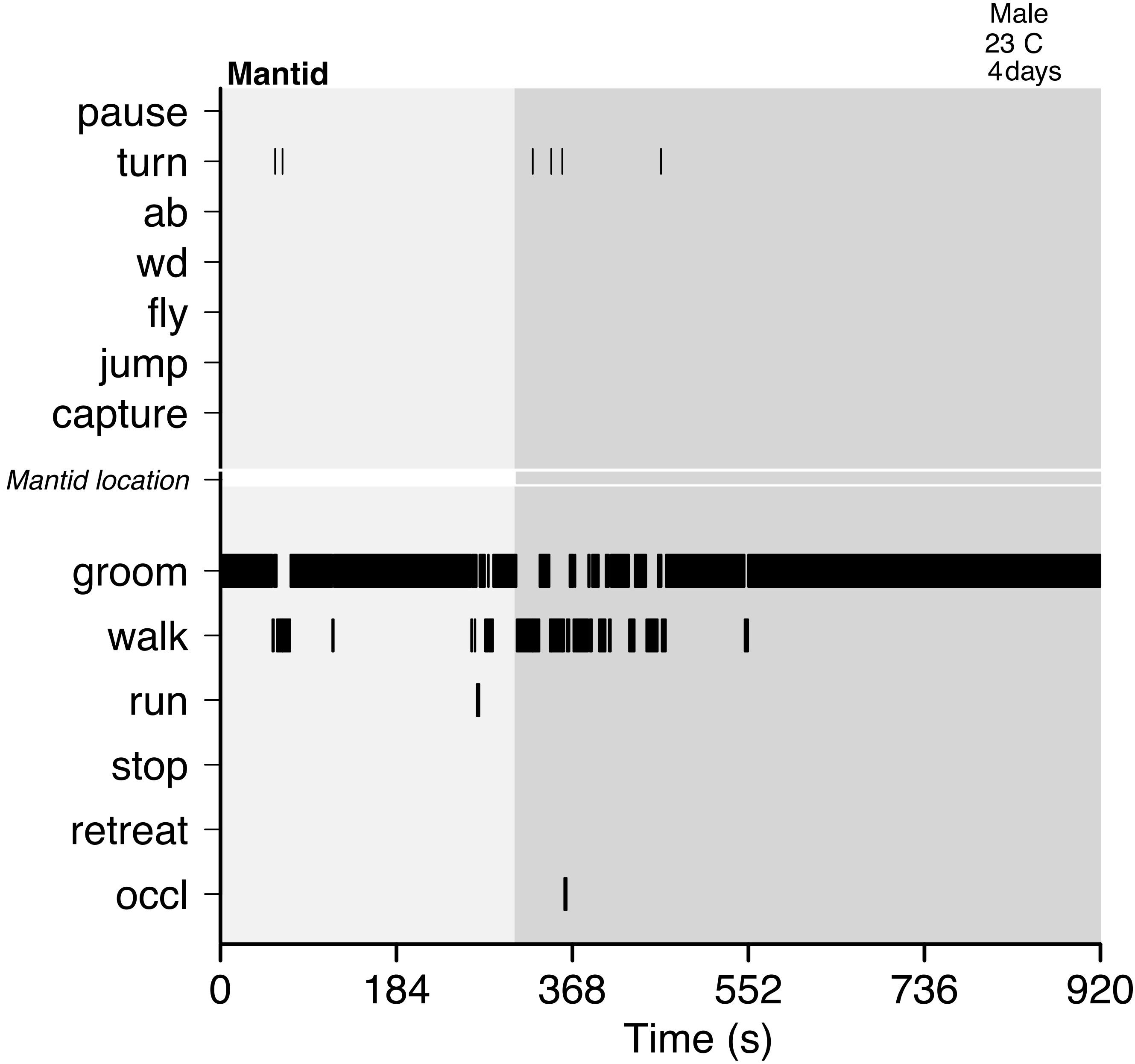

Male
23 C
4 days
Mantid
pause
turn
ab
wd
fly
jump
capture
Mantid location
groom
walk
run
stop
retreat
occl
0
184
368
552
736
920
Time (s)

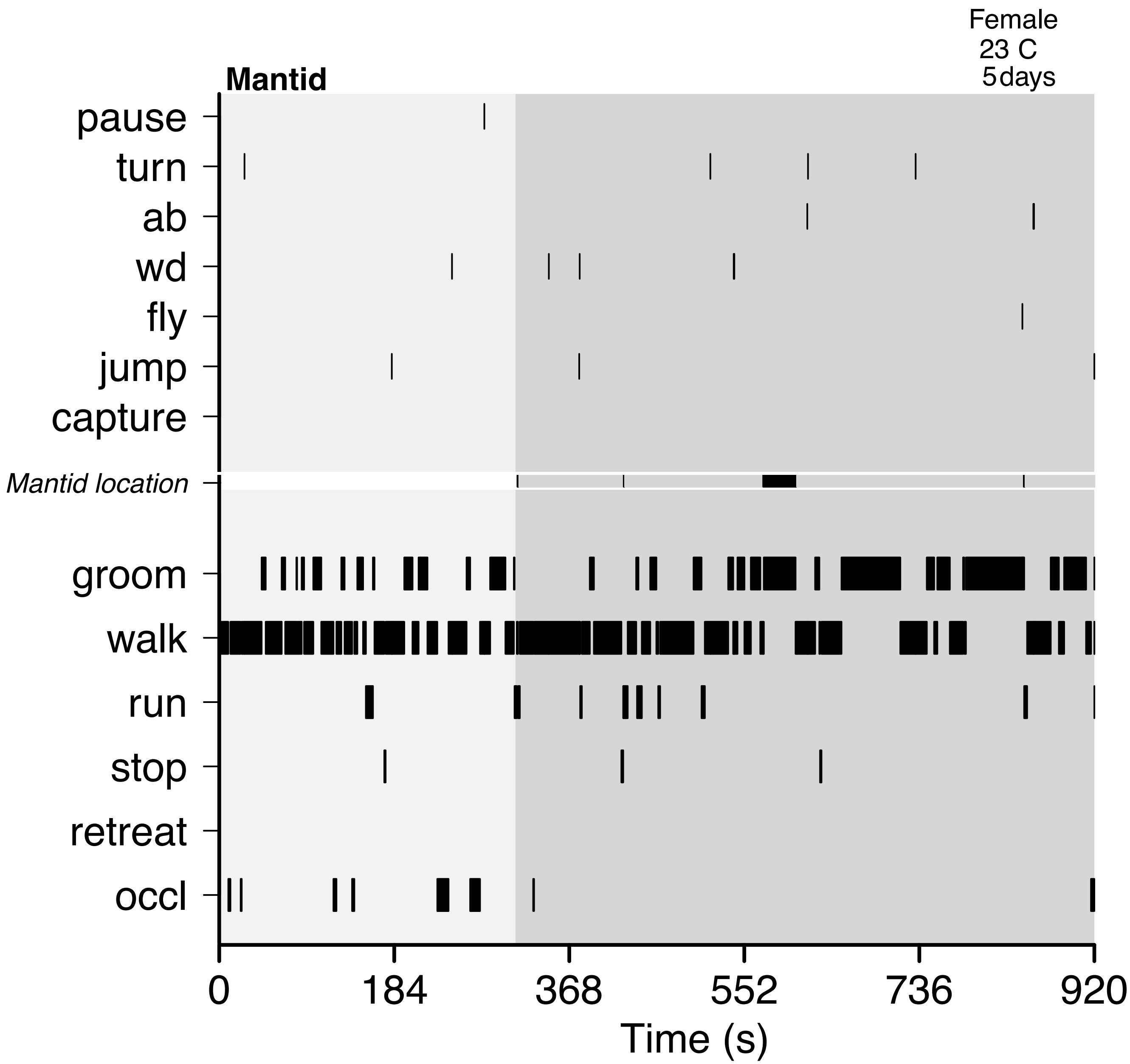

Female
23 C
5 days
Mantid
pause
turn
ab
wd
fly
jump
capture
Mantid location
groom
walk
run
stop
retreat
occl
0
184
368
552
736
920
Time (s)

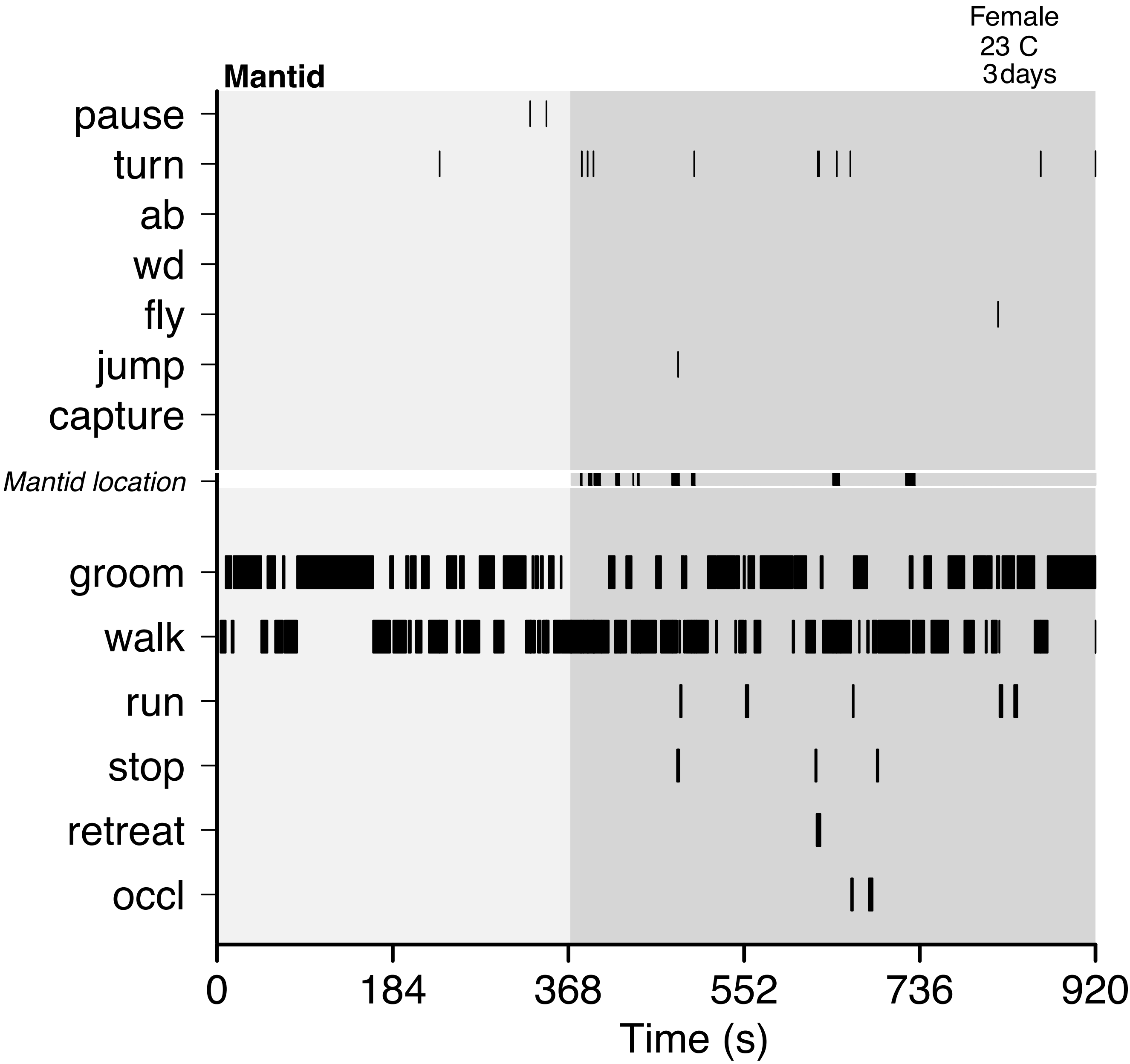

Female
23 C
3days
Mantid
pause
turn
ab
wd
fly
jump
capture
Mantid location
groom
walk
run
stop
retreat
occl
0
184
368
552
736
920
Time (s)

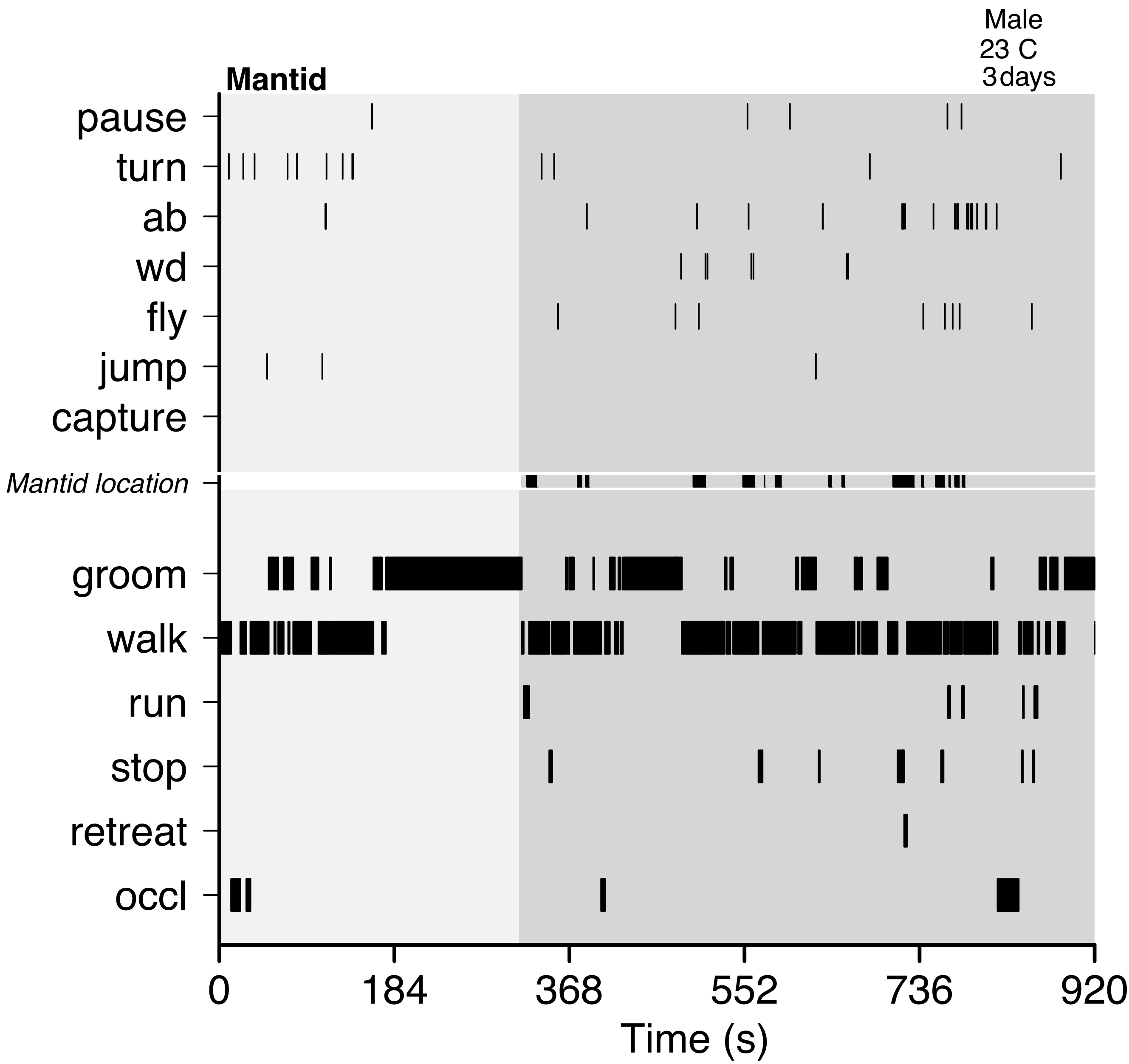
Male
23 C
3days
Mantid
pause
turn
ab
wd
fly
jump
capture
Mantid location
groom
walk
run
stop
retreat
occl
0
184
368
552
736
920
Time (s)

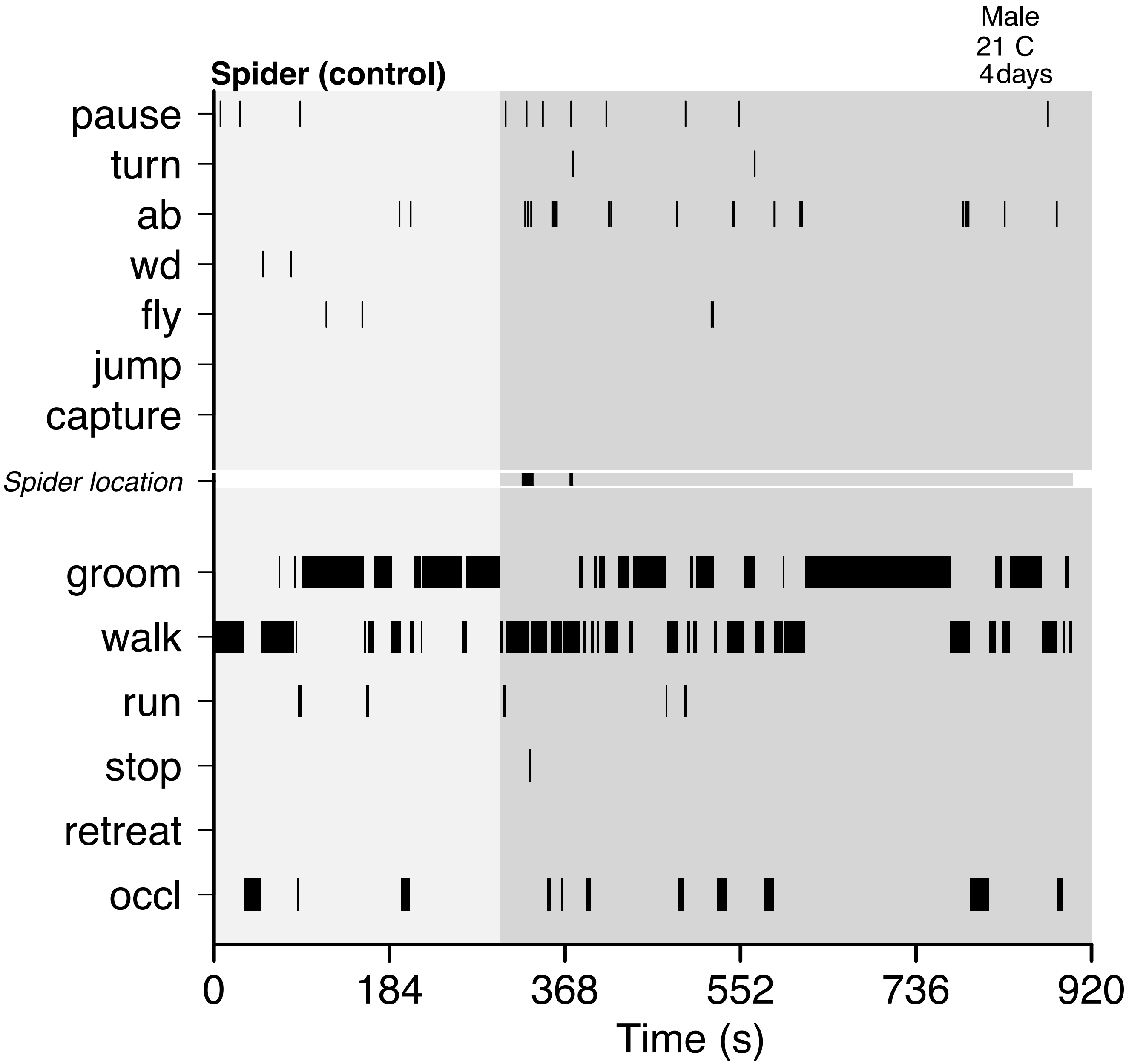

Male
21 C
4 days
Spider (control)
pause
turn
ab
wd
fly
jump
capture
Spider location
groom
walk
run
stop
retreat
occl
0
184
368
552
736
920
Time (s)

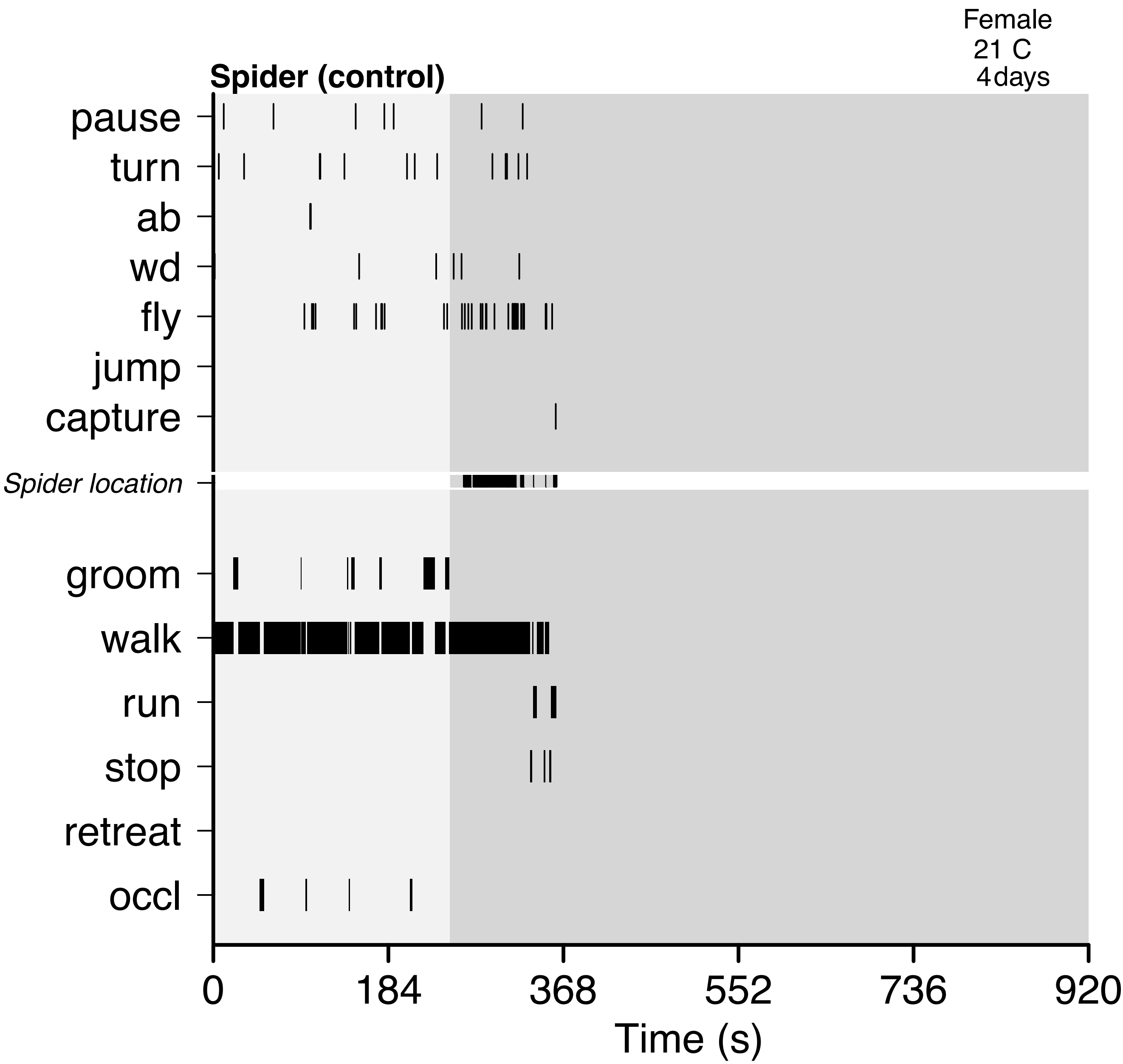

Female
21 C
4 days
Spider (control)
pause
turn
ab
wd
fly
jump
capture
Spider location
groom
walk
run
stop
retreat
occl
0
184
368
552
736
920
Time (s)

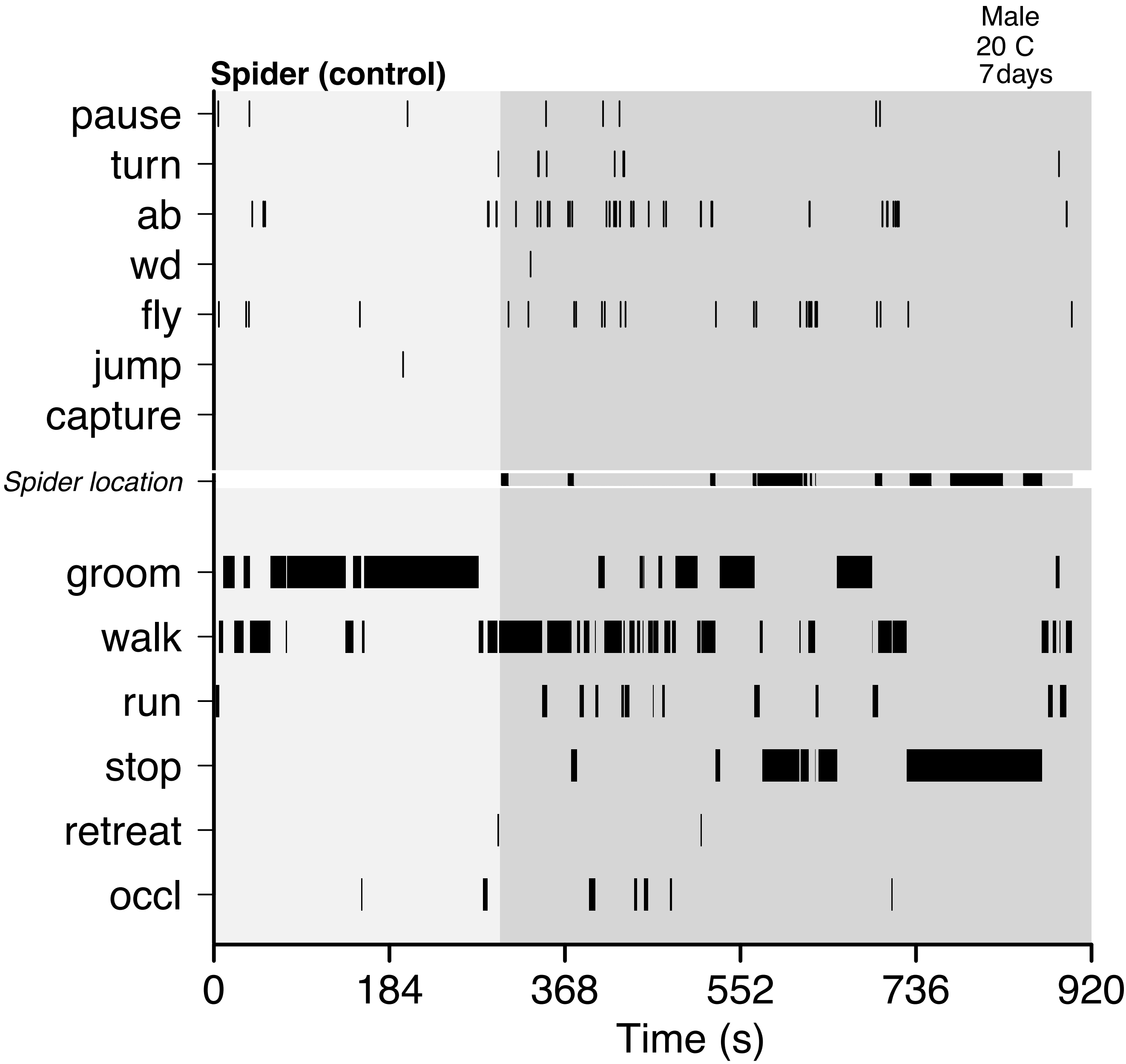
Male
20 C
7days
Spider (control)
pause
turn
ab
wd
fly
jump
capture
Spider location
groom
walk
run
stop
retreat
occl
0
184
368
552
736
920
Time (s)

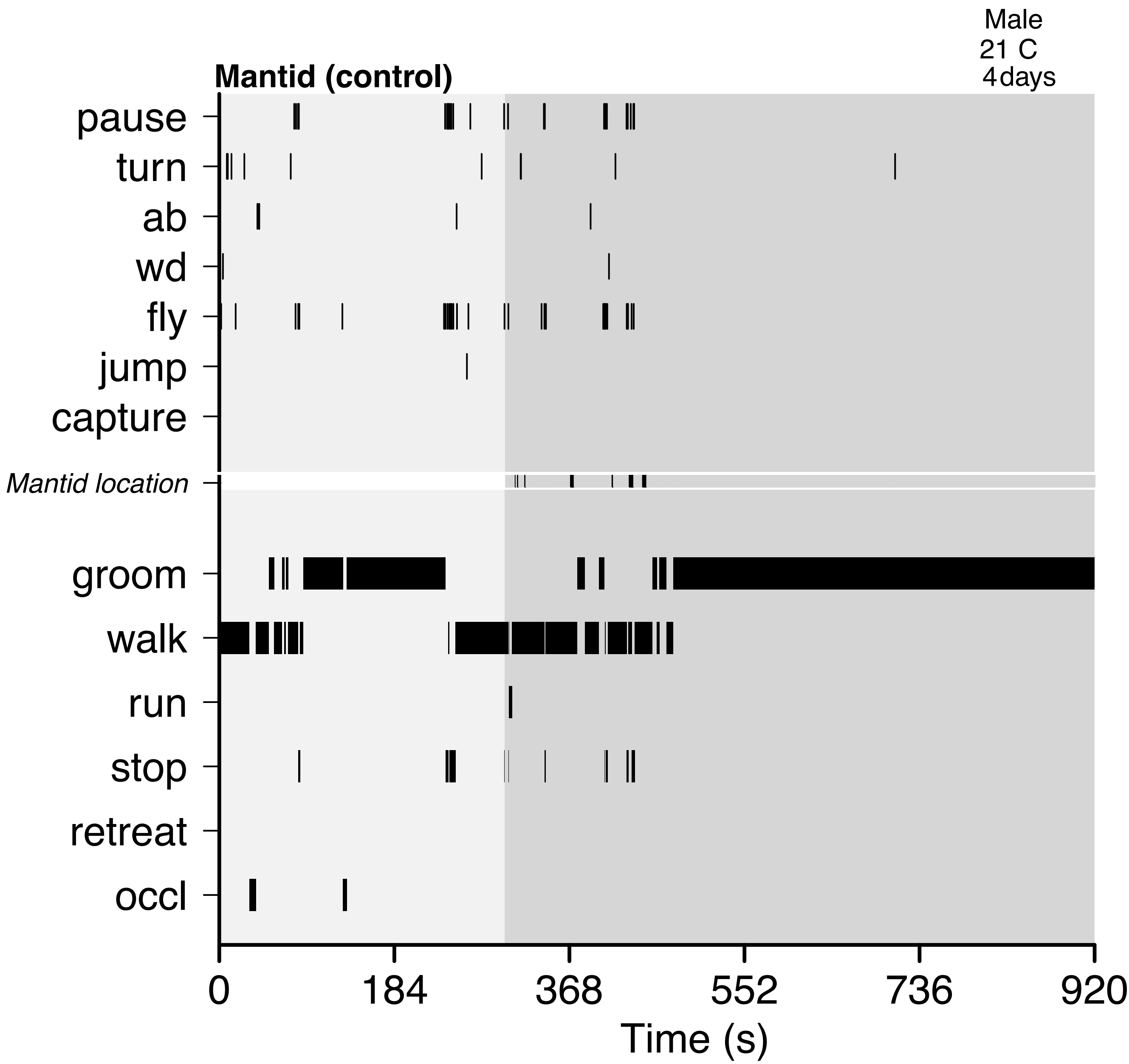
Male
21 C
4 days
Mantid (control)
pause
turn
ab
wd
fly
jump
capture
Mantid location
groom
walk
run
stop
retreat
occl
0
184
368
552
736
920
Time (s)

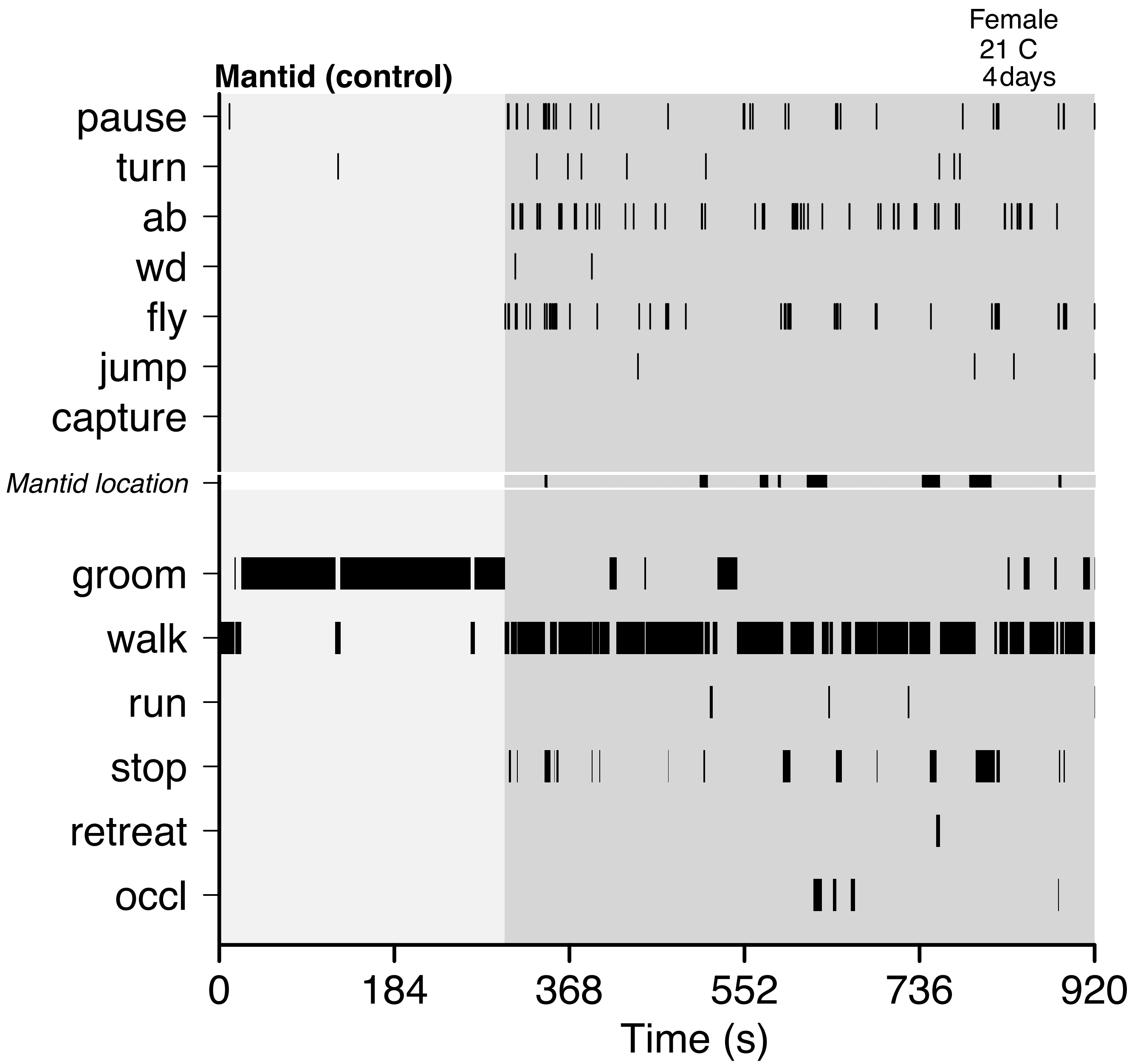

Female
21 C
4 days
Mantid (control)
pause
turn
ab
wd
fly
jump
capture
Mantid location
groom
walk
run
stop
retreat
occl
0
184
368
552
736
920
Time (s)

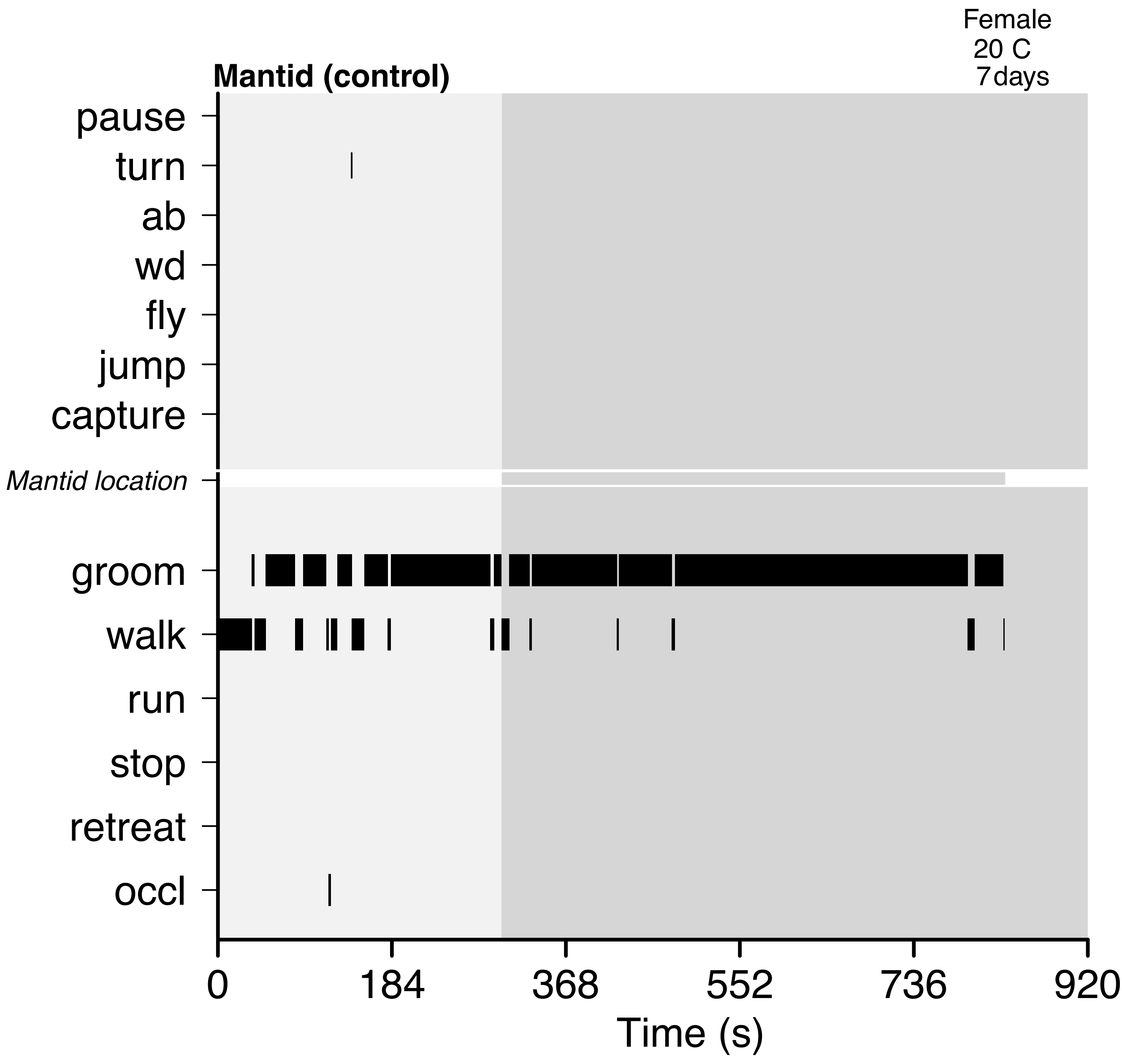
Female
20 C
7days
Mantid (control)
pause
turn
ab
wd
fly
jump
capture
Mantid location
groom
walk
run
stop
retreat
occl
0
184
368
552
736
920
Time (s)
