# Supplementary material for: The behavioral repertoire of Drosophila melanogaster in the presence of two predator species that differ in hunting mode
Source: PLoS One. 2019 May 31;14(5):e0216860. doi: 10.1371/journal.pone.0216860 (PMC6544228; doi:10.1371/journal.pone.0216860)
Supplement: S2 File — (DOCX) [file pone.0216860.s002.docx]

**Supplemental material**

**Table of contents**

| **Supplemental material type** | **Page numbers** |
| --- | --- |
| Supporting information | 1-2 |
| Supplemental figures | 2-7 |
| Supplemental tables | 8-14 |
| Videos | 14 |

**Supporting information**

**Differential response to spiders versus mantids**

Because spider and mantid population densities vary by season, we had to temporally segregate the spider assays from the mantid assays. We conducted all spider observations between October and December 2012 and all the mantid observations between March and May 2013. Comparing time allocation and frequencies of occurrences in the predator absent state between the two predator treatments suggest that behavioral modifications were predator induced, and not due to seasonal effects (S5 and S6 Fig). Although the assays were carried out under highly controlled conditions, to confirm that predator species-specific behavioral differences were not confounded with seasonal differences in behavior, we performed 6 additional assays (alternating between spider and mantid treatments) within the span of one week. The control experiments show no evidence of confounding effects of season with *D. melanogaster*’s anti-predator behavioral repertoire (S9, S11 and S12 Table below). Ethograms are shown in Supplement a. Furthermore, to confirm that the disturbance we caused (to the assay chamber) during the addition of a predator did not confound behavioral responses to the predator, we did 3 “no predator” control assays. For these “no predator” controls, instead of adding a predator to the arena, we caused a mild disturbance (~ to intensity of disturbance caused while adding the predator) without actually adding any predator. We found that disturbance caused during predator addition was not responsible for observed behavioral modifications (Table S10 and S13). Finally, “no predator” controls also ruled our temporal differences in fruit fly activity levels (Table S10 and S13).

**Supplemental figures**

**Fig A**

**Presence of jumping spider causes fruit flies to walk more and groom less, whereas the presence of mantid causes weaker, more variable (and not significant) changes in fruit fly activity levels.** Here we show coefficient plots from the output of mixed effects models using the package MCMCglmm to visualize duration of two behaviors (**grooming in black** and **locomotion in red**) as a function of predator state (present vs. absent of spiders, left panels and mantids, right panels), total time spent in the assay [Time (cent.)], sex of the fly, start time of the assay, temperature in the room and age of the fly. The continuous covariate “Time” was centered around the mean and therefore reflects the average increase in time spent per minute of the assay. Estimates are in seconds. Error bars are ± 95% CI.

**Fig B**

**Fruit flies “stop” significantly longer in the presence of spiders (left panel), and to a much lesser extent (and not significantly) in the presence of mantids (right panel).** Here we have coefficient plots made from the output of mixed effects models using the package MCMCglmm to visualize duration of “stopping” as a function of predator state (present vs absent), time spent in the assay [Time (cent.)], sex of the fly, start time of the assay, temperature in the room and age of the fly. Estimates are in seconds. Error bars are ± 95% CI. The continuous covariate “Time” was centered around the mean and therefore reflects the average increase in time spent per minute of the assay. Although assays were performed between 9 am and 12 pm each day, start time for the mantid assays significantly affected the total time that flies spent “stopping”.

**Fig C**

**In the presence of spiders, fruit flies increased the frequency with which they performed flights, pauses and jumps**. Here we show coefficient plots made from the output of mixed effects models where the events were modelled using a poisson generalized linear mixed model with a log-link function fit using the lmer function, and estimates remain on a natural log scale. Output of the mixed model using the package MCMCglmm was consistent with the output of lmer. Coefficient plots were used to visualize frequency of each individual behavioral event (ab, fly, pause, wd, turn and jump) as a function of predator state (present vs absent of a spider) and, in some cases, time spent by the fly in the assay [Time (cent.)]. All estimates are scaled to number of events per minute. Error bars are ± 95% CI.

**Fig D**

**Fruit flies performed abdominal lifts at a higher frequency in the presence of a juvenile mantid.** Coefficient plots made from the output of mixed effects models where the events were modelled using a poisson generalized linear mixed model with a log-link function fit using the lmer function, and estimates remain on a natural log scale. Coefficient of the model output are used to visualize frequency of each individual behavioral event (ab, fly, pause, wd, turn and jump) as a function of predator state (present vs. absent of a mantid) and, in some cases, time spent in the assay [Time (cent.)]. All estimates are scaled to number of events per minute. Error bars are ± 95% CI.

**Fig E**

**Hunting mode induced behavioral differences in fruit fly behaviors were not confounded with seasonal effects**. Here we show percentage time spent in each behavioral state (left) and number of occurrences per minute for each behavioral event (right) as measured for individual fruit flies before the addition of a spider (white circles) and before the introduction of a mantid (black circles) into the chamber. Error bars are ± 2 * SEs. Overlapping error bars suggest that there was minimal effect of season on the behavioral repertoire of fruit flies.

**Fig F**

**Seasonal differences in fruit fly behaviors did not confound behavioral differences induced by different predators.** Flies measured before the addition of a spider did not differ in behavior from flies measured before the addition of a mantid.

**Fig G**

**a) A diagram representing probability of transitioning from one fly behavior to the other when individuals were measured before the addition of a spider b) A diagram representing probability of transitioning from one fly behavior to the other for individuals measured before the addition of a juvenile mantid.** Thickness of arrows indicates transition probability between the two behaviors. The arrowhead points to the behavior being transitioned to. Thickness of the box around behavioral state (groom, run, occl, retreat, stop and walk) indicate the mean proportion of total time spent in that behavior, whereas thickness of the box around behavioral events (fly, jump, turn, wd, ab) indicates mean number of occurrences per minute of that behavior. To reduce the complexity of the web we combined the behaviors “pause” with the behavior “stop”. Behavioral transitions that occurred less than 10 times have not been shown in the figure.

******

**Fig H**

**Effects of predator state on the mean proportion of time fruit flies spend on different behaviors**. Here “predator state” is divided into three categories: “predator close” is when the predator is within striking distance of the fruit fly, “predator far” is when the predator is in the arena but too far away to perform a successful strike, and “no predator” refers to the control conditions in the absence of a predator. Error bars are ± 95% CI. Results shown here are made from raw data and do not reflect estimates derived from the mixed effects models described in the results section. Because fruit flies rarely moved into a mantid’s striking zone, sample sizes for “predator close” in the mantid treatments are very low and preclude further statistical analyses.

**Fig I**

**Effects of predator on the mean number of times a behavioral event is performed per minute.** Here “predator state” is divided into three categories: “predator close” is when the predator is within striking distance of the fruit fly, “predator far” is when the predator is in the arena but too far away to perform a successful strike, and “no predator” refers to the control conditions in the absence of a predator. Error bars are ± 95% CI. Results shown here are made from raw data and do not reflect estimates derived from the mixed effects models described in the results section. Because fruit flies rarely moved into a mantid’s striking zone, sample sizes for “predator close” in the mantid treatments are very low and preclude further statistical analyses.

**Supplemental tables**

**Table A.** **Transition frequency matrix when a spider was present in the chamber. Each row represents the number of times one behavior (row name) transitioned to another behavior (column name)**. Numbers in blue represent transitions that occurred more often that expected under a model of independence, whereas numbers in red are transitions that occurred less often than expected (see methods).


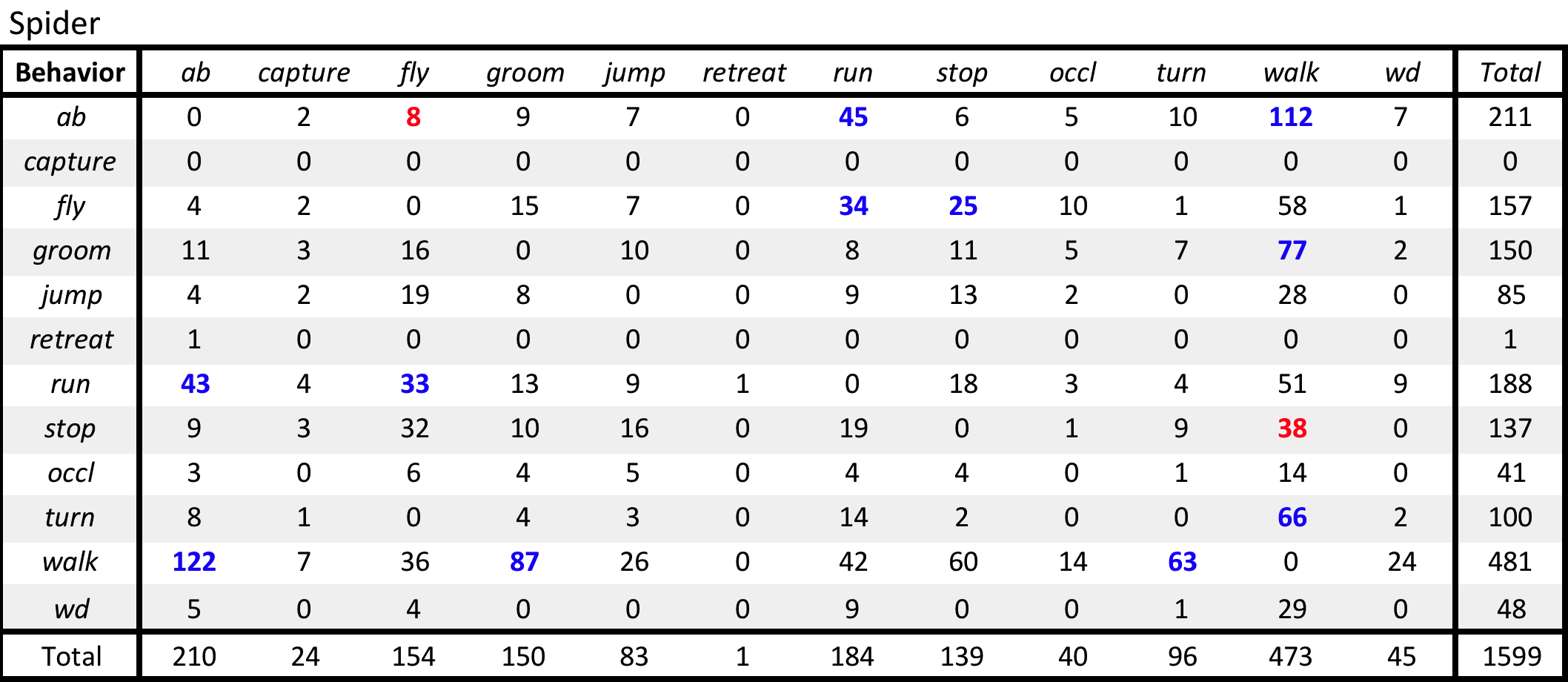


**Table B.** **Transition frequency matrix before a spider was added to the chamber. Each row represents the number of times one behavior (row name) transitioned to another behavior (column name)**. Numbers in blue represent transitions that occurred more often than expected under a model of independence, whereas numbers in red are transitions that occurred less often than expected (see methods).


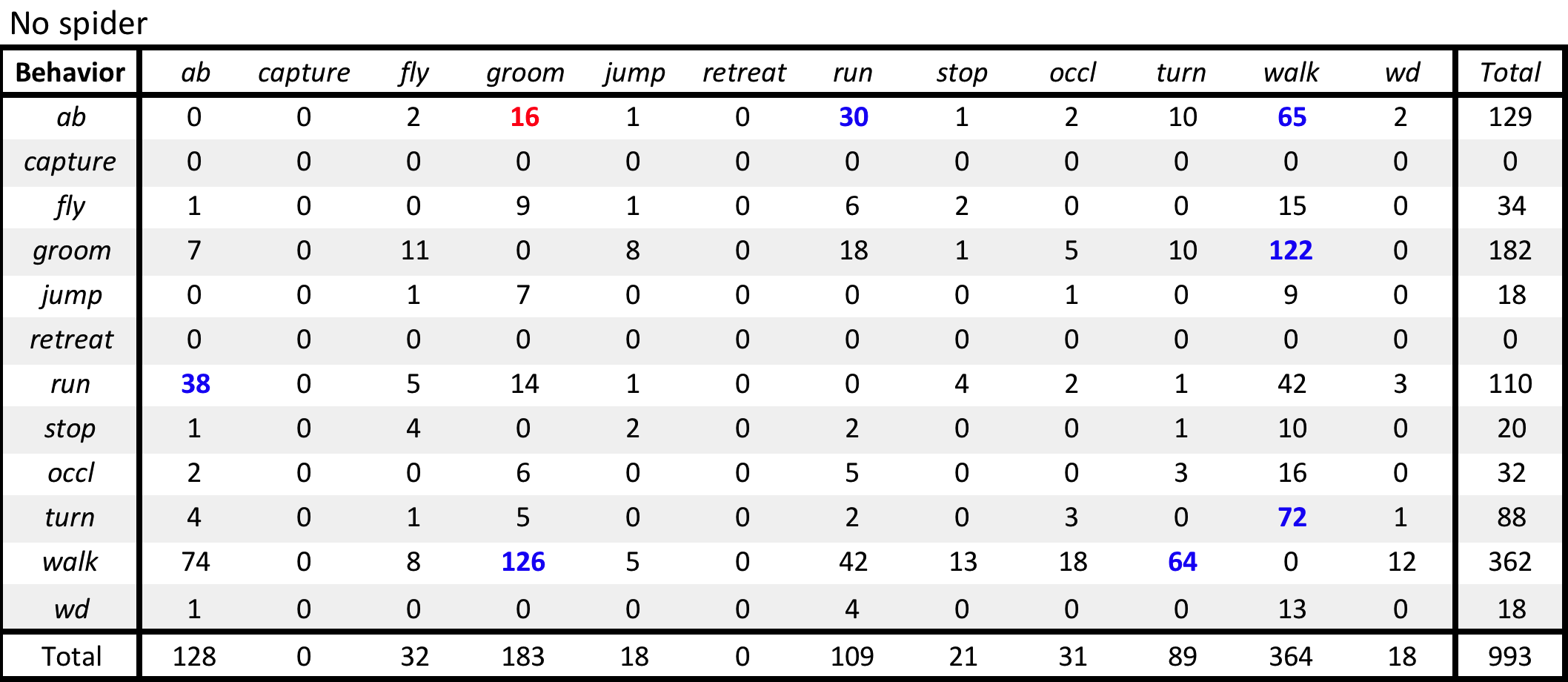


**Table C. Transition frequency in the presence of a juvenile praying mantid. Each row represents the number of times one behavior (row name) transitioned to another behavior (column name).** Numbers in blue represent transitions that occurred more often than expected whereas numbers in red are transitions that occurred less often than expected.


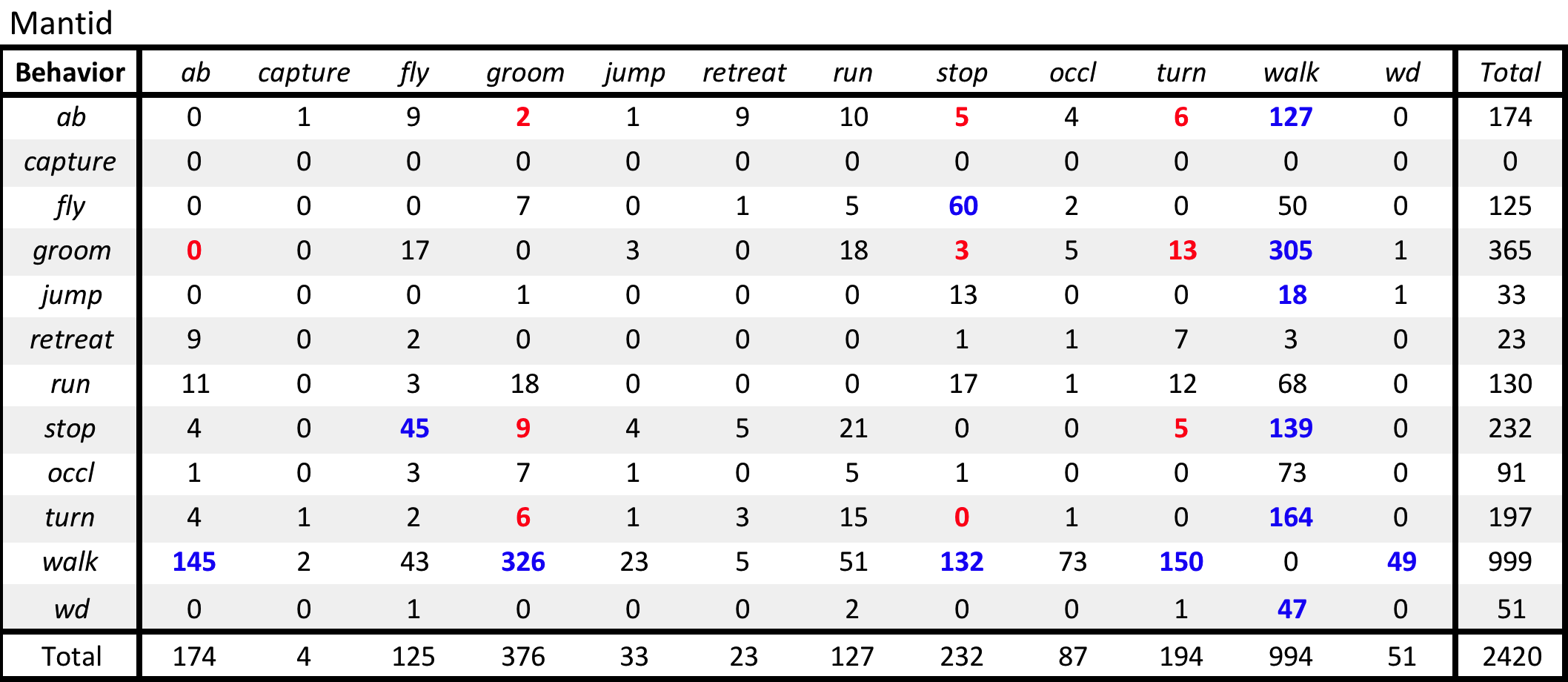


**Table D.** **Transition frequency matrix before a juvenile mantid was added to the chamber.** Each row represents the number of times one behavior (row name) transitioned to another behavior (column name). Numbers in blue represent transitions that occurred more often than expected whereas numbers in red are transitions that occurred less often than expected.


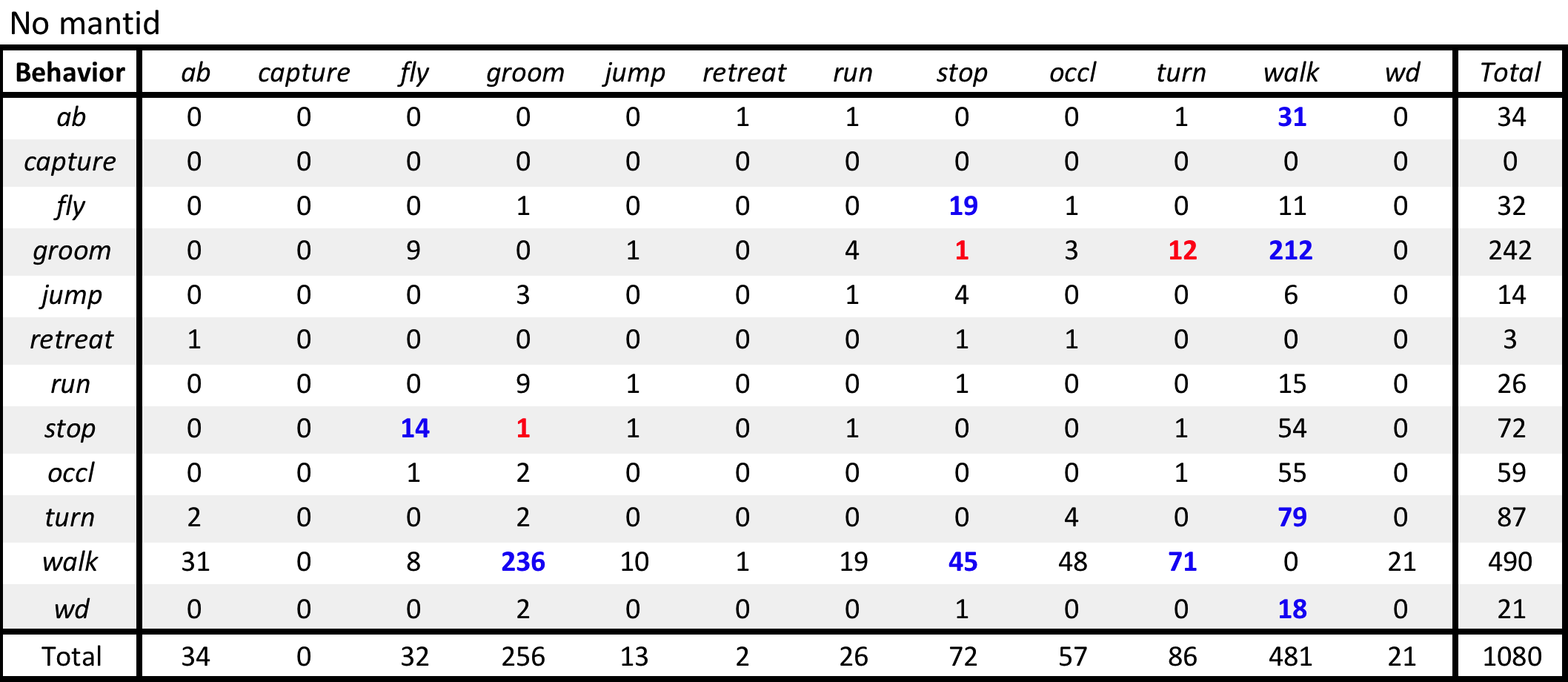


**Table E. Transition probability from one behavior (row name) to the other (column name) in the presence of a zebra jumping spider**. Transition probabilities are obtained by dividing each transition frequency (see table S1) between a pair of behaviors by the total number of times a given behavior was performed (row sums in table S1).


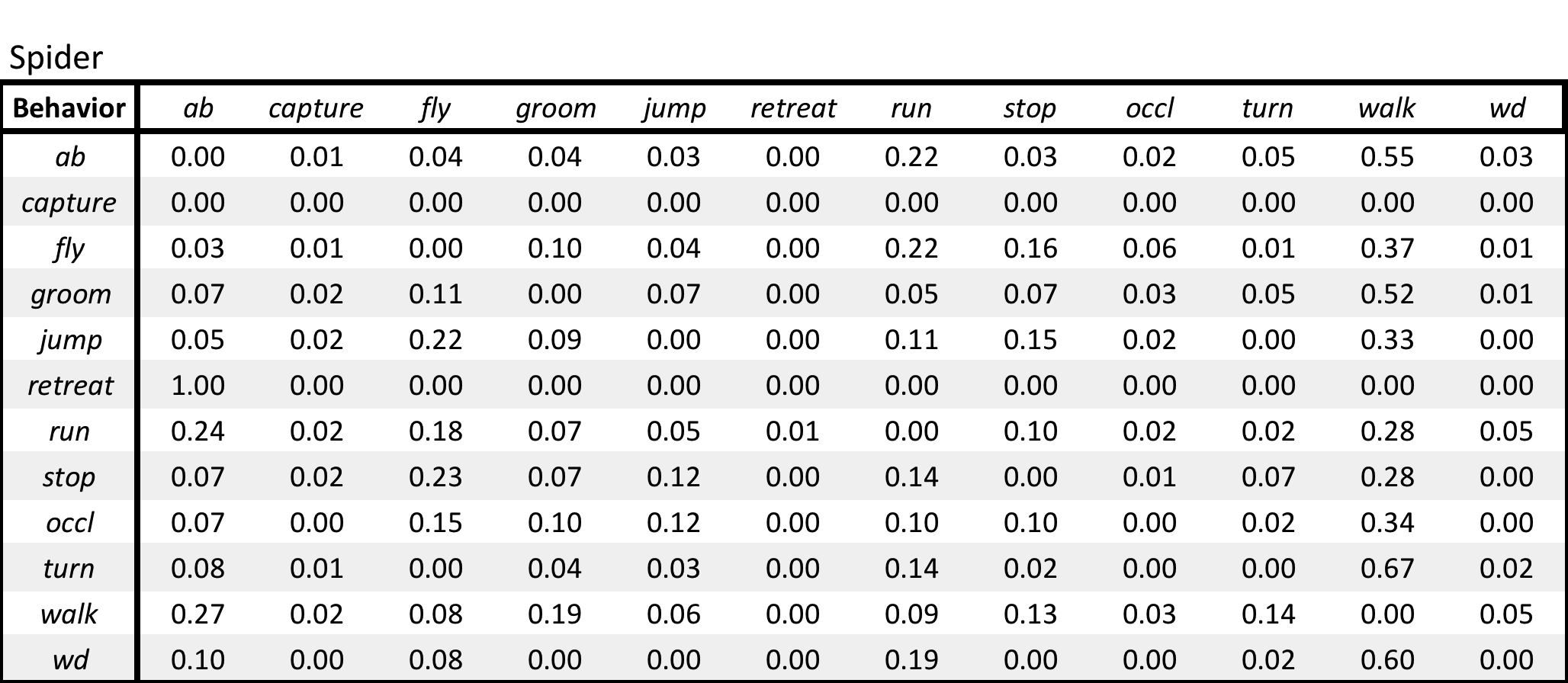


**Table F.** **Transition probability from one behavior (row name) to the other (column name) before a zebra jumping spider was introduced into the arena.** Transition probabilities are obtained by dividing each transition frequency (see S1 Table) between a pair of behaviors by the total number of times a given behavior was performed (row sums in S1 Table).


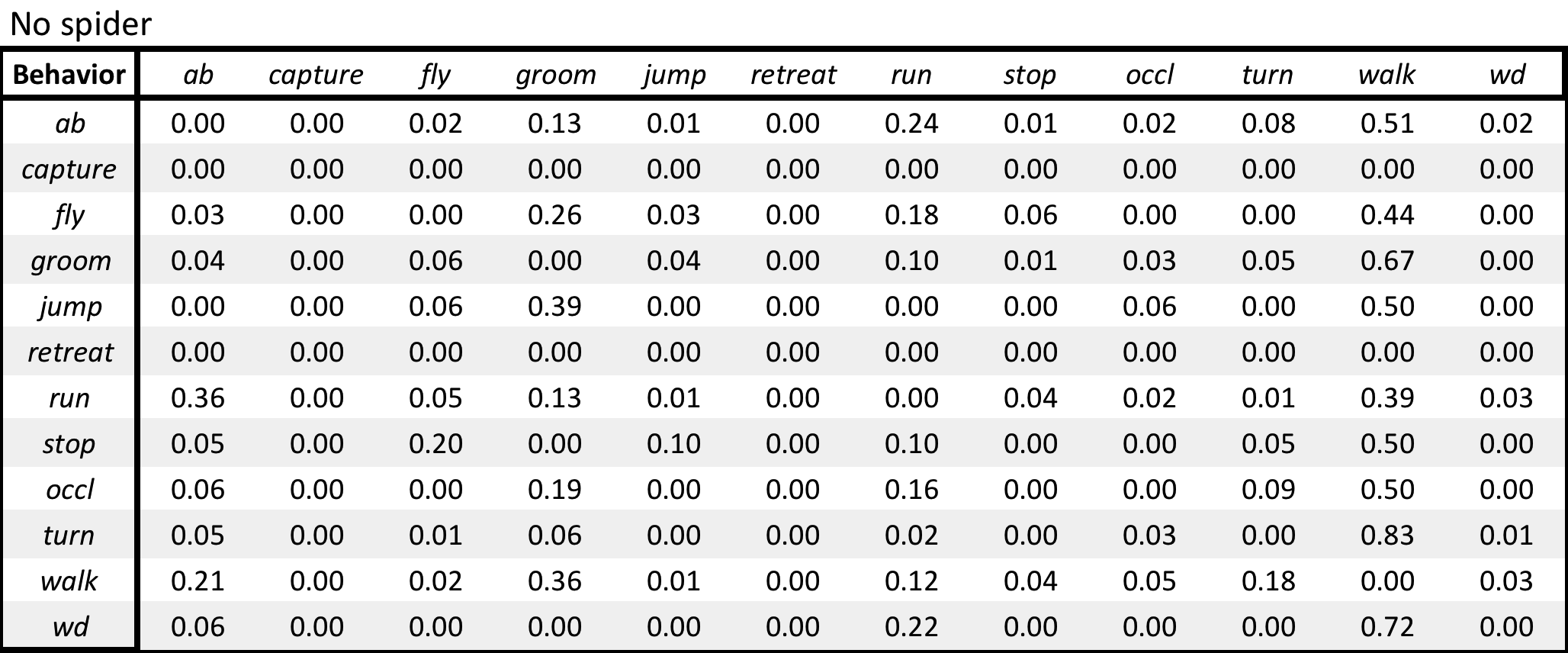


**Table G.** **Transition probability from one behavior (row name) to the other (column name) in the presence of a juvenile praying mantid.** Transition probabilities are obtained by dividing each transition frequency (see S1 table) between a pair of behaviors by the total number of times a given behavior was performed (row sums in S1 Table).


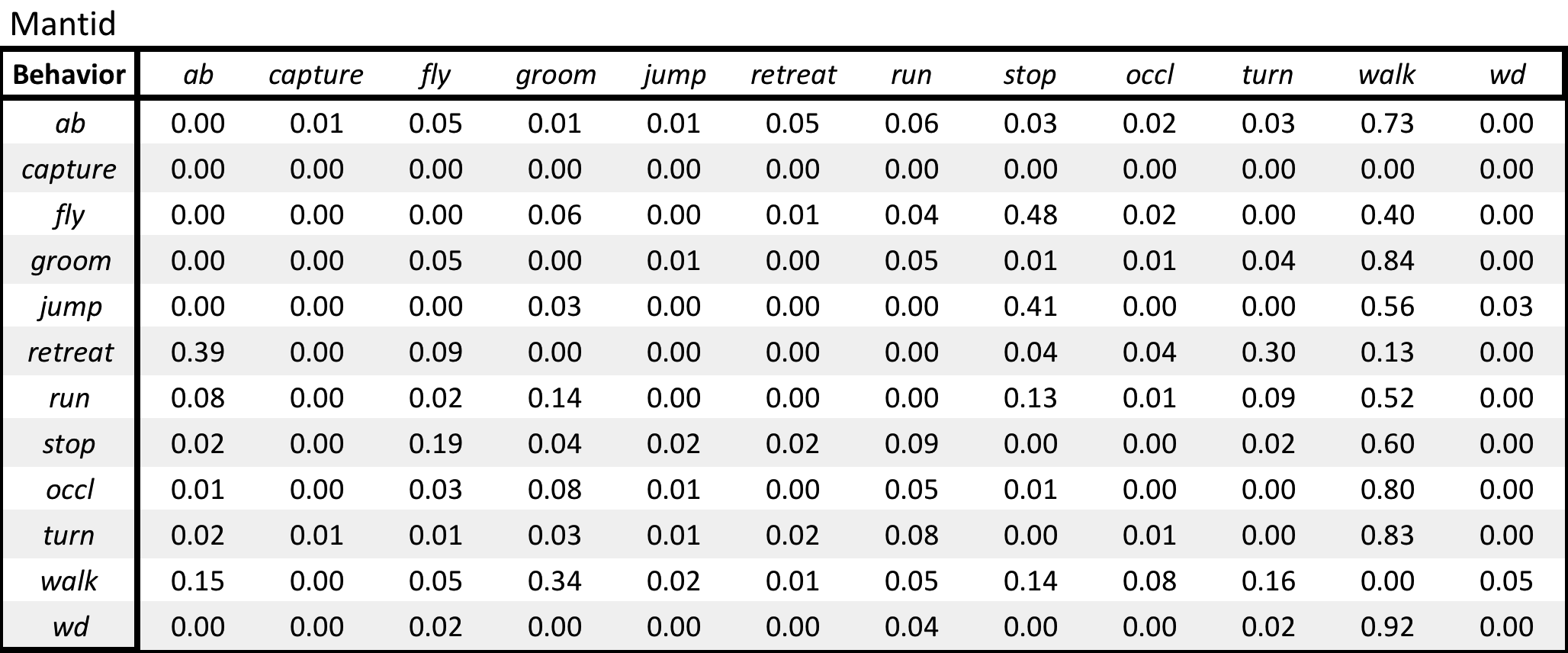


**Table H.** **Transition probability from one behavior (row name) to the other (column name) before a juvenile praying mantid was introduced into the arena.** Transition probabilities are obtained by dividing each transition frequency (see S1 Table) between a pair of behaviors by the total number of times a given behavior was performed (row sums in S1 Table).


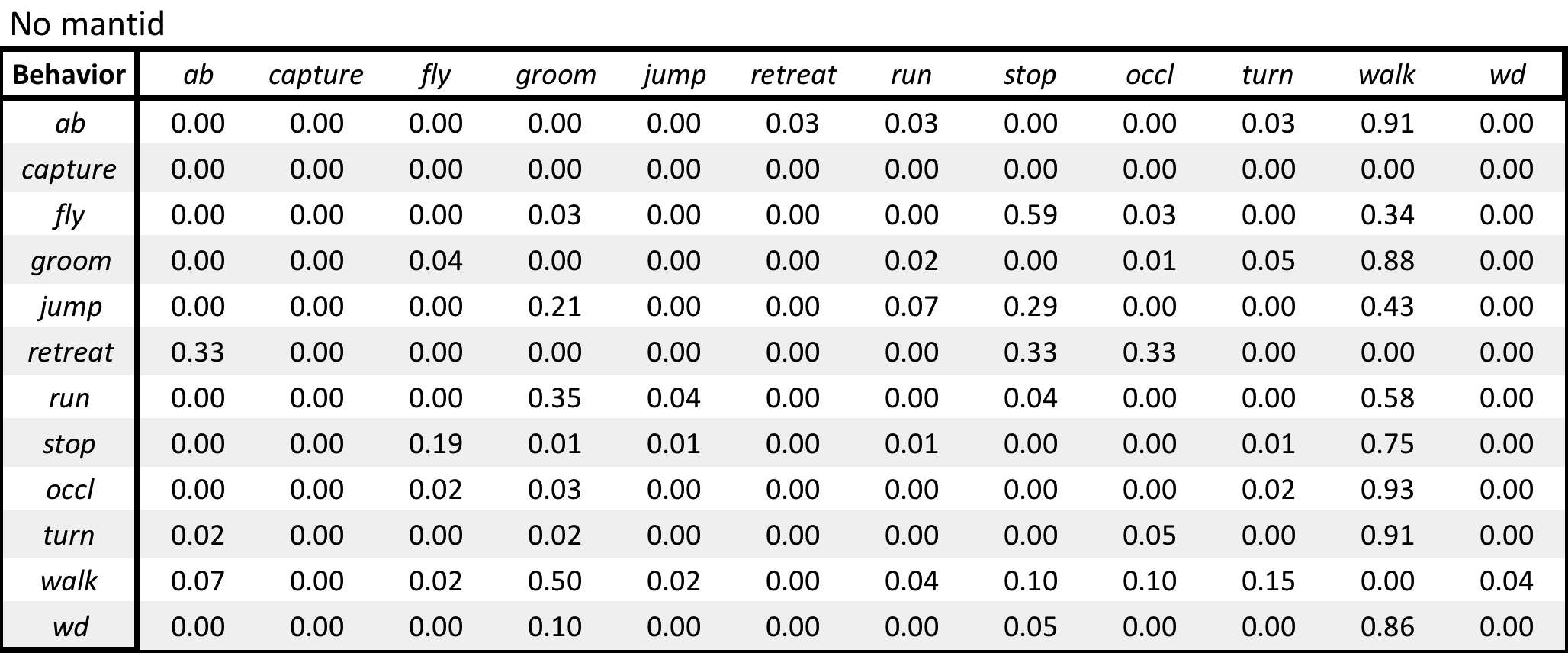


**Table I. Proportion of time spent in a given behavioral state by each individual fruit fly before and after introducing a treatment (i.e., a disturbance, spider or mantid) to the assay chamber.**


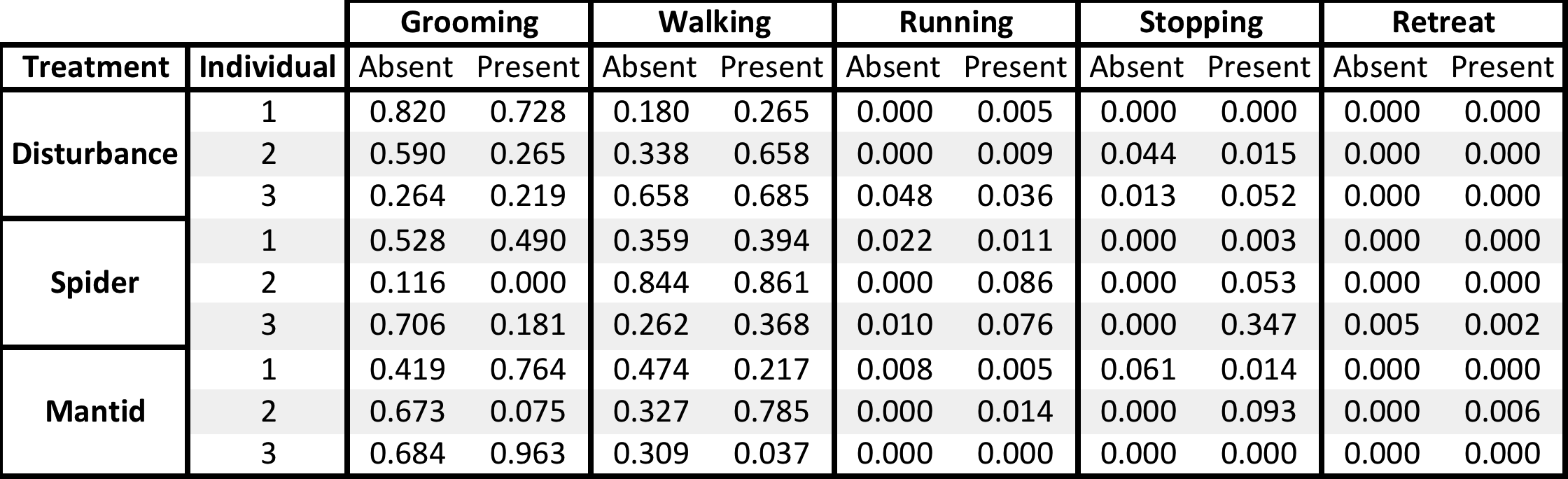


**Table J. Number of occurrences per minute of each behavioral event before and after the introduction of a treatment (i.e., a disturbance, spider or mantid) to the assay chamber.**


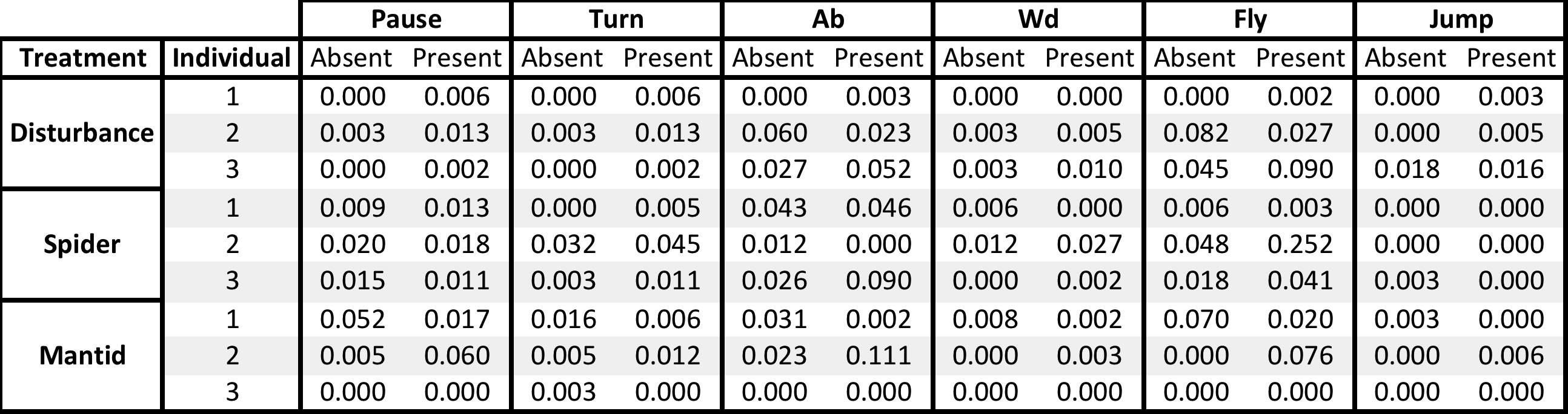


**Table K. Coefficients from a linear model (lm) for control individuals measured before and after the addition of a spider.** While estimate of posterior means are similar to those of the main spider dataset, due to low sample sizes, CIs are large.


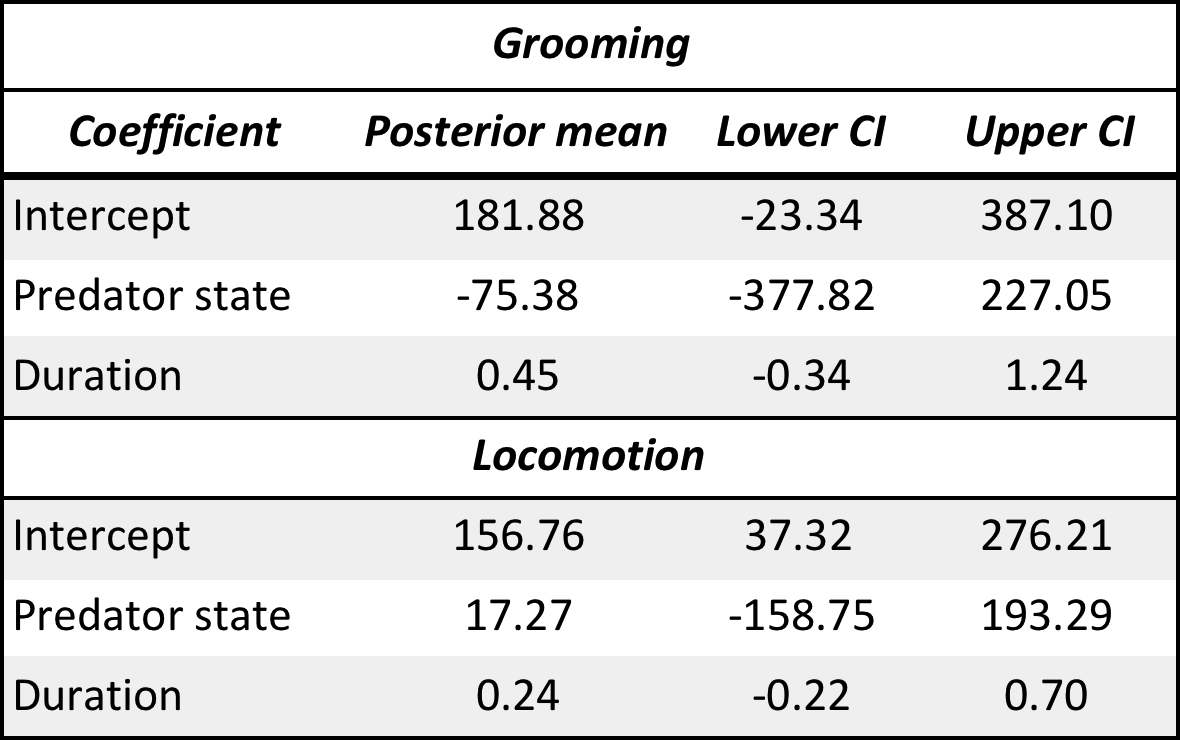


**Table L. Coefficients from a linear model (lm) for control individuals measured before and after the addition of a mantid.** Estimate of posterior means are similar to those of the main mantid dataset, but due to low sample sizes, CIs are large.


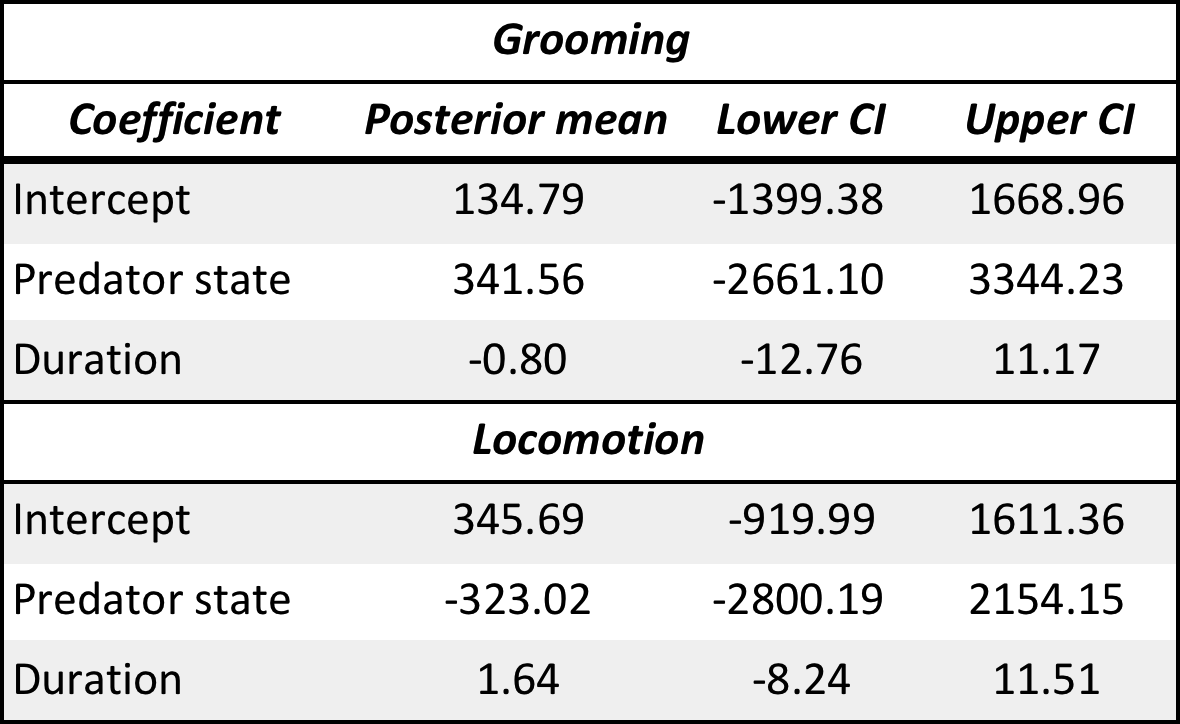


**Table M. Coefficients from a linear model (lm) for control individuals measured before and after a disturbance.** Despite low sample sizes, it is clear that disturbance had minimal effect on fruit fly behaviors.


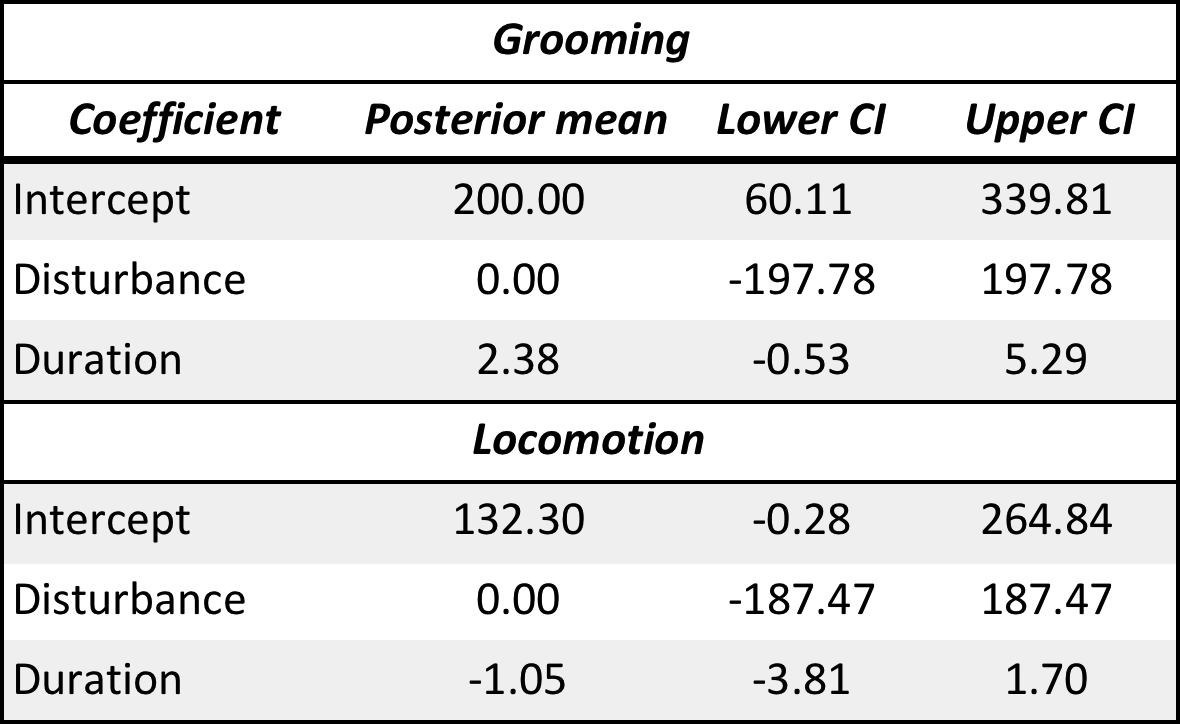


**Links to videos describing novel behaviors**

Video 1) [Abdominal Lifting](http://dx.doi.org/10.6084/m9.figshare.1185638) <http://dx.doi.org/10.6084/m9.figshare.1185638>

Video 2) [Stopping Behavior](http://dx.doi.org/10.6084/m9.figshare.1185639) <http://dx.doi.org/10.6084/m9.figshare.1185639>

Video 3) [Retreat](http://dx.doi.org/10.6084/m9.figshare.1185640) <http://dx.doi.org/10.6084/m9.figshare.1185640>
